# Evaluating a multigene environmental DNA approach for biodiversity assessment

**DOI:** 10.1186/s13742-015-0086-1

**Published:** 2015-10-06

**Authors:** Alexei J. Drummond, Richard D. Newcomb, Thomas R. Buckley, Dong Xie, Andrew Dopheide, Benjamin CM Potter, Joseph Heled, Howard A. Ross, Leah Tooman, Stefanie Grosser, Duckchul Park, Nicholas J. Demetras, Mark I. Stevens, James C. Russell, Sandra H. Anderson, Anna Carter, Nicola Nelson

**Affiliations:** 1 Allan Wilson Centre, University of Auckland, Auckland, New Zealand; 2 Department of Computer Science, University of Auckland, Private Bag 92019, Auckland, 1142 New Zealand; 3 School of Biological Sciences, University of Auckland, Private Bag 92019, Auckland, 1142 New Zealand; 4 The Institute for Plant and Food Research, Private Bag 92169, Auckland, 1142 New Zealand; 5 Landcare Research, Private Bag 92170, Auckland, 1142 New Zealand; 6 South Australian Museum, North Terrace, Adelaide, SA 5000 Australia; 7 School of Pharmacy and Medical Sciences, University of South Australia, GPO Box 2471, Adelaide, SA 5001 Australia; 8 Department of Biological Sciences, University of Waikato, Private Bag 3105, Hamilton, 3240 New Zealand; 9 Department of Statistics, University of Auckland, Private Bag 92019, Auckland, 1142 New Zealand; 10 School of Biological Sciences, Victoria University of Wellington, PO Box 600, Wellington, 6140 New Zealand

**Keywords:** Environmental DNA, Metabarcoding, Biodiversity assessment, Genomic observatory

## Abstract

**Background:**

There is an increasing demand for rapid biodiversity assessment tools that have a broad taxonomic coverage. Here we evaluate a suite of environmental DNA (eDNA) markers coupled with next generation sequencing (NGS) that span the tree of life, comparing them with traditional biodiversity monitoring tools within ten 20×20 meter plots along a 700 meter elevational gradient.

**Results:**

From six eDNA datasets (one from each of 16S, 18S, ITS, *trn*L and two from COI) we identified sequences from 109 NCBI taxonomy-defined phyla or equivalent, ranging from 31 to 60 for a given eDNA marker. Estimates of alpha and gamma diversity were sensitive to the number of sequence reads, whereas beta diversity estimates were less sensitive. The average within-plot beta diversity was lower than between plots for all markers. The soil beta diversity of COI and 18S markers showed the strongest response to the elevational variation of the eDNA markers (COI: *r*=0.49, *p*<0.001; 18S: *r*=0.48, *p*<0.001). Furthermore pairwise beta diversities for these two markers were strongly correlated with those calculated from traditional vegetation and invertebrate biodiversity measures.

**Conclusions:**

Using a soil-based eDNA approach, we demonstrate that standard phylogenetic markers are capable of recovering sequences from a broad diversity of eukaryotes, in addition to prokaryotes by 16S. The COI and 18S eDNA markers are the best proxies for aboveground biodiversity based on the high correlation between the pairwise beta diversities of these markers and those obtained using traditional methods.

**Electronic supplementary material:**

The online version of this article (doi:10.1186/s13742-015-0086-1) contains supplementary material, which is available to authorized users.

## Background

Because of the ease of sampling certain organisms, and the necessity for experts to taxonomically identify sampled organisms, biodiversity assessment typically focuses on a subset of organismal diversity or indicator species. Environmental DNA (eDNA) sequencing technologies now provide a platform for broader biodiversity assessments that do not require complex sampling or expert morphological identification. These methods use next generation sequencing (NGS) technologies to sequence many molecules from the same sample and they have been applied extensively to the assessment of microbial diversity, where the 16S ribosomal gene region is routinely used as a marker to survey microbial diversity [1, 2]. Studies of prokaryote community biodiversity have been undertaken in the human body [3, 4], soils [5–7], freshwater [8], and the deep sea [9].

Increasingly, eDNA monitoring is being used to study eukaryote biodiversity [10–12]. However, these studies have often targeted particular taxa rather than attempting to characterize a full range of biodiversity and few studies have been conducted in terrestrial ecosystems. Those that have taken place in terrestrial systems have most commonly targeted plants by sequencing regions of the chloroplast *trn*L intron [11, 13, 14]. Other studies have targeted the eukaryotic ribosomal gene regions among other gene regions. For example, Creer et al. [15] assessed the biodiversity of meiofauna separated from tropical forest leaf litter by 454 sequencing of 18S sequences, while Bienert et al. [16] attempted to analyse earthworm diversity in soil by targeting short taxon-specific sequences from mitochondrial 16S. Andersen et al. [17] used mammal- and animal-specific primers to characterize the biomass and diversity of large vertebrates based on short DNA fragments extracted from soil. Several studies have used a similar approach to identify a phylogenetically limited range of animal taxa in frozen and ancient substrates [18, 19]. The ITS region has been used to assess palaeobiodiversity of fungi from arctic permafrost [20]. Yu et al. [21] examined the use of NGS to identify arthropods within an artificial mixed ‘soup’ using the COI eDNA marker. Recently, it has also been shown that metabarcoding can be used to obtain similar policy conclusions for restoration ecology and systematic conservation planning to those obtained using standard ecological monitoring techniques [22].

In this study, we attempt to characterize a broad range of biodiversity in a terrestrial system by sampling an elevational series of soils in a temperate forest ecosystem. Soil is the most ubiquitous terrestrial substrate, and in terrestrial ecosystems a rich biodiversity is found in soils and among surface litter, typically exceeding the biodiversity found above ground level [23, 24]. Soil, leaf litter, and the forest floor are home to diverse bacteria, fungi, protists, and metazoans ranging from rotifers, nematodes, earthworms, mites, and beetles to burrowing and surface-dwelling birds, lizards, and mammals. At least 25 % of described animal species reside exclusively in soil and litter layers, and if soil is taken to include substrates such as dung and decaying wood, it is estimated that the majority of terrestrial animal species are soil dwellers [25]. A square meter of surface soil may contain from 10^5^ to 10^8^ invertebrates and 10^7^ to 10^9^ protozoans [23], and a gram of soil may contain from 10^7^ to 10^10^ bacteria [26]. Moreover, molecular evidence has supported the existence of considerably greater soil invertebrate diversity than that indicated by traditional sampling methods [27]. For these reasons, we anticipate that soil will be the most effective single substrate from which to assess biodiversity in a terrestrial ecosystem.

We examined five gene regions (16S, 18S, *trn*L, ITS, COI) to address the following questions; (1) Does eDNA assessment of soil biodiversity offer a useful proxy for traditionally measured aboveground biodiversity? (2) What combination of eDNA markers adequately covers biodiversity? and, (3) How sensitive are the measures of biodiversity to the markers used and parameters used in their analysis? In so doing, we also establish New Zealand’s first contribution to an initiative to build a global network of Genomic Observatories [28, 29].

## Data description

The NGS data and resulting community matrices presented in this paper were collected to assess the utility of a suite of eDNA markers from soil in comparison to estimates of aboveground biodiversity using traditional methods of biodiversity assessment.

**Fig. 1:**
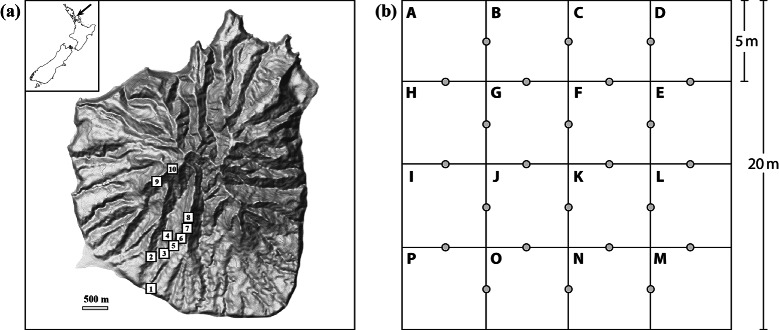
Location and plot details associated with data collection from Hauturu (Little Barrier Island). **a** A contour map with the positions of the 10 plots indicated, and an insert of a map of New Zealand indicating the location of Hauturu. **b** Quadrat design and sampling layout of the 16 subplots (each 5 × 5 m, labelled A-P), with grey circles indicating the positions of the 24 understorey subplots. Each plot had three invertebrate pitfall traps, four lizard pitfall traps, and four lizard cover objects randomly assigned within the 16 subplots. Two subplots were randomly selected for both leaf litter and soil sampling. Bird call stations were located adjacent to each plot

Data were collected to provide both a statistical characterization of biodiversity on a forested island nature reserve and a proof-of-concept of the use of multiple eDNA markers to assess biodiversity over a broad taxonomic range (Fig. 1, Table 1). We collected two types of data: traditional biodiversity data (Table 2) and eDNA data from soil (Table 3). The traditional data consisted of invertebrate, reptile, bird, and vegetation survey data. Invertebrates were isolated from leaf litter samples and collected in pitfall traps. Reptiles were trapped in pitfall traps and under artificial ground covers. Birds were estimated using the distance sampling method [30]. The vegetation data were collected using established national protocols [31, 32], resulting in two separate inventories: (1) tree species counts were carried out across the full plots for all vascular plants with self-supporting stems ≥ 1.35 m tall, and (2) understorey species counts were carried out across 24 circular 0.75 m^2^ subplots, for all vascular plant species ≥1.35 m (Fig. 1). Invertebrate biodiversity was assessed from pitfall traps and leaf litter samples by DNA sequencing of the mitochondrial cytochrome c oxidase subunit I (COI) or barcoding region. Consensus sequences were generated from both strands to ensure high quality. The eDNA data consists of NGS data obtained from PCR products amplified from DNA extracted from either soil (16S, 18S, *trn*L, ITS, COI) or from organism-enriched samples isolated by centrifugation from soil (COI-spun; Table 3).

**Table 1:** Sampling plots. Plots were randomly positioned within 60 m elevational bands and within 200 m linear distance from tracks. Temperatures are annual averages of records taken up to every 30 min at the soil surface between December 2010 and December 2012

Plot name	Latitude	Longitude	Elevation (m)	Mean annual surface
	decimal	decimal		soil temperature
				(degrees Celsius)
Plot 1	-36.22456597	175.069838	50	15.270
Plot 2	-36.21828298	175.070321	90	15.225
Plot 3	-36.21672898	175.073758	160	14.665
Plot 4	-36.21282997	175.074535	260	14.115
Plot 5	-36.21535703	175.075321	240	13.935
Plot 6	-36.21347898	175.075911	320	13.555
Plot 7	-36.21174602	175.078817	420	12.935
Plot 8	-36.21001298	175.078955	460	13.645
Plot 9	-36.20151096	175.071524	595	12.245
Plot 10	-36.19910401	175.075777	640	12.215

**Table 2:** Table of total biodiversity statistics for seedlings, tree, invertebrates, and birds pooled across plots. Numbers of individuals sampled, number of species or invertebrate 97 % OTUs, *α* diversity, effective *α* diversity

	Seedlings	Trees	Invertebrates	Birds	Total
individuals	1302	3520	1406	999	7227
Species/OTUs (97 %)	91	59	413	22	545 ^∗∗^
*α* diversity	24.6	23.6	78.3	12.5	N/C ^∗^
effective *α* diversity	14.3	11.1	44.5	8.6	N/C ^∗^
no. of phyla	1	1	4	1	6

^*^N/C: not calculated

^**^The total number takes the size (110) of the union of species between the seedlings and trees community matrices

**Table 3:** Table of sequence statistics for 16S, 18S, *trn*L, ITS, COI and COI-spun molecular datasets pooled across plots. Number of raw sequence reads, post-QC reads and their unique sequences, chimeras, OTUs at the 97 % threshold, *α* diversity, effective *α* diversity and number of phyla. The quality-control process included error correction of 454 sequence reads using Acacia [34]

	16S	18S	*trn*L	ITS	COI	COI-spun	Total
Raw reads	1,000,881	602,973	1,319,595	377,403	113,427	116,638	3,530,917
Post-QC reads	768,208	539,832	185,314	137,518	84,832	65,786	1,781,490
Post-QC unique sequences	337,849	150,121	105,377	50,166	51,737	25,708	720,958
Filtered reads	563,985	520,826	170,706	132,885	83,747	63,596	1,535,745
Filtered unique sequences	192,151	138,200	100,041	48,118	50,832	23,692	553,034
Chimeras ^+^	147,652	12,130	5,554	2,110	1,013	2,094	170,553
OTUs	15,039	6,440	43,223	6,957	14,248	2,784	88,691 ^∗∗^
Singleton	8,427	2,419	26,537	3,848	6,056	1,497	48,784 ^∗∗^
*α* diversity	3,108.50	1,353.70	4,961.40	935.00	1,786.60	325.40	N/C ^∗^
Effective *α* diversity	295.22	69.48	1,293.63	77.77	631.77	17.23	N/C ^∗^
No. of phyla ^++^	43	58	49	35	60	31	109

^*^N/C: not calculated

^**^The total number of OTUs is just each number for each gene added together

^+^The total number of unique sequences that were classified as chimeras by both USEARCH OTU clustering and UCHIME in UPARSE pipeline

^++^The number of phyla includes NCBI-defined phyla and equivalent high-level taxa

The results of the vegetation surveys have been deposited in the National Vegetation Survey Databank (Landcare Research). Bird call counts, soil chemistry, elevation, and temperature data have been deposited in GigaDB [33]. No reptiles were caught in either the live pitfall traps or under artificial covers during the sampling period. Sanger sequences of invertebrates (*n*=1,720) have been deposited in GenBank with their New Zealand Arthropod Collection codes (GenBank accession numbers KP420745-KP422464). Environmental DNA sequences have been deposited in the NCBI Sequence Read Archive (Project Accession: PRJNA267737). An overview of the project can be found at the New Zealand Genomic Observatory Data Warehouse (http://data.genomicobservatory.cs.auckland.ac.nz).

## Analyses

Deconvolution, trimming, and quality-based filtering of the NGS data from the 16S, 18S, *trn*L, ITS, COI, and COI-spun eDNA datasets resulted in 65,786-768,208 high quality reads per marker. Error-correction of the sequence reads was performed using Acacia [34]. Operational taxonomic units (OTUs) by eDNA marker were determined using the UPARSE [35] pipeline with a 97 % sequence similarity clustering threshold (in all cases except Fig. 4 where we vary the sequence similarity threshold). Additionally, an alternative set of OTUs for each amplicon dataset was constructed in which all of the single-read OTUs were removed (see Additional file 1 for a full set of parallel analyses to match those described below, none of the major conclusions are affected by this alternative data treatment). 

**Fig. 2:**
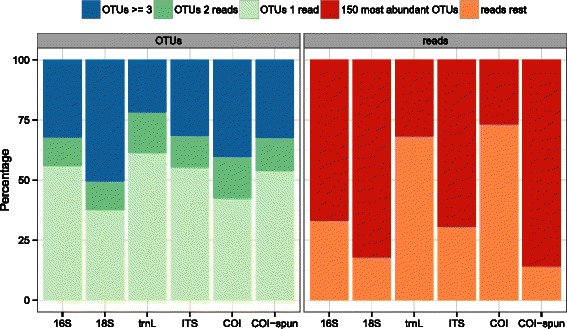
Relative proportion of OTUs at 97 % clustering threshold inferred by read count for molecular datasets. *Left* panel: Percentage of OTUs having 1 read (‘OTUs 1 read’), 2 reads (‘OTUs 2 reads’), and 3 or greater reads (‘OTUs ≥3’). *Right* panel: Percentage of reads in the most abundant 150 OTUs (‘150 most abundant OTUs’), compared to all remaining reads ‘reads rest’)

**Fig. 3:**
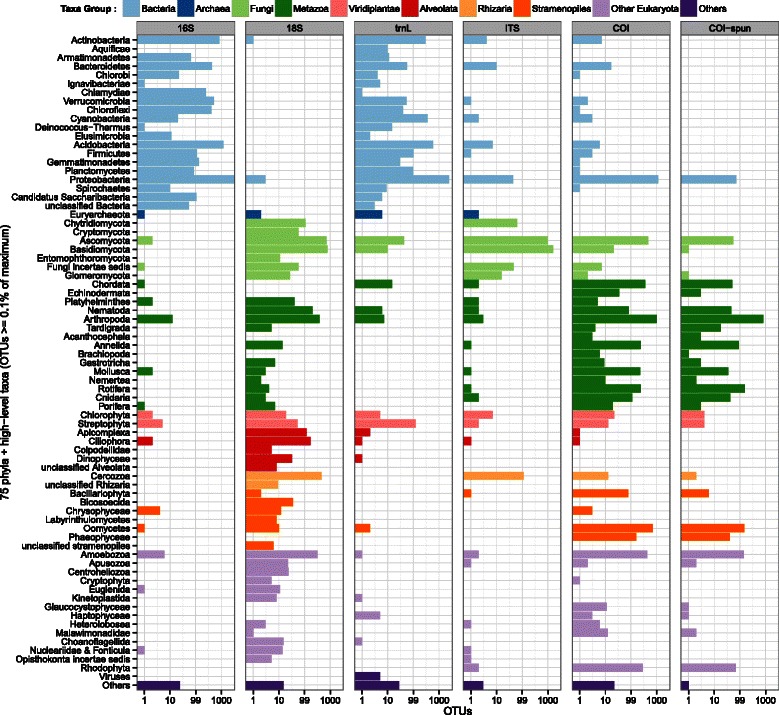
The number of OTUs at the 97 % clustering threshold assigned to phyla. Unclassified OTUs and OTUs containing low-complexity sequences are not included, OTUs from phyla that are represented by less than 0.1 % of the OTUs are grouped into the ‘Others’ category

OTUs were assigned to phyla using BLAST+ and MEGAN 5 [36] (Figs. 2 and 3).

**Fig. 4:**
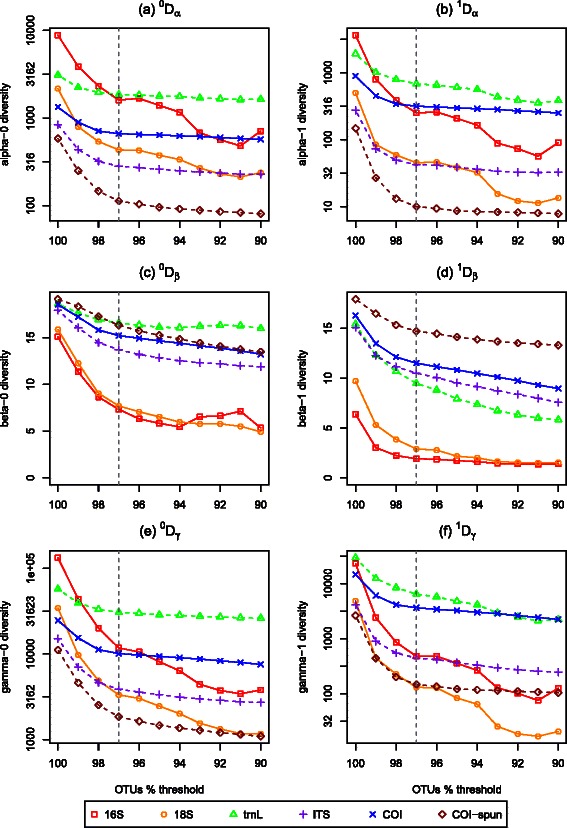
Plots of diversities using cutoff thresholds ranging from 90–100 % for OTU classification of **a** *α* diversity, **b** effective *α* diversity, **c** *β* diversity, **d** effective *β* diversity, **e** *γ* diversity, and **f** effective *γ* diversity. Molecular datasets include 16S, 18S, *trn*L, ITS, COI, and COI-spun

Diversity statistics were calculated for both eDNA marker datasets (Table 3) and those collected using conventional methods (Table 2) with the R package *vegetarian* [37]. Alpha, beta, and gamma diversities all decreased steeply as the similarity threshold for OTU clustering decreased from 100 to 97 %. The diversities were generally less sensitive to changes in the similarity threshold between 90–97 % (Fig. 4). Beta diversities were less sensitive to the choice of OTU similarity threshold than the alpha and gamma diversity estimates.

**Fig. 5:**
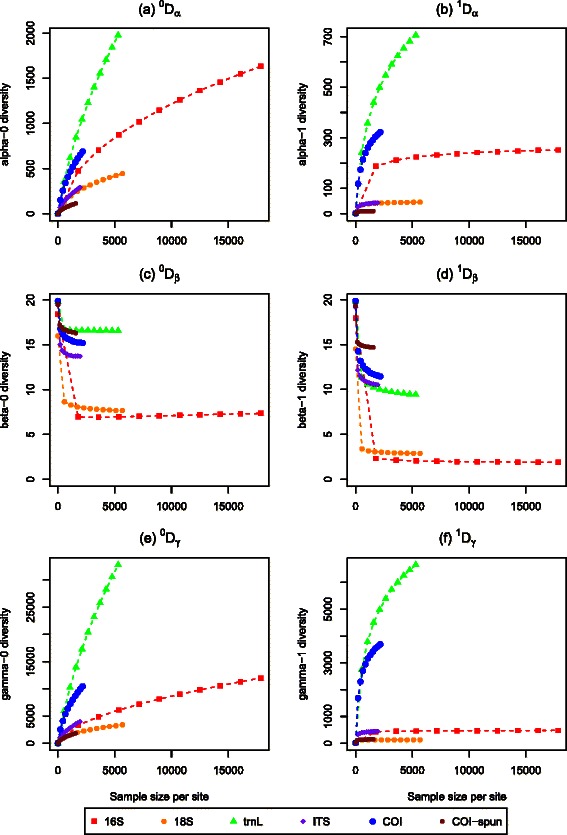
Rarefaction curves for diversities estimated using a 97 % threshold for OTU classification of **a** *α* diversity, **b** effective *α* diversity, **c** *β* diversity, **d** effective *β* diversity, **e** *γ* diversity, and **f** effective *γ* diversity. Molecular datasets include 16S, 18S, *trn*L, ITS, COI, and COI-spun

**Fig. 6:**
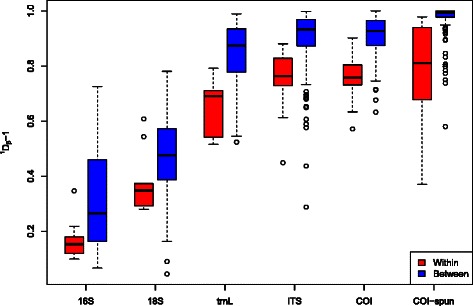
Box and whisker plots of turnover (normalized pairwise effective *β* diversity) within (*red*) and between plots (*blue*) for the molecular methods 16S, 18S, *trn*L, ITS, COI, and COI-spun

Rarefaction curve analysis for each of the eDNA markers indicates different sampling properties for the different diversity statistics (Fig. 5). Measures of alpha and gamma diversities were highly dependent on the number of sequences, with most gene regions not asymptoting to a maximum. On the other hand, beta diversities trended towards a stable measure after a few thousand sequence reads for all the eDNA markers examined. Beta diversities within and among plots varied for the different markers (Fig. 6). Beta diversities were low within plots for 16S, but were highly variable between pairs of plots.

**Fig. 7:**
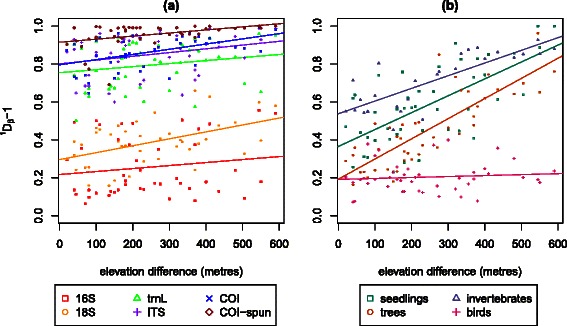
Regression of effective *β* diversity and difference in elevation for **a** the eDNA biodiversity datasets (16S, 18S, *trn*L, ITS, COI-soil and COI-soil spun) and **b** traditional biodiversity datasets (seedlings, trees, invertebrates, birds)

The 18S marker showed intermediate levels of beta diversities, both within and between pairs of plots, whereas the remaining four eDNA markers had high beta diversities within and especially between pairs of plots. A regression analysis of pairwise beta diversity against the elevational difference between plots (Fig. 7) shows that among the conventional methods, trees, seedlings and invertebrates have the strongest positive correlation. This decrease in compositional similarity with increasing elevational separation is analogous to the well-established distance-decay relationship [38, 39]. Among the eDNA markers, the COI and 18S markers showed the strongest positive correlation between pairwise beta diversity and elevational difference (COI: *r*=0.49, *p*<0.001; 18S: *r*=0.48, *p*<0.001). All of the correlations were significant using PERMANOVA [40] except 16S and *trn*L (Table 4).

**Table 4:** Mantel statistic *r* and their significance using Mantel’s test based on 4,999 permutations, and R^2^ and *p*-*value* for a linear model of the regression of effective *β* diversity and difference in elevation in Fig. 7

	Mantel statistic *r*	Significance	R^2^	*p*-*value*
16S	0.155	0.2048	0.0241	0.309
18S	0.481	0.0042	0.231	8.35e-04
trnL	0.201	0.1378	0.0402	0.187
ITS	0.276	0.0524	0.0763	0.0662
COI	0.487	0.0034	0.237	6.9e-04
COI-spun	0.398	0.0026	0.158	0.00683
Seedlings	0.672	8e-04	0.451	4.37e-07
Trees	0.827	2e-04	0.684	2.51e-12
Invertebrates	0.813	0.0052	0.661	1.48e-07
Birds	0.096	0.2942	0.00929	0.529

### Soil eDNA markers as proxies for traditional biodiversity assessment methods

Pairwise community correlations form a matrix describing the correlations among and between the traditional and eDNA community samples (Table 5). This analysis shows which methods have the strongest correlations between pairwise beta diversity measures. The strongest correlation between an eDNA method and a traditional method was found between the COI eDNA dataset and the conventionally collected invertebrates dataset (*r*=0.80; *p*<0.001; Table 5). COI eDNA beta diversities were also strongly and significantly correlated with vegetation pairwise beta diversities (*r*=0.69 for seedlings and *r*=0.61 for trees).

**Table 5:** Pairwise community matrix correlations of effective *β* diversity within and between the eDNA datasets and traditional datasets, Mantel statistic *r*, and their significance in parentheses using Mantel’s test based on 4,999 permutations

	16S	18S	trnL	ITS	COI	COI-spun	Seedlings	Trees	Inverts
18S	0.484 (0.0034)								
trnL	0.801 (2e-04)	0.59 (2e-04)							
ITS	0.431 (0.02)	0.588 (2e-04)	0.618 (2e-04)						
COI	0.642 (2e-04)	0.593 (2e-04)	0.79 (2e-04)	0.616 (4e-04)					
COI-spun	0.342 (0.0318)	0.558 (2e-04)	0.399 (0.012)	0.611 (4e-04)	0.453 (0.0038)				
Seedlings	0.499 (0.0464)	0.542 (0.003)	0.469 (0.0328)	0.482 (0.0032)	0.685 (2e-04)	0.427 (0.0036)			
Trees	0.259 (0.1004)	0.551 (0.001)	0.317 (0.0622)	0.398 (0.0118)	0.611 (2e-04)	0.43 (0.0022)	0.816 (4e-04)		
Inverts	0.322 (0.0988)	0.694 (0.0014)	0.5 (0.03)	0.504 (0.0036)	0.802 (4e-04)	0.611 (0.0064)	0.827 (0.0018)	0.827 (0.004)	
Birds	-0.02 (0.4194)	0.29 (0.0934)	0.1 (0.3024)	0.124 (0.2384)	-0.135 (0.6908)	0.021 (0.4132)	-0.046 (0.5122)	-0.024 (0.4714)	-0.031 (0.5126)

These correlations are summarized in a second-stage MDS that provides an ordination of the methods by their similarity of pairwise beta diversities (Fig. 10). This shows that the COI and 18S methods are the closest eDNA methods to the traditional measures (seedlings, trees, invertebrates). The bird dataset was excluded for better visualisation because it was independent from the other datasets (see Table 5). The full plot is available as Figure SA13 in Additional file 2. 

A comparison of plot rank importance based on maximizing retained beta diversity also showed some correlations between eDNA and traditional biodiversity measures, but this comparison was less definitive owing to the small number of plots and weak power of the Spearman’s rank correlation test. The strongest correlation in ranked importance between an eDNA and traditional biodiversity measure was found between COI and invertebrates (*r*=0.76; *p*<0.005; see Additional file 2). The ranking of plots based on 16S beta diversity were highly correlated with those based on seedling community data (*r*=0.75; *p*<0.02).

### Comparing communities across samples

**Fig. 8:**
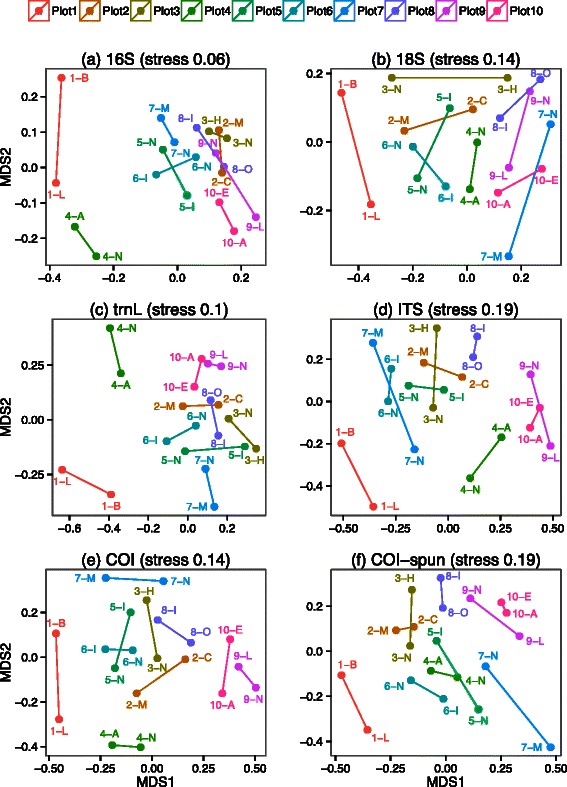
Non-metric multidimensional scaling of effective *β* diversity for paired subplots for the molecular datasets 16S, 18S, *trn*L, ITS, COI-soil, and COI-soil spun

Non-metric multidimensional scaling plots based on effective beta diversity generally show consistent differentiation of samples based on elevation for all amplicon datasets (Fig. 8). The largest differences are observed between the lowest elevation samples (Plot 1) and the highest elevation samples (Plots 8, 9, and 10). The communities in Plots 5, 6, and 7 generally have intermediate similarity between the lowest and highest elevation samples, whereas the communities in Plot 2 and Plot 3 samples tend to be more similar to those in high elevation Plot 8 samples. Plot 4 samples show the most variation between amplicons, being most similar to mid-elevation samples for 18S and COI-spun, and to Plot 1 samples for 16S, but having limited similarity to all of the other samples for *trn*L, ITS, and COI. Similar patterns were observed in ordination plots based on Jaccard and Horn-Morisita indices (Figures SA11 & SA12 in Additional file 2).

**Fig. 9:**
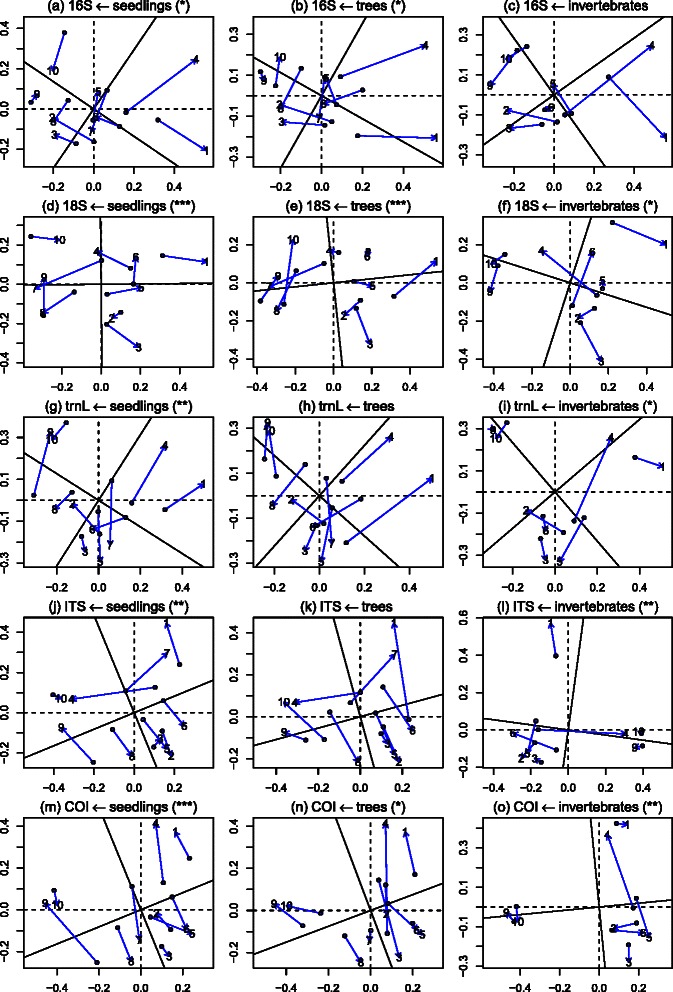
Procrustes analysis of effective *β* diversity between the eDNA datasets and traditional datasets, and their significance level in parentheses is estimated based on 4,999 permutations

To more precisely compare the similarities in ordination across methods, Procrustes comparisons were made between the eDNA methods and the traditional methods (excluding birds) (see Fig. 9). These comparisons show that 18S and COI have significant similarities in their ordination to all three traditional methods (seedlings, trees, invertebrates). Other eDNA methods show lesser degrees of similarity with traditional methods. This reflects the Mantel test results in a reduced-dimension context.

**Fig. 10:**
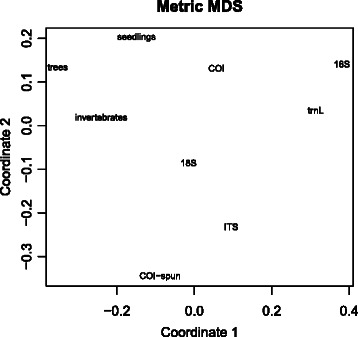
Multidimensional scaling (no birds) of pairwise community matrix correlations of effective *β* diversity within and between the eDNA datasets (16S, 18S, trnL, ITS, COI-soil, and COI-soil spun) and traditional datasets (seedlings, trees, invertebrates)

### Environmental drivers of biodiversity patterns

Fifteen abiotic environmental variables were examined for their ability to explain the patterns of biodiversity in the amplicon datasets using distance-based redundancy analysis [41, 42]. The *sin.aspect* and *cos.aspect* variables were derived by taking, respectively, the *sine* and *cosine* of the aspect measurement in degrees to enable inclusion of these data in distance-based redundancy models. Values for *sin.aspect* (indicating how *east-facing* a plot is) and *cos.aspect* (indicating how *north-facing* a plot is) range from -1 (representing south and west) to 1 (representing north and east).

When each of the environmental variables were tested in isolation, the highest proportions of explained inertia were observed for the 16S dataset and the lowest for the COI-spun dataset (Tables SA17 and SA18 in Additional file 2). For the 16S dataset, 18.8 % of inertia was explained by pH, 13.5 % by phosphorus, and 12.2 % by electrical conductivity, 11.4 % by organic carbon, and ≥ 10 % for soil water content, total nitrogen, and NH_4_. For the 18S dataset, 9.11 % of inertia was explained by elevation and 8.95 % by water content, and for the *trn*L dataset 8.06 % was explained by pH. Aside from the 16S and 18S dataset, the proportions of inertia explained by all other variables in all datasets were typically in the range of 5 % to 8 %. The set of nine variables with high variance inflation factors (VIF) <10 together explained from 50.5 % (COI) to 61.3 % (16S) of inertia (Table SA19 in Additional file 2), but in each case only a subset of these variables were significant according to permutation tests. Slope, temperature, cos.aspect, sin.aspect, and pH were significant (or near-significant) for 16S, *trn*L, COI and COI-spun datasets, in addition to NO_3_ and NH_4_ for COI-spun. For the 18S dataset, slope, temperature, pH, C/N ratio, and NH_4_ were significant (or near-significant), as were slope, temperature, sin.aspect, and phosphorus for the ITS dataset. Ordination biplots suggest that temperature and pH tend to have an influence on the community composition in a similar direction, which differs from that of the other soil chemistry variables/slope/cos.aspect (Figure SA19 in Additional file 2).

Backward selection of variables resulted in from three to six variables for each dataset, explaining from 25.3 % (ITS) to 40.8 % (18S) of inertia, whereas forward selection of variables resulted in only two to four significant variables, explaining from 14 % (ITS and COI) to 37.4 % (16S) of inertia, which were usually a subset of the corresponding backward selection model variables (Table SA17, SA18 & SA19 in Additional file 2). Phosphorus was included in forward and backward selection models for all datasets (except the COI-spun backward selection model). Forward and backward selection models for 16S, *trn*L and COI datasets also included pH, and temperature occurred in both models for 16S but only backward selection models for *trn*L and COI, in addition to slope and cos.aspect (*trn*L), or NO_3_ and NH_4_ (COI). For the 18S and ITS datasets, forward selection models contained only phosphorus and slope, whereas the corresponding backward selection models also contained pH and temperature (18S), or sin.aspect (ITS). Models for the COI-spun dataset included North and East measurements, as well as phosphorus (forward selection model), or temperature, slope, NO_3_, and NH_4_ (backward selection model). Ordination biplots of forward/backward selection models show that 16S, *trn*L and COI assemblages in Plots 1 and 4 are associated with elevated levels of soil pH and phosphorus, whereas communities in the highest elevation plots (Plots 9 and 10) are associated with elevated phosphorus but lower soil pH and temperature levels (Figure SA19 & SA20 in Additional file 2). ITS and 18S communities in Plots 4, 9, and 10 are associated with elevated phosphorus and slope according to forward selection models. According to backward selection models, 18S communities in Plot 1 are also associated with lower temperature and pH, whereas ITS communities are also associated with increasing sin.aspect (Plots 2 and 3) and NO_3_ levels (Plots 9-N and 10-E). COI-spun communities in Plots 2 and 3 are also associated with increasing sin.aspect, whereas Plot 8 is associated with decreasing sin.aspect, and Plots 9 and 10 with increasing cos.aspect, NO_3_ and slope, and reduced temperature.

A subset of nine of the above abiotic environmental variables were examined as potential drivers of the patterns in vegetation community datasets using distance-based redundancy analysis [41, 42]. The smaller number of variables used was due to the limited number of plots in this pilot study. For the seedling dataset, 17.1 % of inertia was explained by pH, and 16.1 % of by NO_3_. They were both significant in the corresponding forward/backward selection models. For the trees dataset, 23.4 % of inertia was explained by temperature, and 21.4 % of by pH. There was, however, no single variable chosen in either the forward or backward selection models (Figure SA21, Table SA20 and SA21 in Additional file 2).

## Discussion

We have demonstrated that, by using standard barcoding primers on eDNA extracted from soil, we are able to broadly sample taxa from the soil biota. The majority of the sampled phyla are known to be found in soil, including, for example, Eubacteria, Amoebozoa, Basidiomycetes and Arthropoda. There are also a few unexpected phyla that may be artefacts of the bioinformatics pipeline (e.g. the small number of OTUs identified as members of Porifera and Echinodermata are probably due to errors in the taxonomic identification of matching sequences in GenBank). This finding extends the use of eDNA methods in soil beyond the commonly used bacterial 16S eDNA paradigm to include the majority of eukaryotic groups. Of the eukaryotic eDNA markers, COI recovered the most phyla (60), followed by 18S which recovered 58 phyla, with fewer found by *trn*L (49), especially as most of the phyla from *trn*L were prokaryotic (see below).

At least two eDNA markers are required to cover a majority of the phyla, one covering the prokaryotes and at least one other for the eukaryotes. The 16S eDNA marker is an obvious choice for the prokaryotes due to the large amount of comparative data held in reference datasets and databases. Which eDNA marker is optimal for the eukaryotes is less clear and may depend more on the groups of interest and desired taxonomic resolution. The COI eDNA primers used here recover a similar number of phyla to 18S, but with a greater number of phyla represented from within the metazoa. The 18S eDNA primers used here cover a broader range of eukaryotic taxa from single-celled organisms, including alveolata and rhizaria, to fungi and metazoa. The 18S marker is highly conserved [43] and at the 97 % cutoff level will probably often lump closely related species and genera into single OTUs. If finer-scale measures of eukaryotic diversity are required, for example species, then other eukaryotic markers should be included, such as COI for metazoa and ITS for fungi. Although we did find that ITS targets fungi almost exclusively, it did contain significant length differences, making it difficult to align confidently compared to the other markers. The *trn*L marker was intended to sample Viridiplantae because this marker is routinely used as a molecular barcode for plants [44, 45]. However, when using it as an eDNA marker with NGS, most of the resulting reads were prokaryotic in origin (Fig. 3). The primers that we used for *trn*L will require refinement for application in metabarcoding of vascular plants from soil. The two different COI methods resulted in very similar distributions of phyla being sampled.

All of the measures of biodiversity from the different eDNA methods are sensitive to OTU sequence similarity cutoff thresholds. Consistency in the use of a cutoff level will be important for measuring alpha and gamma diversity, although these are less important for beta diversity. The 97 % sequence similarity level appears to lie near an apparent inflection point on most of the diversity measure curves. Stable estimates of alpha and gamma diversity levels require deep sequencing, regardless of marker, whereas stable estimates of beta diversity from eDNA can be obtained from a few thousand sequences from any one of the markers.

The six eDNA datasets consistently return different absolute measures of biodiversity. The *trn*L marker consistently gives the highest levels of species diversity (alpha and gamma), whereas COI-spun and ITS give the lowest levels of alpha, beta, and gamma diversity. The low levels of alpha diversity in the COI-spun is likely to be due to the reduction in the number of prokaryotic and single-celled eukaryotic sequences relative to the COI dataset. The overall difference in biodiversity measures among the eDNA methods can be explained in part by a simple consideration of the physical size and density of these very different organisms. Even very small invertebrates of sub-millimeter length (e.g. nematodes, rotifers, mites) have an individual biomass of at least six orders of magnitude greater than that of a typical soil bacterium. This radical increase in biomass leads to a similarly large reduction in the density of multicellular animals in a given volume of soil. This is perhaps best demonstrated by the higher estimates of beta diversity between plots for larger organisms, which is also suggestive of a link with scaling. The scaling laws of organism size lead to natural consequences for biodiversity in a given volume. This simple explanation has natural yet non-trivial consequences for decisions about sampling and DNA extraction protocols, which should be the focus of future research. The challenge in assessing biodiversity across a wide taxonomic range from environmental samples such as soil is to choose a sampling strategy that provides the best outcome for comparing diversity and its change across the landscape. A sufficient overlap must exist in the sampled communities at different locations if such a comparison is to be possible. The optimal volume of soil from which to extract DNA may vary by orders of magnitude for different taxonomic groups, even among the ‘very small’ species. Ground-truthing these biodiversity measures against reference sets that have been morphologically identified as belonging to particular taxonomic species will be important for assessing the absolute measures and is the subject of future publications from this study. Furthermore, current sequencing technologies are dominated by Illumina technology and, although transferring to this approach will be essential in the future, such a change will not affect the main conclusions of this study. Despite these limitations, our research demonstrates the feasibility of using multiple eDNA markers to assess soil biodiversity from all of the major branches of the ‘tree of life’ and predict patterns of aboveground biodiversity using these measures.

The eDNA sequences analyzed in this study allowed us to estimate the biodiversity within sample plots across a broad range of taxa. This provides a basis for classical comparisons of communities, investigation of the factors that drive community differences, and assessment of priorities for conservation. The patterns of multivariate community similarity observed between samples were broadly similar among the different amplicon datasets, suggesting comparable responses of different taxonomic groups to the elevation gradient from which the samples were collected. Furthermore, there were consistencies among the sets of abiotic variables that were associated with patterns of community similarity, which may indicate that common physical/chemical factors are influencing the composition of the following subgroups of the sampled communities: 16S/*trn*L/COI, 18S/ITS, and COI/COI-spun. This seems reasonable because the 16S and *trn*L datasets both contain mainly bacterial sequences, and the COI dataset contains a significant minority of bacterial sequences. The 18S and ITS datasets both include many fungal sequences, and the COI and COI-spun communities both include many metazoan sequences.

Finally, we have demonstrated that, of the amplicon datasets that we investigated, the COI and 18S markers were most similar to traditional methods (vegetation surveys and invertebrate collections) in their pairwise plot beta diversities and ordination of plots by community similarity. These two markers thus represent the best proxies for traditional biodiversity assessments of those that we investigated. Further studies that expand the sample size and landscapes investigated should enable an even better understanding of the properties of these promising next generation biodiversity assessment tools.

## Methods

### Plots and field sampling

#### Field site

Plots were established on Hauturu-O-Toi (Little Barrier Island), which is one of New Zealand’s largest temperate off-shore island sanctuaries (36.19S, 175.11E), in December 2010 (Table 1, Fig. 1). Hauturu is a protected restricted access nature reserve; it is 3,083 ha in area and it rises to 722 m above sea level [46]. The dormant volcanic island is heavily forested, with over 400 species of native plants [47], and it is home to the most locally diverse assemblage of native vertebrates in New Zealand, with over 40 species of birds, two species of bat, and 14 species of reptiles. Although it provides the best opportunity to evaluate a pre-human ecosystem free from introduced browsing mammals, it has had introduced mammalian predators - cats (*Felis catus*) and Pacific rats (*Rattus exulans*), which are now eradicated - and the forest has been modified in parts by historical logging and fire prior to 1895.

#### Plots

Ten 20 ×20 m plots were established using standard protocols for vegetation community analyses [31, 32, 48]. Each 20×20 m plot was divided into 16 5×5 m subplots labelled A-P, with M-P located along the higher contour line and A-D the lower. Locations for the P corner of plots were randomly generated within 200 m distance along a contour off a specified track, with one plot for each 60 m elevational band. The track was predetermined based on accessibility in most weathers and the relatively intact vegetation representative of the original state of the island throughout all of the elevational sections. Random sites were discarded if the slope was >50 degrees and, therefore, the survey work would be destructive to the site or would be considered unsafe; in either instance, further random sites were targeted. All of the targeted random sites for the high elevational sections for Plots 9 and 10 on the same track were unsafe for survey work, so random sites off the south facing ridge-line of the nearest track were targeted for these plots. A 200 m limit was set to enable plots to be visited within logistical constraints and to ensure that the plots could be located anywhere from a ridge-line to a stream gully (i.e. sampling was not biased towards a ridge-line due to the track location).

#### Plant sampling and mapping

The vegetation data were collected using two separate inventory protocols, as outlined previously [48]: (1) tree species counts and (2) understorey species counts. Tree species counts involved sampling the full plots for all vascular plant individuals with self-supporting stems ≥ 1.35 m tall. All trees with a diameter at breast height (DBH) ≥ 25 mm were identified to morphospecies, measured and given permanent tags. Each individual was recorded at the subplot level (A-P). Understorey species counts were carried out across 24 circular 0.75 m^2^ subplots, for all vascular plant species <1.35 m tall (see Fig. 1 for placement).

Analyses of the tree data used raw abundance measures (based on full plot stem counts), while analyses of the understorey data used presence-absence observations (based on subplot presences, with each recorded species given a value of 1–24 for each plot). To simplify the nomenclature, all of the components of the understorey subplots are referred to as ‘seedlings’ in the Tables and Figures – although these subplots also recovered mature plants of small stature (e.g. many ferns and lycophytes).

#### Invertebrate sampling

Pitfall traps (100 mm diameter, 680 ml plastic containers) containing approximately 200 ml 100 % propylene glycol were placed in three randomly allocated subplots per plot for 5–7 days (Plots 1–8 for 7 days; Plots 9 and 10 for 5 days). Pitfall traps were then removed and the fluid and contents were transported to Landcare Research, Auckland, where the material was transferred into 100 % ethanol. At the same time, 2 kg leaf litter samples were taken from each of two randomly allocated subplots per plot, placed into cloth bags, and taken to Landcare Research, where they were placed in Berlese funnels (Landcare Research, Auckland, New Zealand) for 1–2 weeks. Invertebrates were collected into 100 % ethanol from the Berlese funnels and then separated into Arthropoda, Collembola, and Acari by an expert entomology technician. Each specimen was given a unique barcode label from the New Zealand Arthropod Collection (Landcare Research, Auckland). Selected specimens, representative of taxonomic diversity, were imaged using an Auto-Montage System (Syncroscopy, United Kingdom).

#### Bird counts

At each plot, hourly counts were made between 0900–1200 and 1400–1700 h to provide an estimate of bird species richness and abundance at the site. Birds were identified and counted based on their sighting and calls by a single expert ornithologist using the 5 min point-count distance survey method [30]. A species list of all birds seen or heard during the 3-h sessions at each plot was also recorded, as well as data on an ordinal scale of 0–5 for wind, noise, sun and precipitation.

#### Reptile sampling

Live capture pitfall traps were installed for lizards in four randomly allocated subplots throughout Plots 1–8. Traps were not installed in Plots 9 and 10 because these were located on another track and they could not be checked daily (a requirement of the ethics permit) owing to logistical constraints. The traps were 4 l buckets with drainage holes, installed so the lip was flush with the soil surface, with plastic lids set slightly above the trap using wire stands. Traps were baited with tinned pear, and they had a bed of leaf litter and a damp sponge inserted in the bottom. The traps were checked daily for 7 days, the bait was refreshed, and the sponge was moistened daily. Artificial cover objects were installed in four randomly allocated subplots in all ten plots. These consisted of brown Onduline (corrugated bitumen used for roofing) sheets, 670 × 420 mm, placed on top of the leaf litter. The covers were checked eight times over 3 months.

#### Soil sampling

Soil (1 kg) was collected from each of the same two subplots as the leaf litter samples. Soil was collected to measure the soil chemistry and environmental DNA, with sterile gloves and trowels. The trowel was wiped down with ethanol after each collection and the gloves were changed between subplots. The soil was kept cool and as soon as possible (i.e. within 4 days) it was stored at -80 °C. Soil chemical analysis was conducted by the Environmental Chemistry Laboratory, Landcare Research, Palmerston North, using standard methods [49–51]. From each subplot soil sample, a subsample of 200 g of soil was analyzed for electrical conductivity (EC), water content (Water.Content), organic carbon (Organic.C), Olsen-phosphorus (Olsen.P), total nitrogen (Total.N), NO_3_- (NO3.N), NH_4_+ (NH4.N), and pH.

#### Data loggers

Data loggers (Onset HOBO ^*TM*^ Pro v2 U23-002, Onset Computer Corporation, 470 MacArthur Blvd, Bourne, MA 02532, US) for temperature and humidity were placed at the soil surface, and at a depth of 100 mm below the soil surface and 1.2 m above the soil surface attached to the south side of a tree, in a randomly allocated location in each plot. These data were collected every 30 min (with some gaps) over a 2 year period from December 2010.

### Environmental data preparation

Data for 15 chemical and physical variables were collected (Figure SA17 in Additional file 2). Most soil chemistry values were log transformed to adjust skewed distributions. A number of variables were highly collinear (Figure SA17 in Additional file 2), most notably temperature/elevation, and electrical conductivity/organic carbon/total nitrogen/NH_4_/soil water content, and aspect/cos.aspect/sin.aspect. The number of variables included in the analysis models was reduced by excluding those with high VIF, which provide an index of the severity of multicollinearity. VIF was calculated for all of the variables, after which the variable with the highest VIF ≥10 was excluded, followed by recalculation of VIF for the remaining variables. This process was repeated in a stepwise manner until the VIF for each remaining variable was <10 [52]. This resulted in the exclusion of six variables (elevation, aspect, soil water content, electrical conductivity, organic carbon, and total nitrogen), but see [53] for a caution regarding this approach.

### DNA extraction, PCR, and Sanger sequencing of invertebrates

Genomic DNA from invertebrates collected in pitfall traps or leaf litter collections was extracted nondestructively. Individual specimens were soaked in 420 *μ*l of Tissue Lysis Buffer DXT and 4.2 *μ*l of DXT enzyme mix overnight at 56 °C and the solution was then used to extract DNA on the QIAxtractor®;system using the protocol described by the manufacturer (Qiagen, Hilden, Germany). Individual invertebrates were dried and returned to storage. The cytochrome c oxidase subunit I (COI) from the mitochondrial genome was amplified using the LCO1490 (5’-GGTCAACAAATCATAAAGATATTGG-3’) and HCO2198 (5’-TAAACTTCAGGGTGACCAAAAAATCA-3’) primer pair [54]. PCR amplifications were performed in 50 *μ*l volumes containing the following: 1x PCR buffer (20 mM Tris-HCl (pH 8.4), 50 mM KCl); 2.5 mM MgCl2; 200 mM dNTPs; 1.5 U Platinum Taq (Invitrogen) and 10 pM of each primer. In most cases, 5 *μ*l of each template DNA was added to each reaction. Amplification was carried out with a thermocycling profile of an initial 5 min at 94 °C, followed by 30 cycles of 30 s at 94 °C, 30 s at 48 °C, 1 min at 72 °C, and ending with a final extension time of 10 min at 72 °C. PCR products were purified using 0.15 U Shrimp Alkaline Phosphatase, 0.15 U DNA Exonuclease I and 0.3 *μ*l PCR buffer per 5 *μ*l of PCR product heated to 37 °C for 1 h, followed by deactivation at 85 °C for 15 min. Purified PCR products were sequenced by Macrogen Korea (Geumchen-gu, Seoul, Korea) using an ABI3730XL (Applied Biosystems Inc., Foster City, California). Bidirectional sequencing of the PCR products was conducted with each primer pair. The resulting sequences were aligned and checked for errors using Geneious Pro v5.5 (Biomatters, Auckland, New Zealand) [55] and exported as consensus sequences in FASTA format.

### eDNA extraction, PCR, and pyrosequencing from soil

DNA was extracted from 1.5 g of soil using the MoBio RNA Powersoil kit with the accessory DNA elution kit according to the manufacturer’s instructions (MoBio Laboratories, Carlsbad, California). This approach captures DNA both from organisms living in the soil (intracellular) and from the soil matrix (extracellular; however, most extracellular DNA will be degraded into short fragments and will therefore under-represented in the downstream amplification step). In addition, the samples were spun through a Qiagen DNA spin column (QIAgen, Hilden, Germany) and then a OneStep ^*TM*^ PCR Inhibitor Removal Kit (Zymo Research, California, USA) to remove humic contaminants. To allow amplification of a wide range of target sequences we used a two step amplification protocol. The first rounds of PCR used universal bacterial 16S primers 530F (GTGCCAGCMGCNGCGG) and 1100R (GGGTTNCGNTCGTTG) [56], metazoan-targeted 18S primers #3 (GYGGTGCATGGCCGTTSKTRGTT) and #5_RC (GTGTGYACAAAGGBCAGGGAC) [57], fungal ITS-1 primers ITSF (CTTGGTCATTTAGAGGAAGTAA) and ITSR (GCTGCGTTCTTCATCGATGC) [58], plant *trn*L (UAA) intron primers *c* (CGAAATCGGTAGACGCTACG) and *d* (GGGGATAGAGGGACTTGAAC) [44], or mitochondrial COI primers LCO1490 and HCO2198 [54]. Forward and reverse primers were, respectively, modified with M13 forward (TGTAAAACGACGGCCAGT) and reverse tags (CAGGAAACAGCTATGACC) on their 5’ ends. A second round of PCR was used to add M13 modified Roche MID tags with 454 LibA (CGTATCGCCTCCCTCGCGCCATCAG) and LibB (CTATGCGCCTTGCCAGCCCGCTCAG) adapter sequences.

PCRs were conducted in a 25 *μ*l volume and contained 5–50 ng DNA for the first round, or 1 *μ*l of 1:50 or 1:100 dilution of the first round amplification products for the second round as a template. The reactions also contained 1X Buffer, 2.25 mM Mg, 0.2 mM of each primer, 0.2 mM dNTPs, 0.5 U KAPA2G Robust polymerase (Kapa Biosystems Inc, Boston, Massachusetts). The first round amplification conditions were 95 °C for 3 min followed by 25 (16S), 27 (18S), or 30 (ITS-1, *trn*L, COI) cycles of 95 °C for 30 s, 48 °C (COI), 49 °C (ITS-1) 51 °C (*trn*L), 58 °C (18S), or 60 °C (16S) for 30 s, 72 °C for 45 s, with a final extension of 72 °C for 5 mins. For second round PCRs, to add the MID tags, 12 amplification cycles with an annealing temperature of 60 °C were used for all of the samples. Second round amplifications were conducted in five separate reactions, which were subsequently pooled, cleaned up using the AMPure XP magnetic bead method (Beckman Coulter, MA, USA), and quantified using the Qubit dsDNA HS Assay Kit (Life Technologies, New York, USA). Cleaned up pools from each sample were combined in equal proportions for each amplicon, after which 125 ng of each amplicon was pooled for a total of 5 *μ*g to be sequenced using a Lib-A Titanium protocol (Roche, Switzerland) on the 454 GS-FLX system at Macrogen (Geumchen-gu, Seoul, Korea).

### Invertebrate isolation from soil, eDNA extraction, PCR, and pyrosequencing

Soil invertebrates were extracted from approximately 50 cc of soil using a modified sugar centrifugation method developed by Freckman & Virginia [59]. A modified version of this method has proven to be very robust in extracting soil invertebrates from a variety of soil types with little damage to the specimens [60]. Approximately 650 ml of clean tap water was combined with soil in a glass beaker and stirred in a figure of eight for 30 s, and was then immediately poured onto a wetted 40 mesh (425 *μ*m) screen which was stacked on top of a 400 mesh (38 *μ*m) screen. Screens were then gently rinsed, at an angle, with cold tap water, washing soil invertebrates through the top of the stacked screens. The top screen was then removed and examined under a dissecting microscope (6-50X magnification) for the presence of soil invertebrates that were too large to fit through the 40 mesh (425 *μ*m) screen. The soil invertebrates and remaining soil were then gently backwashed into 50 ml plastic centrifuge tubes. The samples were then centrifuged at 1,750 RPM in an Eppendorf 5810 centrifuge for 5 min to form a small pellet. Following initial centrifugation, all but a few ml of liquid were carefully decanted off the pellet and replaced with an equal amount of 1.33 M chilled sugar solution (454 g white table sugar/L water). The pellet was then gently stirred to break it up, re-suspended, and then returned to the centrifuge for one minute at 1,750 RPM. The sugar solution, containing suspended soil invertebrates, was then decanted onto a wet 500 mesh (25 *μ*m) screen, and was then gently rinsed with tap water and backwashed with approximately 10 ml of water into a clean centrifuge tube. Samples were fixed in 90 % ethanol to allow for molecular analysis.

Ethanol was removed by drying the samples in a heat block at 50 °C. The DNA was extracted from each of the 20 samples using a QIAxtractor (Qiagen, USA). The DNA extractions were amplified using forward 454 PCR fusion primers that contain the 454 emulsion PCR adapter, joined to a 10-base-pair multiplex identifier (MIDs) with the LCO1490 and reverse 454 PCR fusion primers that contain the 454 emulsion PCR adapter and HCO2198. A PCR was performed using a Veriti thermal cycler (Life Technologies). The PCR conditions were 3 min at 94 °C, followed by 35 cycles of 30 s at 94 °C, 30 s at 48 °C, 45 s at 72 °C, and finally 5 min at 72 °C. For each sample, 25 *μ*l reactions were carried out using a Roche FastStart High Fidelity PCR system (Roche, USA) with 1 *μ*l BSA (10 g/l) added. PCR products were cleaned with Agencourt AMPure XP magnetic beads (Beckman Coulter) to remove the primer dimers. The purified PCR products were quantified using a Fluorometer (QuantiFluor, Promega, USA), checked using a Bioanalyzer 2100 (Agilent Technologies, USA) for removal of PCR primer dimers, and an equimolar of the samples was pooled in a single tube. This pool was amplified using the Lib-A method and sequenced on a 454 GS Junior system (Roche) at Landcare Research, Auckland.

### OTU identification and bioinformatics pipeline

Geneious [55] was used to deconvolute the standard flowgram format files (SFF) encoding raw 454 sequencing results. During the deconvolution, the site information (e.g. plot and subplot name) was added into the sequence labels for later analysis. The raw reads file in a FASTQ format was then passed into a UPARSE [35] pipeline to identify OTUs. This pipeline includes quality filtering, length truncation (300 bp), dereplication, abundance sorting, OTU clustering, and chimera filtering. Before the dereplication step, all of the reads were processed by Acacia [34] for error correction. The output of the pipeline was a FASTA file containing OTU sequences, and a mapping file between OTUs and reads for each given OTU clustering threshold. A community matrix was then created from the mapping file for each locus by retrieving the sample information in each sequence label and cross referencing with OTU identity of the read. The resulting community matrix has a row for each sample and a column for each OTU, and is populated by abundances as measured by OTU read counts per sample. Additionally, an alternative set of community matrices were constructed for each amplicon dataset in which all single-read OTUs were removed (see Additional file 1 for a full set of parallel analyses).

Jost’s biodiversities [61] were calculated from the community abundance matrices of six eDNA methods using the R package *vegetarian* [37]. Rarefaction curves for diversities were estimated based on the 97 % sequence similarity threshold for OTU identification in the R ecology package *vegan* [62]. Correlations of diversity and environmental factors, and correlations of eDNA methods and traditional methods were also computed. Finally, BLAST+ was used to classify the taxonomy of OTUs and MEGAN 5 [36] was used to interpret and visualize the BLAST+ results.

### Comparison of eDNA and traditional biodiversity measures

Traditional and eDNA methods were compared in three ways: (1) in their ability to detect community differences associated with elevation, (2) in a pairwise community correlation analysis of between-plot beta diversities, and (3) by measuring the similarity of methods via the correlation of their plot priorities. Pairwise community correlations were used to form a matrix describing the correlations among pairwise beta diversity from traditional and eDNA community samples using a Mantel test for significance. This matrix of correlations was then used as a similarity matrix to produce a second-stage MDS plot to determine which pairs of communities vary in composition across the plots in the most correlated manner (Fig. 10).

The plots were also ranked by their conservation priority as measured by each biodiversity measure in turn. Conservation priority rankings were determined by iteratively removing the next plot that maximized the beta-1 diversity of the remaining plots. A Spearman’s correlation coefficient was then computed for each pair of biodiversity measures to determine which measures produced the most similar priority ranking. Of particular interest was which eDNA markers produced the most similar priority rankings to traditional measures.

### Multivariate ordination of samples and environmental data

The differences between the communities detected in samples were visualized using non-metric multidimensional scaling plots generated using *vegan* and *ggplot2*. Constrained ordination of community data with environmental data as constraining variables was carried out using the capscale function, which is a non-Euclidean generalization of redundancy analysis, from the R package *vegan* [62]. Three ordination scenarios were tested with each eDNA dataset and also with the traditional vegetation datasets: (i) models were constructed containing each of the fifteen environmental variables in isolation; (ii) a model was constructed using the combined set of variables with VIF <10; and, (iii) more conservative models were constructed by using subsets of the variables with VIF <10 chosen by stepwise forward and backward selection model building procedures. The community data ordinations were based on the Jaccard distance measure and the significance of variables included in each model was determined by permutation tests.

To visualize the similarities in sample ordination based on eDNA and traditional community biodiversity measures Procrustes plots of the five main eDNA datasets (16S, 18S, trnL, ITS, COI) and the three main traditional measures (seedlings, trees, invertebrates) where produced, and PROTEST from the R package *vegan* [62] was used to measure the significance of the correlations in ordination between different methods.

## Availability of supporting data

All of the sequence data produced by this project are freely available. Environmental DNA sequences have been deposited in the NCBI Sequence Read Archive (Project Accession: PRJNA267737). Sanger sequences of invertebrates have been deposited in GenBank with their New Zealand Arthropod Collection codes (GenBank accession numbers KP420745- KP422464). The 12 community matrices of eDNA and traditional methods in the CSV format, OTU representative sequences at 97 % clustering threshold, BLAST output, images of invertebrates, soil chemistry, elevation, and temperature data have been deposited in GigaDB [33]. All of the R scripts used to analyse data and produce figures are available at [63]. Attribution should be made by citation of this paper.

## Additional files

Additional file 1 **Supplementary information B.** Evaluating a multigene environmental DNA approach for biodiversity assessment. (PDF 1075 kb)

Additional file 2 **Supplementary information A.** Evaluating a multigene environmental DNA approach for biodiversity assessment. (PDF 2089 kb)

## Abbreviations

eDNA: Environmental DNA
NGS: Next generation sequencing
OTU: Operational taxonomic unit
VIF: Variance inflation factor
