# Supplementary material for: Evaluating a multigene environmental DNA approach for biodiversity assessment
Source: Gigascience. 2015 Oct 6;4:46. doi: 10.1186/s13742-015-0086-1 (PMC4595072; doi:10.1186/s13742-015-0086-1)
Supplement: Additional file 1 — Supplementary information B. Evaluating a multigene environmental DNA approach for biodiversity assessment. (PDF 1075 kb) [file 13742_2015_86_MOESM1_ESM.pdf]

SUPPLEMENTARY INFORMATION B

Supplementary Information B: Evaluating a multigene environmental DNA approach for biodiversity assessment

Alexei J Drummond[1,2*], Richard D Newcomb[1,3,4], Thomas R Buckley[1,3,5], Dong Xie[1,2], Andrew Dopheide[1,3,4], Benjamin CM Potter[1,3], Joseph Heled[1,2], Howard A Ross[1,3], Leah Tooman[1,4], Stefanie Grosser[1,5], Duckchul Park[5], Nicholas J Demetras[9], Mark I Stevens[6,7], James C Russell[1,3,7], Sandra H Anderson[3], Anna Carter[1,10] and Nicola Nelson[1,10]

*Correspondence:
alexei@cs.auckland.ac.nz
[1]Allan Wilson Centre, University of Auckland, Auckland, New Zealand
Full list of author information is available at the end of the article

Result of removing all singleton OTUs from the eDNA datasets

The figures and tables below are the result of removing all of the singleton OTUs from the eDNA datasets.

Author details
[1]Allan Wilson Centre, University of Auckland, Auckland, New Zealand. [2]Department of Computer Science, University of Auckland, Private Bag 92019, Auckland 1142, New Zealand. [3]School of Biological Sciences, University of Auckland, Private Bag 92019, Auckland 1142, New Zealand. [4]The Institute for Plant and Food Research, Private Bag 92169, Auckland 1142 , New Zealand. [5]Landcare Research, Private Bag 92170, Auckland 1142 , New Zealand. [6]South Australian Museum, North Terrace, Adelaide SA 5000 , Australia. [7]School of Pharmacy and Medical Sciences, University of South Australia, GPO Box 2471, Adelaide SA 5001 , Australia.
[8]Department of Statistics, University of Auckland,, Private Bag 92019, Auckland 1142 , New Zealand.
[9]Department of Biological Sciences, University of Waikato, Private Bag 3105, Hamilton 3240 , New Zealand.
[10]School of Biological Sciences, Victoria University of Wellington, PO Box 600, Wellington 6140 , New Zealand.

References

Figure SB1 The number of OTUs at the 97% clustering threshold across the number of sites.

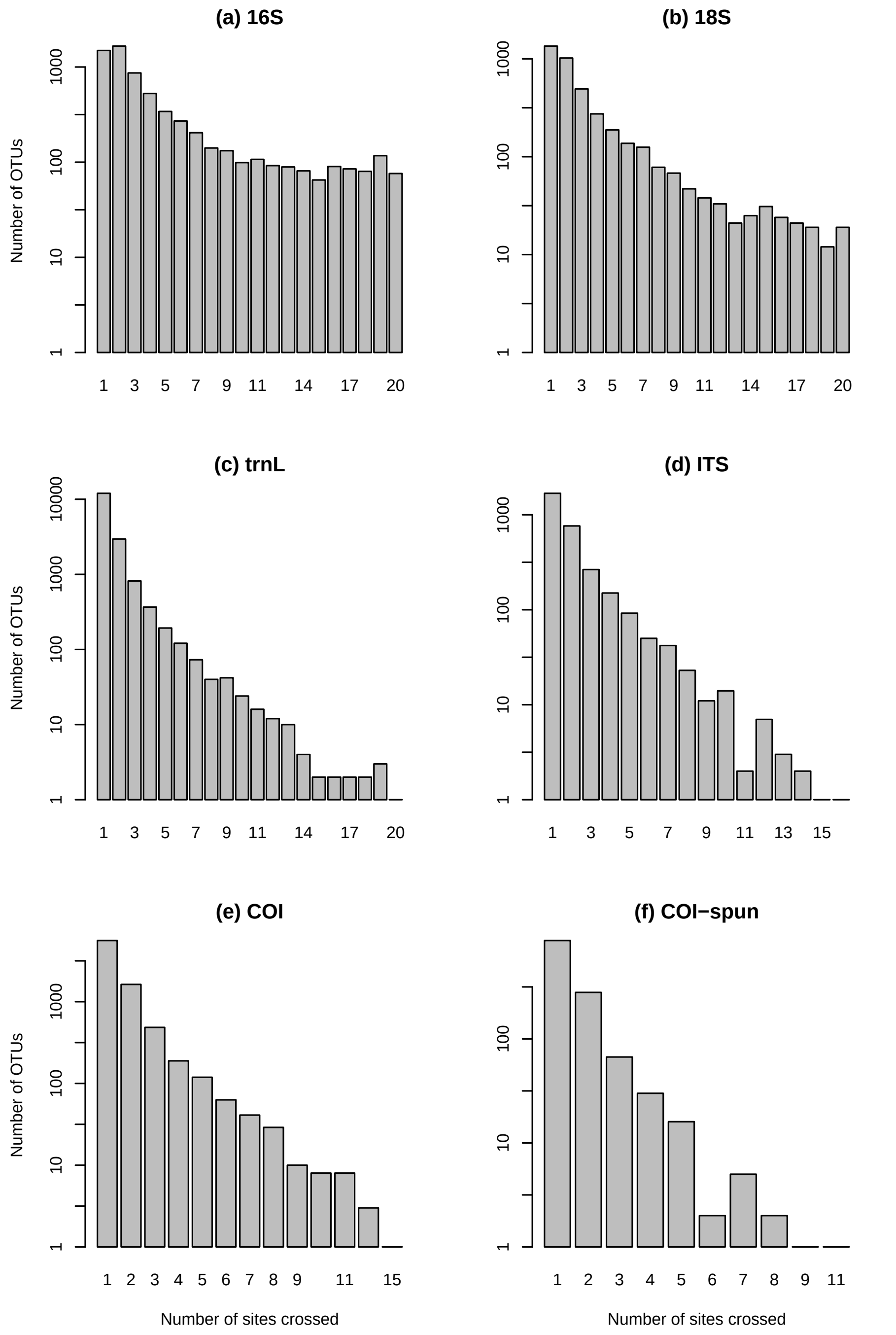

Figure SB2 The number of OTUs at the 97% clustering threshold assigned to phyla. Unclassified OTUs and OTUs containing low-complexity sequences are not included, OTUs from phyla that are represented by less than 0.1% of the OTUs are grouped into the ‘Others’ category

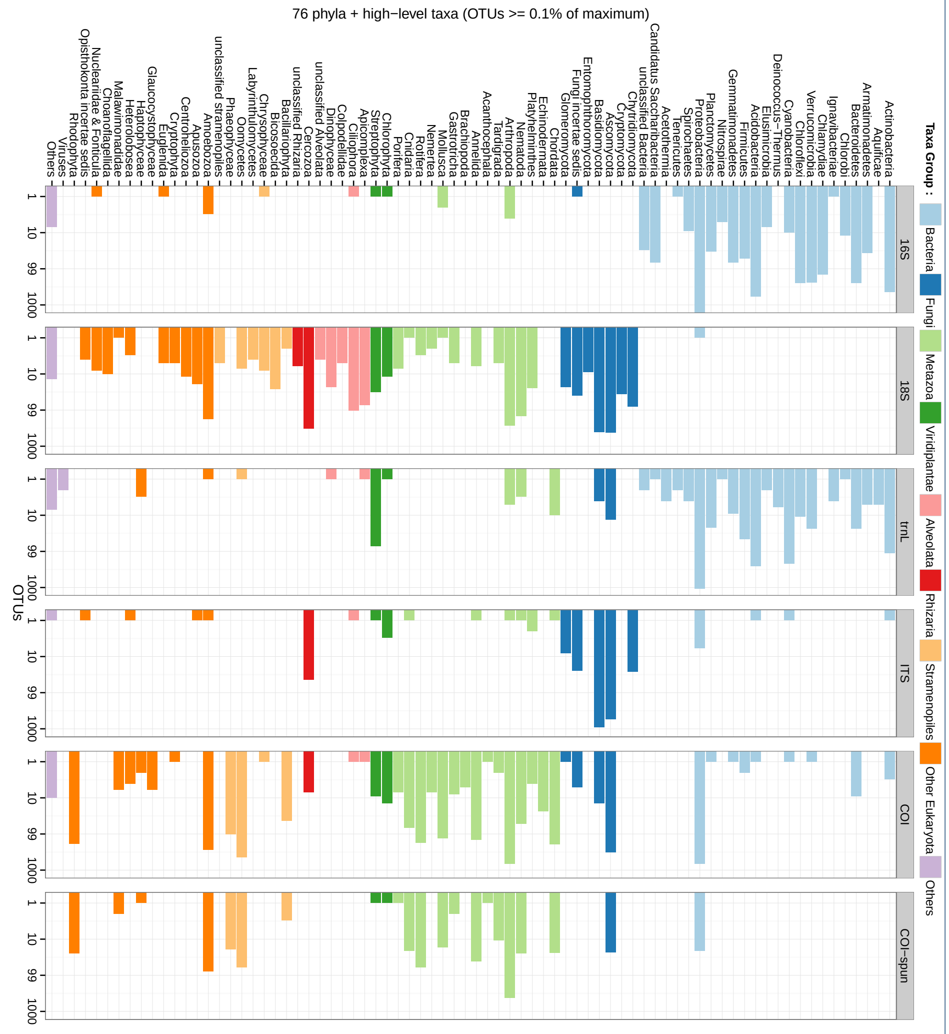

Figure SB3 Plots of diversities using cutoff thresholds ranging from 90-100% for OTU classification of: (a) α diversity, (b) effective α diversity, (c) β diversity, (d) effective β diversity, (e) γ diversity, and (f) effective γ diversity. Molecular datasets include 16S, 18S, *trn*L, ITS, COI, and COI-spun.

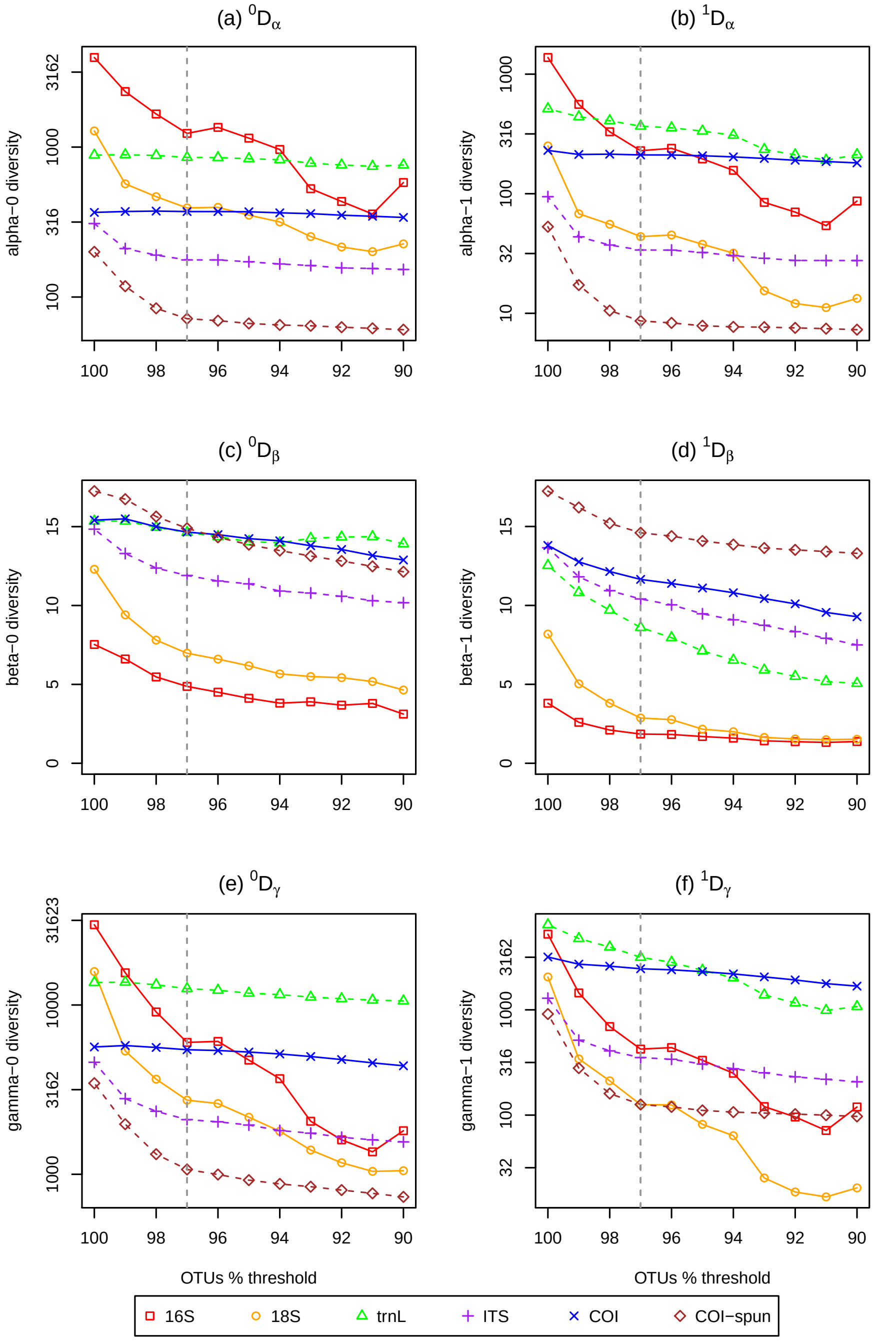

Figure SB4 Rarefaction curves for diversities estimated using a 97% threshold for OTU classification of: (a) α diversity, (b) effective α diversity, (c) β diversity, (d) effective β diversity, (e) γ diversity, and (f) effective γ diversity. Molecular datasets include 16S, 18S, *trn*L, ITS, COI, and COI-spun.

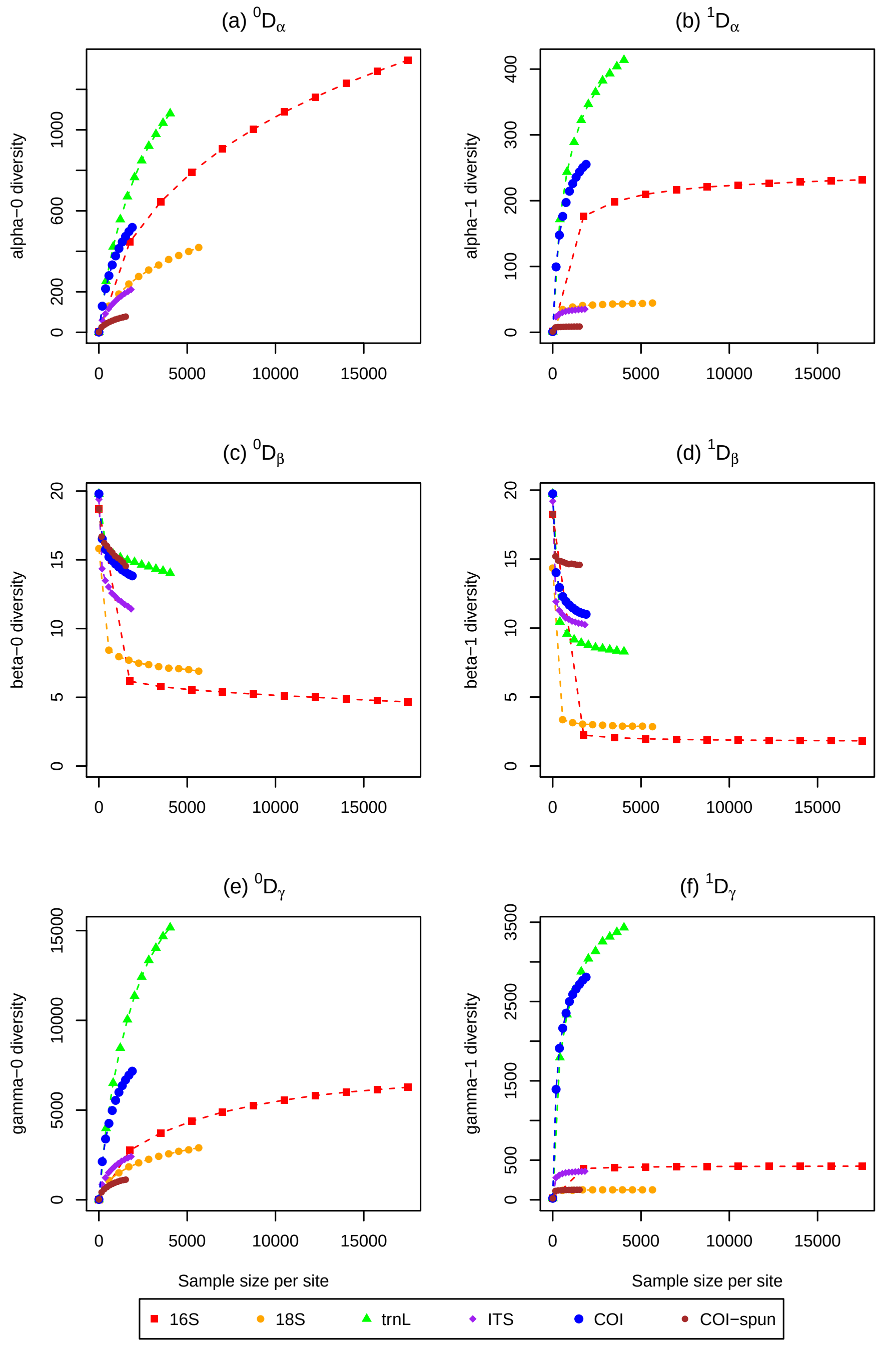

Figure SB5 Box and whisker plots of turnover (normalized pairwise effective β diversity) within (red) and between plots (blue) for the molecular methods 16S, 18S, *trn*L, ITS, COI, and COI-spun.

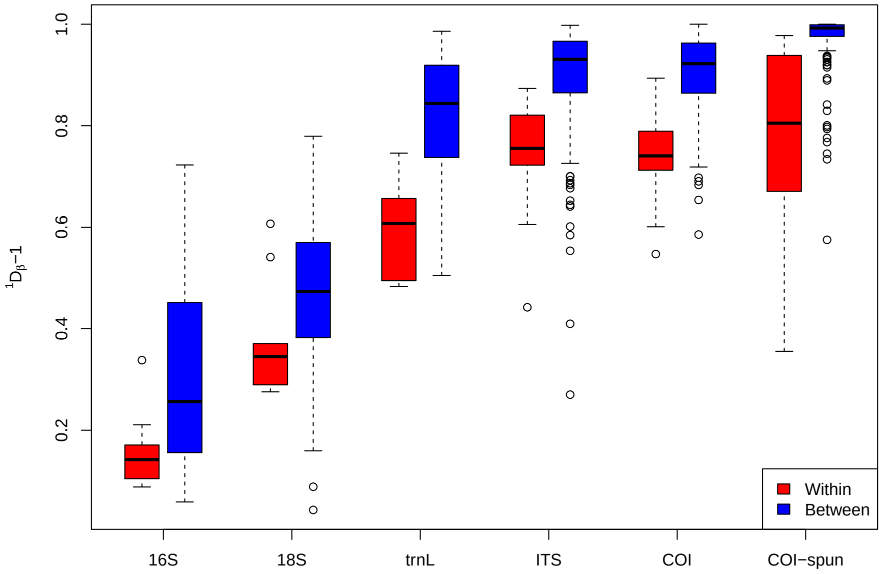

Figure SB6 Regression of effective β diversity and difference in elevation for: (a) the eDNA biodiversity datasets (16S, 18S, *trn*L, ITS, COI-soil and COI-soil spun) and (b) traditional biodiversity datasets (seedlings, trees, invertebrates, birds).

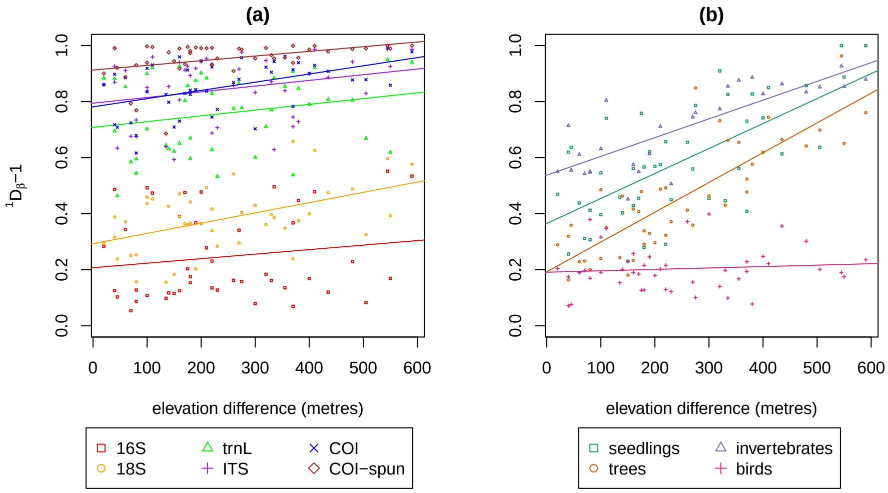

Figure SB7 Regression of effective α diversity as a function of elevation for: (a) the eDNA biodiversity datasets (16S, 18S, *trn*L, ITS, COI-soil and COI-soil spun) and (b) traditional biodiversity datasets (seedlings, trees, invertebrates, birds).

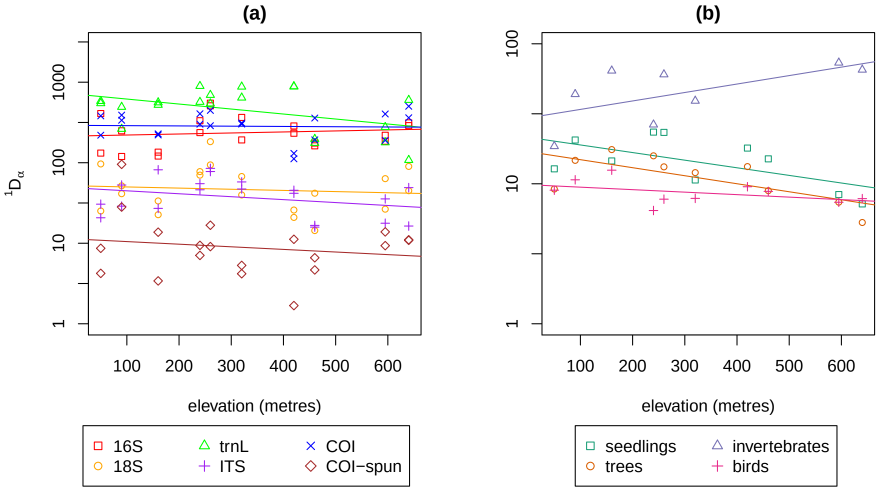

Figure SB8 Non-metric multidimensional scaling of effective β diversity for paired subplots for the molecular datasets 16S, 18S, *trn*L, ITS, COI-soil and COI-soil spun.

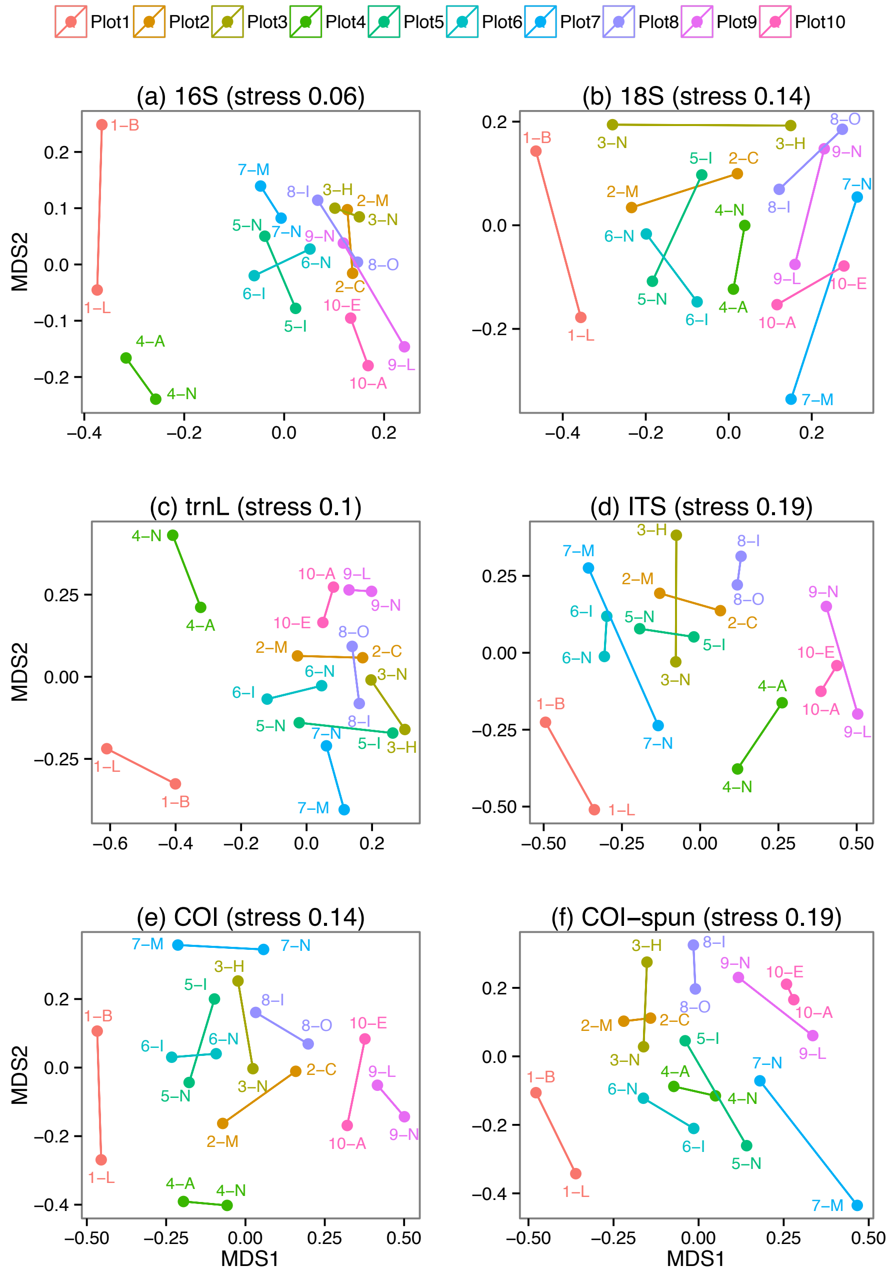

Figure SB9 Procrustes analysis of effective β diversity between the eDNA datasets and traditional datasets, their significance level in parentheses is estimated based on 4,999 permutations.

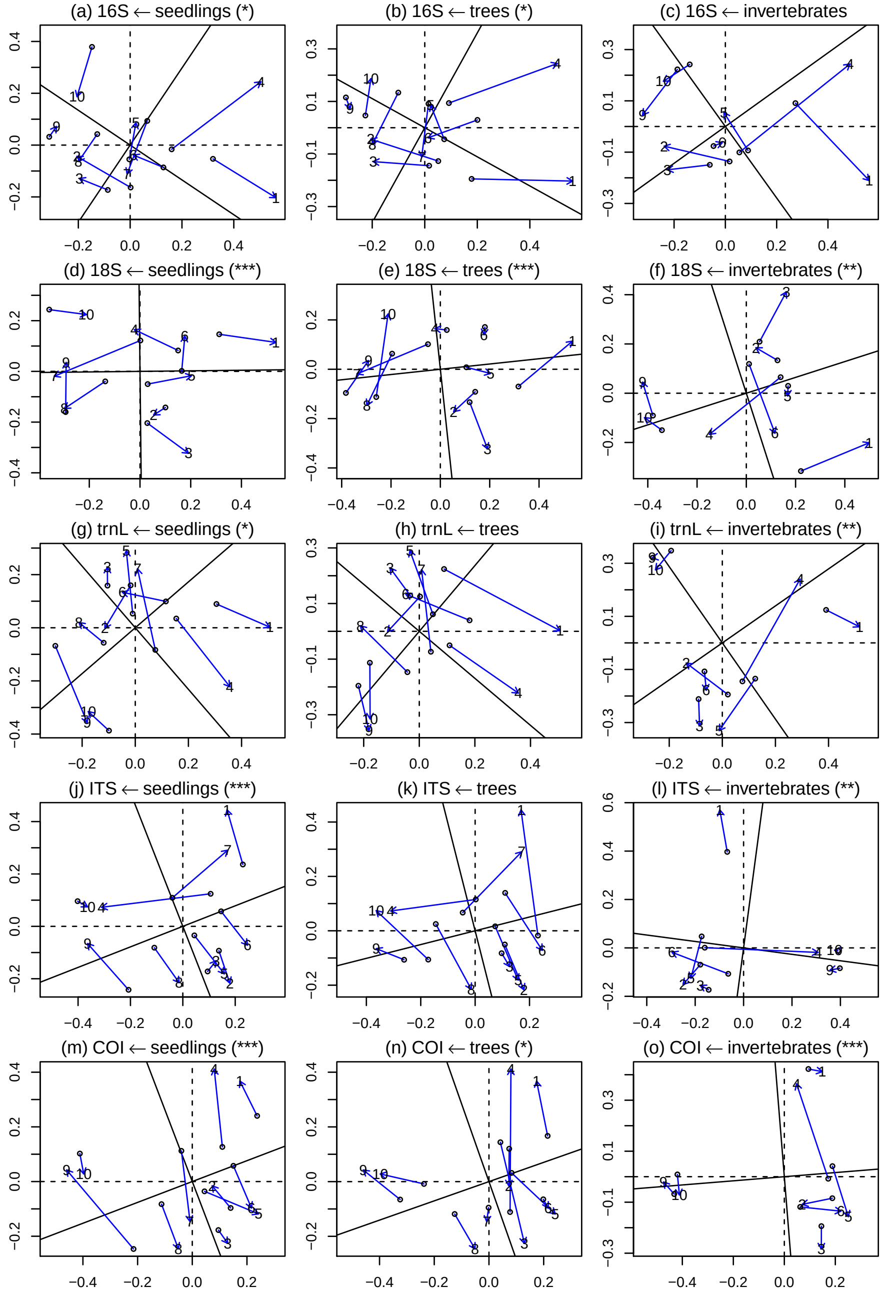

Figure SB10 Multidimensional scaling (no birds) of pairwise community matrix correlations of effective β diversity within and between the eDNA datasets (16S, 18S, trnL, ITS, COI-soil and COI-soil spun) and traditional datasets (seedlings, trees, invertebrates).

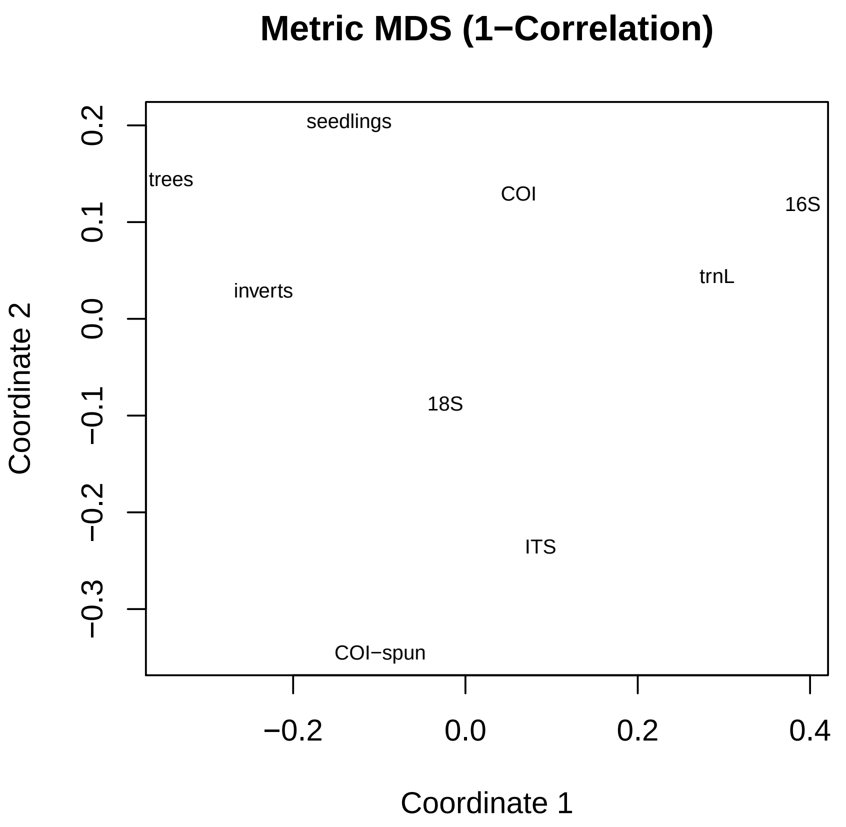

Figure SB11 The probability of having a) maximum γ diversity and b) maximum effective β diversity of all possible combinations of the four plots for the eDNA biodiversity datasets (16S, 18S, *trn*L, ITS, COI-soil and COI-soil spun) and the traditional biodiversity datasets (seedlings, trees, invertebrates, birds).

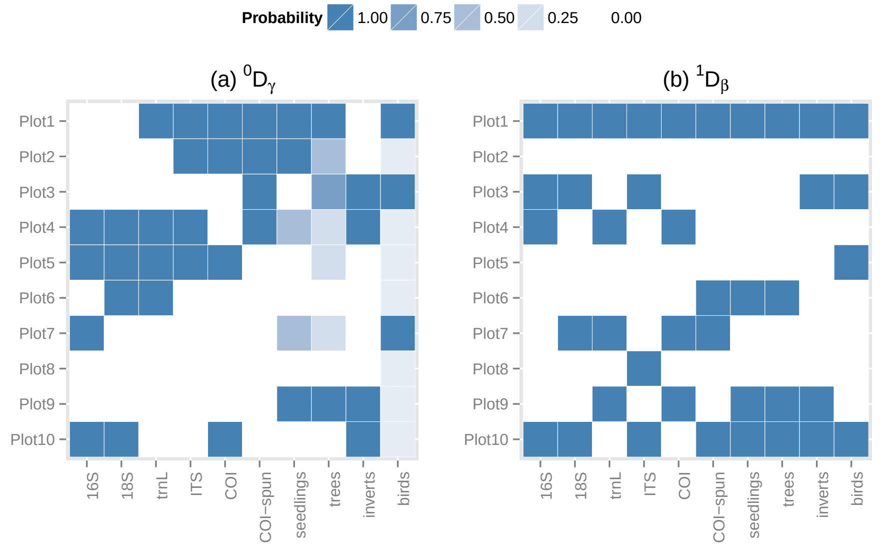

Figure SB12 Maximum remained γ diversity as a function of number of sites for (a) eDNA biodiversity datasets (16S, 18S, *trn*L, ITS, COI-soil and COI-soil spun) and (b) traditional biodiversity datasets (seedlings, trees, invertebrates, birds). Plots were removed sequentially so as to minimize the loss of overall γ diversity among the remaining plots. This procedure was undertaken independently for each of the ten biodiversity measures.

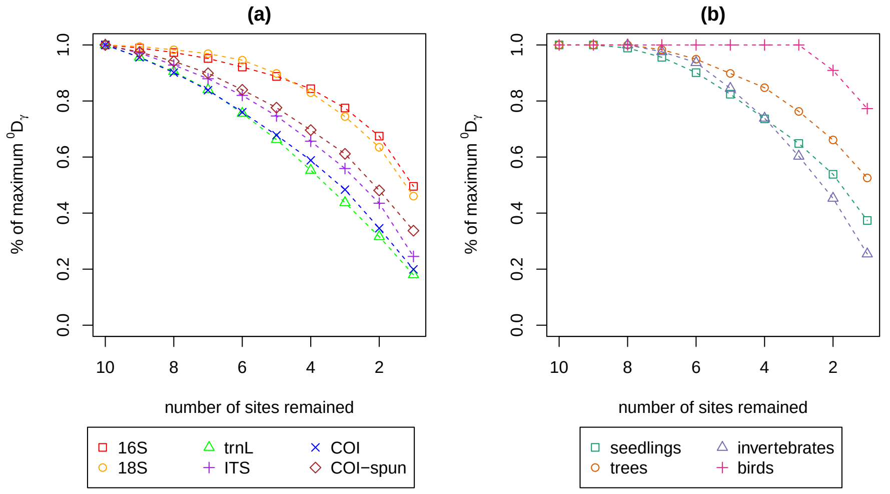

Figure SB13 Maximum remained effective β diversity as a function of the number of sites for (a) eDNA biodiversity datasets (16S, 18S, *trn*L, ITS, COI-soil and COI-soil spun) and (b) traditional biodiversity datasets (seedlings, trees, invertebrates, birds). Plots were removed sequentially so as to maximize the resulting effective β diversity among the remaining plots. This procedure was undertaken independently for each of the ten biodiversity measures.

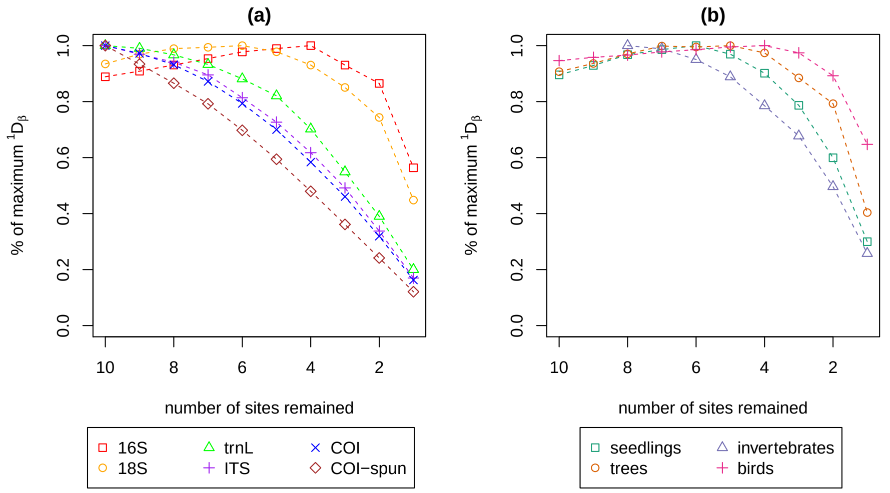

Figure SB14 Models constructed by the combined set of variables with VIF < 10 using distance-based redundancy analysis for the molecular datasets 16S, 18S, *trn*L, ITS, COI, and COI-spun.

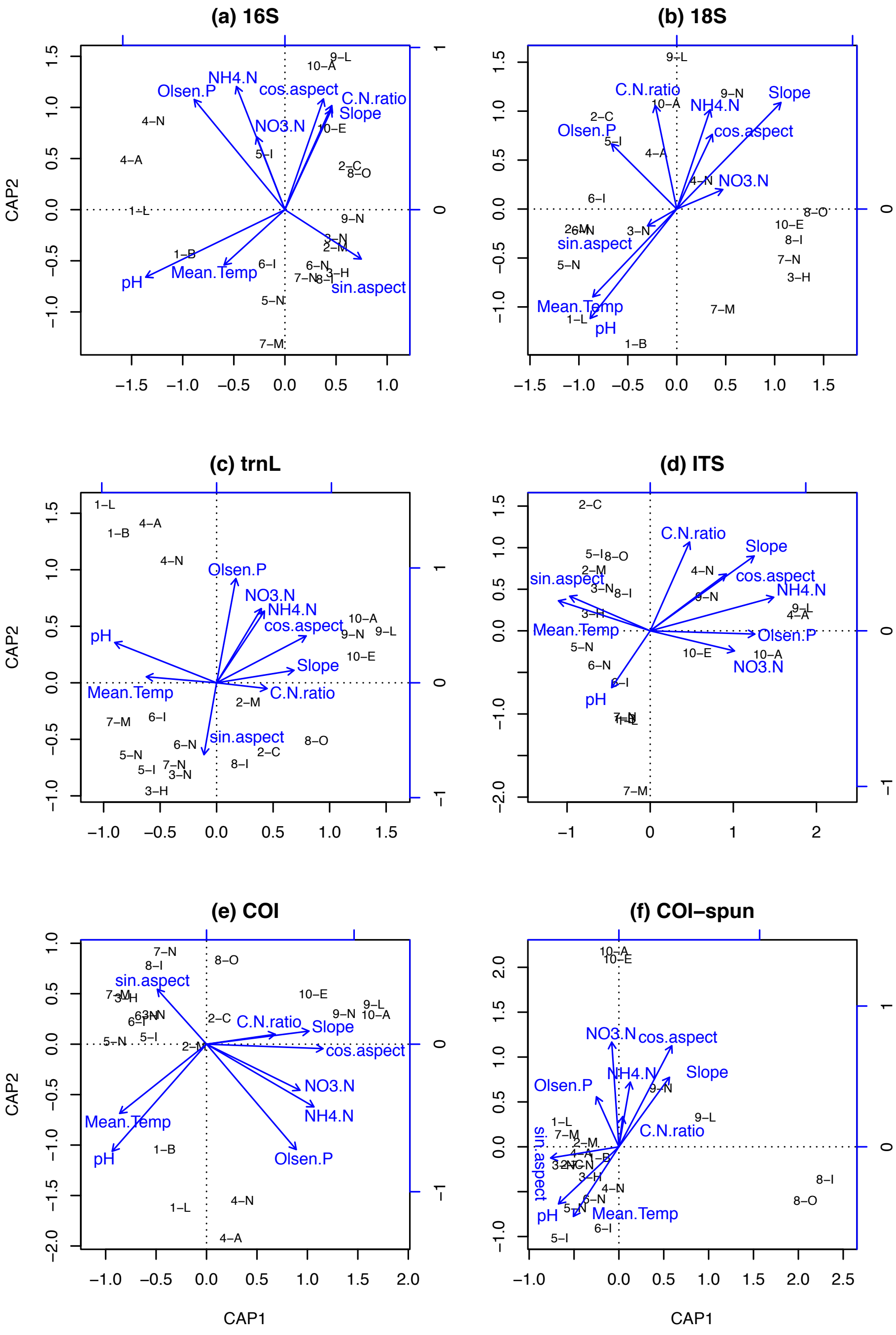

Figure SB15 More conservative models constructed by the subsets of the variables with VIF < 10 chosen by stepwise forward selection model building procedures using distance-based redundancy analysis for the molecular datasets 16S, 18S, *trn*L, ITS, COI, and COI-spun.

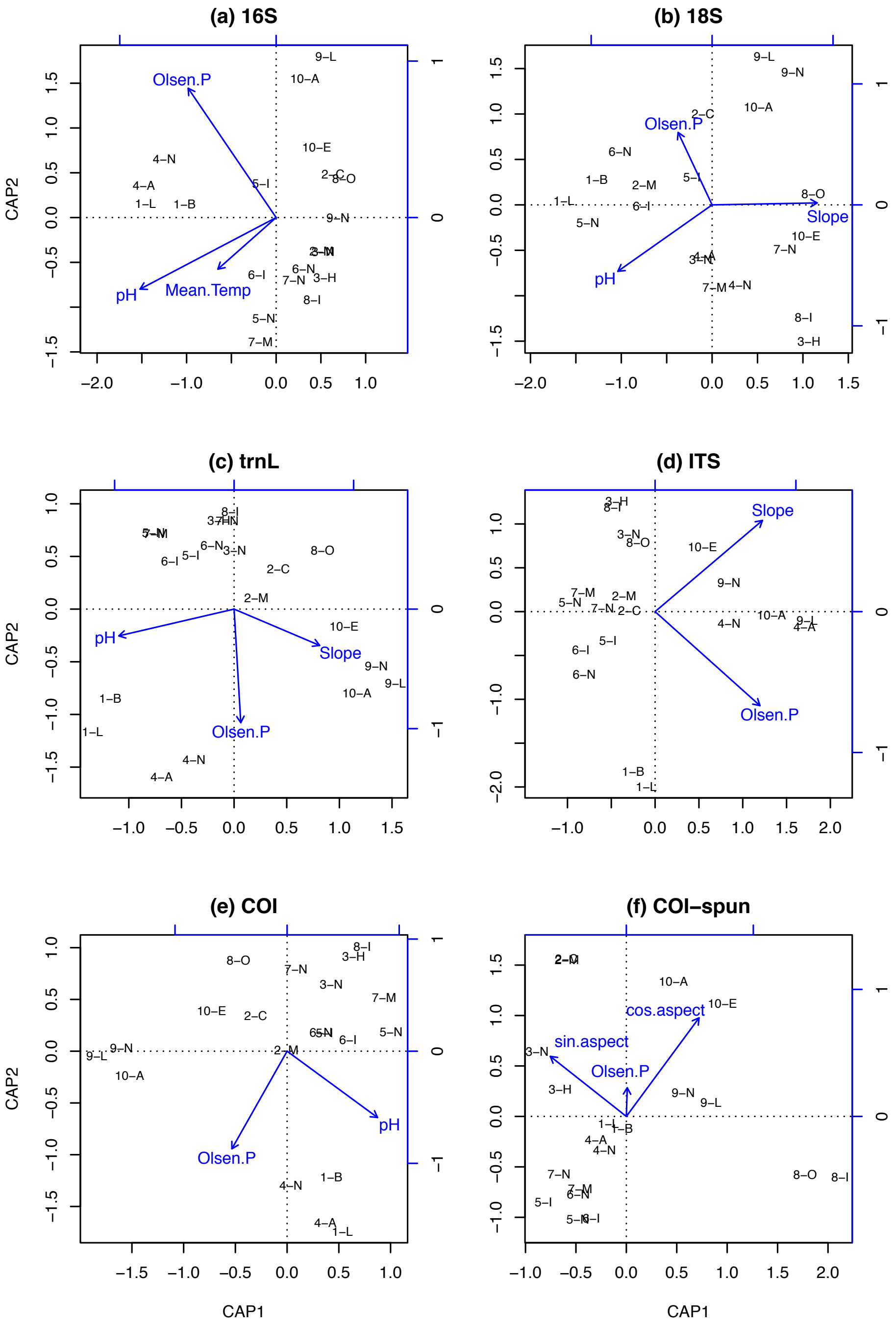

Figure SB16 More conservative models constructed by the subsets of the variables with VIF < 10 chosen by stepwise backward selection model building procedures using distance-based redundancy analysis for the molecular datasets 16S, 18S, *trn*L, ITS, COI, and COI-spun.

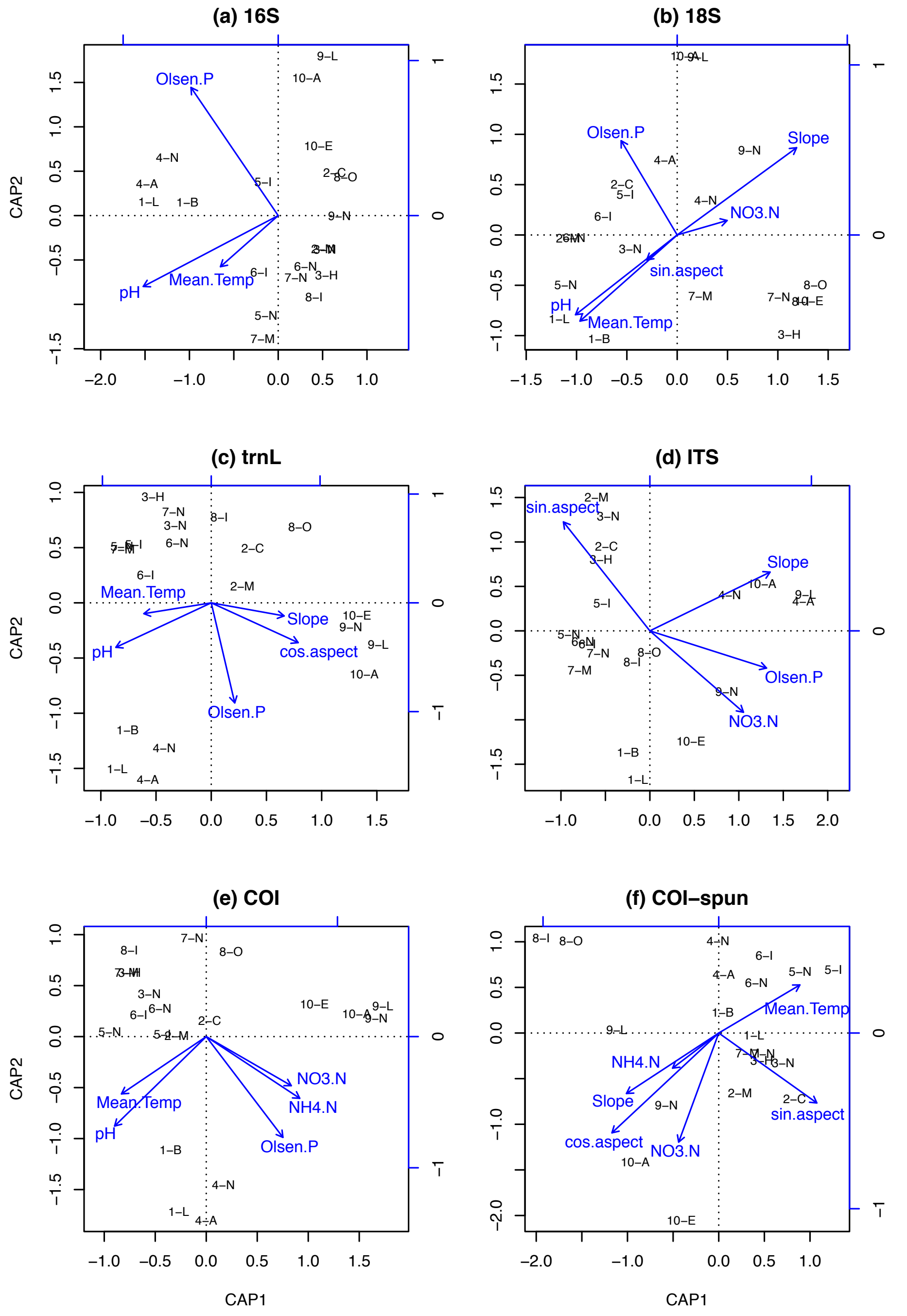

Table SB1 Table of sequences per plot for 16S, 18S, *trn*L, ITS, COI, and COI-spun molecular datasets.

	16S	18S	trnL	ITS	COI	COI-spun	Total
Plot1	63,878	27,317	12,236	9,794	9,522	11,102	133,849
Plot2	46,891	100,338	17,756	19,355	7,689	7,886	199,915
Plot3	47,637	34,089	9,926	12,430	6,584	7,820	118,486
Plot4	59,431	50,368	13,264	14,330	6,722	6,104	150,219
Plot5	92,724	86,554	13,802	17,783	9,835	4,448	225,146
Plot6	37,248	78,034	13,705	12,093	8,584	3,608	153,272
Plot7	52,608	15,016	10,868	3,824	5,637	5,316	93,269
Plot8	54,367	17,812	20,655	23,229	7,731	5,766	129,560
Plot9	50,374	73,877	12,046	11,630	7,560	5,021	160,508
Plot10	50,400	35,002	19,911	4,569	7,827	5,028	122,737

Table SB2 Table of OTUs per plot for 16S, 18S, *trn*L, ITS, COI, and COI-spun molecular datasets.

	16S	18S	trnL	ITS	COI	COI-spun	Total*
Plot1	2,012	1,031	2,286	451	1,314	143	7,237
Plot2	1,946	1,125	2,504	631	1,376	434	8,016
Plot3	1,812	839	2,335	611	1,137	272	7,006
Plot4	3,133	1,560	2,286	763	1,137	223	9,102
Plot5	3,276	1,852	2,956	734	1,632	112	10,562
Plot6	2,150	1,498	3,003	585	1,119	96	8,451
Plot7	2,123	505	2,594	444	709	86	6,461
Plot8	1,953	640	1,859	533	1,189	149	6,323
Plot9	1,989	980	1,379	359	877	123	5,707
Plot10	2,264	1,088	1,875	391	1,320	119	7,057

Table SB3 Table of Shannon index per plot for 16S, 18S, *trn*L, ITS, COI, and COI-spun molecular datasets.

	16S	18S	trnL	ITS	COI	COI-spun
Plot1	255.61	99.52	898.09	43.59	522.09	10.95
Plot2	192.68	57.34	569.27	61.94	656.28	91.95
Plot3	142.04	42.20	853.05	90.06	361.07	13.54
Plot4	571.37	184.98	1,019.56	141.93	589.35	22.96
Plot5	345.30	100.95	1,222.87	86.08	666.75	13.66
Plot6	309.60	70.70	1,207.66	84.12	523.90	8.94
Plot7	286.85	32.09	1,412.53	76.14	186.53	5.51
Plot8	189.31	32.96	276.45	19.87	472.69	6.98
Plot9	245.25	56.49	316.49	46.78	434.71	18.78
Plot10	342.21	96.52	328.61	46.62	697.62	17.17

* the total number of OTUs is just each number for each dataset added together.

Table SB4 Mantel statistic r and its significance using Mantel's test based on 4,999 permutations, and R^2 and $p\text{-}value$ for a linear model of the regression of effective β diversity and difference in elevation in Figure SB6.

	Mantel statistic r	significance	R^2	$p\text{-}value$
16S	0.158	0.1864	0.0249	0.301
18S	0.481	0.0038	0.231	0.000823
trnL	0.231	0.102	0.0535	0.126
ITS	0.278	0.0482	0.0771	0.0648
COI	0.489	0.0036	0.239	0.000658
COI-spun	0.399	0.0026	0.159	0.00670
seedlings	0.672	4e-04	0.451	4.37e-07
trees	0.827	2e-04	0.684	2.51e-12
invertebrates	0.813	0.0026	0.661	1.48e-07
birds	0.096	0.3124	0.00929	0.529

Table SB5 The significance test of effective α diversity as a function of elevation in Figure SB7.

	R^2	*p-value*
16S	0.0159	0.596
18S	0.0105	0.667
trnL	0.201	0.0476
ITS	0.0915	0.195
COI	0.00139	0.876
COI-spun	0.0304	0.463
seedlings	0.379	0.0582
trees	0.501	0.0219
invertebrates	0.386	0.100
birds	0.185	0.215

Table SB6 Pairwise community matrix Procrustes analysis of effective β diversity within and between the eDNA datasets and traditional datasets, estimated as a sum of squared differences and their significance in parentheses is based on 4,999 permutations.

	16S	18S	trnL	ITS	COI	COI-spun	seedlings	trees	inverts
18S	0.57 (0.017)								
trnL	0.34 (0.00040)	0.55 (0.016)							
ITS	0.59 (0.017)	0.65 (0.074)	0.34 (0.0012)						
COI	0.44 (0.0024)	0.47 (0.0040)	0.11 (0.00040)	0.47 (0.0070)					
COI-spun	0.82 (0.37)	0.70 (0.11)	0.85 (0.53)	0.78 (0.28)	0.78 (0.27)				
seedlings	0.48 (0.011)	0.41 (0.0012)	0.50 (0.011)	0.47 (0.0038)	0.44 (0.0014)	0.81 (0.38)			
trees	0.63 (0.047)	0.44 (0.0040)	0.69 (0.10)	0.72 (0.13)	0.61 (0.038)	0.53 (0.012)	0.42 (0.0018)		
inverts	0.67 (0.15)	0.41 (0.0090)	0.34 (0.0092)	0.36 (0.0054)	0.26 (0.0044)	0.70 (0.23)	0.28 (0.0014)	0.54 (0.047)	
birds	0.88 (0.54)	0.55 (0.012)	0.87 (0.58)	0.85 (0.48)	0.83 (0.41)	0.66 (0.068)	0.75 (0.20)	0.67 (0.073)	0.63 (0.12)

Table SB7 Pairwise community matrix correlations of effective β diversity within and between the eDNA datasets and traditional datasets, Mantel statistic r, and their significance in parentheses using Mantel's test based on 4,999 permutations.

	16S	18S	trnL	ITS	COI	COI-spun	seedlings	trees	inverts
18S	0.482 (0.0036)								
trnL	0.814 (2e-04)	0.584 (2e-04)							
ITS	0.431 (0.0176)	0.574 (2e-04)	0.614 (4e-04)						
COI	0.635 (2e-04)	0.586 (4e-04)	0.816 (2e-04)	0.607 (6e-04)					
COI-spun	0.341 (0.0338)	0.558 (2e-04)	0.403 (0.0108)	0.604 (6e-04)	0.45 (0.003)				
seedlings	0.501 (0.0502)	0.541 (0.0022)	0.501 (0.0302)	0.482 (0.0026)	0.68 (2e-04)	0.428 (0.0042)			
trees	0.261 (0.1036)	0.552 (0.002)	0.348 (0.0542)	0.402 (0.0074)	0.611 (2e-04)	0.431 (0.0022)	0.816 (2e-04)		
inverts	0.325 (0.1032)	0.694 (8e-04)	0.536 (0.0314)	0.504 (0.0032)	0.802 (4e-04)	0.611 (0.0066)	0.827 (0.0014)	0.827 (0.0044)	
birds	-0.016 (0.414)	0.292 (0.0932)	0.064 (0.3394)	0.125 (0.2278)	-0.145 (0.7008)	0.021 (0.4202)	-0.046 (0.503)	-0.024 (0.4874)	-0.031 (0.501)

Table SB8 Ranking sampling plots by removing plots sequentially so as to minimize the loss of overall γ diversity among the remaining plots from Figure SB12. 1 is the most important and is removed last, 10 is the least important and is removed at the beginning.

Plot name	16S	18S	*trn*L	ITS	COI	COI-spun	seedlings	trees	inverts	birds
Plot1	6	8	4	4	3	4	5	4	8	2
Plot2	8	5	7	3	4	1	6	3	6	10
Plot3	10	7	8	5	8	2	4	7	4	1
Plot4	2	2	3	1	5	3	2	6	2	9
Plot5	1	1	2	2	1	9	1	1	7	8
Plot6	9	4	1	7	6	10	10	10	5	7
Plot7	4	9	5	8	10	8	8	5		3
Plot8	5	10	10	6	7	6	7	9		6
Plot9	7	6	9	10	9	5	3	2	1	5
Plot10	3	3	6	9	2	7	9	8	3	4

Table SB9 Means and standard deviations of Table SB8 between eDNA datasets for ranking plots to minimize the loss of overall γ diversity among the remaining plots.

	eDNA	Traditional	Traditional No Birds	All	All No Birds
Plot1	4.8 ± 1.83	4.8 ± 2.50	5.7 ± 2.08	4.8 ± 1.99	5.1 ± 1.83
Plot2	4.7 ± 2.58	6.2 ± 2.87	5.0 ± 1.73	5.3 ± 2.67	4.8 ± 2.22
Plot3	6.7 ± 2.80	4.0 ± 2.45	5.0 ± 1.73	5.6 ± 2.88	6.1 ± 2.52
Plot4	2.7 ± 1.37	4.8 ± 3.40	3.3 ± 2.31	3.5 ± 2.46	2.9 ± 1.62
Plot5	2.7 ± 3.14	4.2 ± 3.77	3.0 ± 3.46	3.3 ± 3.30	2.8 ± 3.03
Plot6	6.2 ± 3.31	8.0 ± 2.45	8.3 ± 2.89	6.9 ± 3.00	6.9 ± 3.18
Plot7	7.3 ± 2.34	5.3 ± 2.52	6.5 ± 2.12	6.7 ± 2.45	7.1 ± 2.17
Plot8	7.3 ± 2.16	7.3 ± 1.53	8.0 ± 1.41	7.3 ± 1.87	7.5 ± 1.93
Plot9	7.7 ± 1.97	2.8 ± 1.71	2.0 ± 1.00	5.7 ± 3.09	5.8 ± 3.27
Plot10	5.0 ± 2.76	6.0 ± 2.94	6.7 ± 3.21	5.4 ± 2.72	5.6 ± 2.83

Table SB10 Spearman correlations and their significance in parentheses of Table SB8 between eDNA datasets for ranking plots to minimize the loss of overall γ diversity among the remaining plots.

	16S	18S	*trn*L	ITS	COI	COI-spun	seedlings	trees	inverts
18S	0.41 (0.25)								
trnL	0.31 (0.39)	0.60 (0.073)							
ITS	0.26 (0.47)	0.37 (0.30)	0.39 (0.26)						
COI	0.44 (0.20)	0.64 (0.054)	0.45 (0.19)	0.49 (0.15)					
COI-spun	-0.33 (0.35)	-0.16 (0.66)	-0.48 (0.17)	0.37 (0.30)	-0.067 (0.86)				
seedlings	0.31 (0.39)	0.33 (0.35)	0.0061 (1.0)	0.56 (0.096)	0.16 (0.66)	0.37 (0.30)			
trees	0.26 (0.47)	0.21 (0.56)	0.018 (0.97)	0.26 (0.47)	0.19 (0.61)	0.21 (0.56)	0.70 (0.031)		
inverts	-0.048 (0.93)	0.14 (0.75)	-0.43 (0.30)	-0.40 (0.33)	-0.60 (0.13)	0.048 (0.93)	0.12 (0.79)	-0.19 (0.66)	
birds	-0.27 (0.45)	-0.56 (0.096)	-0.27 (0.45)	-0.48 (0.17)	-0.35 (0.33)	-0.0061 (1.0)	-0.19 (0.61)	-0.18 (0.63)	0 (1.0)

Table SB11 Ranking sampling plots by removing plots sequentially so as to maximize the resulting effective β diversity among the remaining plots from Figure SB13. 1 is the most important and is removed last, 10 is the least important and is removed at the beginning.

Plot name	16S	18S	*trn*L	ITS	COI	COI-spun	seedlings	trees	inverts	birds
Plot1	2	2	2	2	4	2	2	2	2	3
Plot2	7	9	10	8	8	6	8	7	7	8
Plot3	4	4	5	3	5	8	5	5	3	2
Plot4	3	5	3	6	2	7	6	6	5	5
Plot5	10	10	8	10	10	10	10	10	8	1
Plot6	9	7	6	5	7	4	4	3	6	7
Plot7	6	1	4	7	1	3	7	9		4
Plot8	8	6	9	4	9	5	9	8		6
Plot9	5	8	1	9	3	9	3	1	1	10
Plot10	1	3	7	1	6	1	1	4	4	9

Table SB12 Means and standard deviations of Table SB11 between eDNA datasets for ranking plots to maximize effective β diversity among the remaining plots.

	eDNA	Traditional	Traditional No Birds	All	All No Birds
Plot1	2.3 ± 0.816	2.2 ± 0.500	2.0 ± 0	2.3 ± 0.675	2.2 ± 0.667
Plot2	8.0 ± 1.41	7.5 ± 0.577	7.3 ± 0.577	7.8 ± 1.14	7.8 ± 1.20
Plot3	4.8 ± 1.72	3.8 ± 1.50	4.3 ± 1.15	4.4 ± 1.65	4.7 ± 1.50
Plot4	4.3 ± 1.97	5.5 ± 0.577	5.7 ± 0.577	4.8 ± 1.62	4.8 ± 1.72
Plot5	9.7 ± 0.816	7.2 ± 4.27	9.3 ± 1.15	8.7 ± 2.83	9.6 ± 0.882
Plot6	6.3 ± 1.75	5.0 ± 1.83	4.3 ± 1.53	5.8 ± 1.81	5.7 ± 1.87
Plot7	3.7 ± 2.50	6.7 ± 2.52	8.0 ± 1.41	4.7 ± 2.78	4.8 ± 2.96
Plot8	6.8 ± 2.14	7.7 ± 1.53	8.5 ± 0.707	7.1 ± 1.90	7.2 ± 1.98
Plot9	5.8 ± 3.37	3.8 ± 4.27	1.7 ± 1.15	5.0 ± 3.68	4.4 ± 3.43
Plot10	3.2 ± 2.71	4.5 ± 3.32	3.0 ± 1.73	3.7 ± 2.87	3.1 ± 2.32

Table SB13 Spearman correlations and their significance in parentheses of Table SB11 between eDNA datasets for ranking plots to maximize effective β diversity among the remaining plots.

	16S	18S	trnL	ITS	COI	COI-spun	seedlings	trees	inverts
18S	0.67 (0.039)								
trnL	0.52 (0.13)	0.43 (0.22)							
ITS	0.64 (0.054)	0.68 (0.035)	0.055 (0.89)						
COI	0.60 (0.073)	0.65 (0.049)	0.83 (0.0056)	0.091 (0.81)					
COI-spun	0.44 (0.20)	0.71 (0.028)	-0.0061 (1.0)	0.71 (0.028)	0.20 (0.58)				
seedlings	0.75 (0.018)	0.48 (0.17)	0.58 (0.088)	0.60 (0.073)	0.45 (0.19)	0.50 (0.14)			
trees	0.52 (0.13)	0.15 (0.68)	0.60 (0.073)	0.38 (0.28)	0.33 (0.35)	0.21 (0.56)	0.88 (0.0020)		
inverts	0.62 (0.11)	0.62 (0.11)	0.86 (0.011)	0.40 (0.33)	0.76 (0.037)	0.14 (0.75)	0.74 (0.046)	0.83 (0.015)	
birds	-0.16 (0.66)	0.18 (0.63)	0.018 (0.97)	-0.0061 (1.0)	-0.067 (0.86)	-0.22 (0.54)	-0.41 (0.25)	-0.48 (0.17)	-0.33 (0.43)

Table SB14 Distance-based redundancy analysis and their ANOVA tests in each step for the eDNA biodiversity datasets (16S, 18S, *trn*L).

	All			VIF< 10	Forward	Backward
16S	Inertia	Proportion	Pr(>F)	Pr(>F)	Pr(>F)	Pr(>F)
Elevation	0.48	9.8%	0.01			
Slope	0.43	8.8%	0.06	0.006		
Aspect	0.36	7.5%	0.14			
Mean.Temp	0.42	8.6%	0.09	0.028	0.001	0.015
cos.aspect	0.45	9.2%	0.03	0.052		
sin.aspect	0.43	8.8%	0.07	0.001		
Water.Content	0.50	10.2%	0.02			
pH	0.94	19.2%	0.01	0.008	0.001	0.001
EC	0.61	12.5%	0.01			
Organic.C	0.56	11.5%	0.01			
Total.N	0.52	10.7%	0.01			
C.N.ratio	0.44	9.0%	0.04	0.142		
NO3.N	0.28	5.7%	0.31	0.433		
NH4.N	0.50	10.1%	0.03	0.139		
Olsen.P	0.67	13.8%	0.01	0.593	0.058	0.001
18S	Inertia	Proportion	Pr(>F)	Pr(>F)	Pr(>F)	Pr(>F)
Elevation	0.64	9.14%	0.01			
Slope	0.62	8.87%	0.01	0.004	0.007	0.003
Aspect	0.45	6.46%	0.13			
Mean.Temp	0.56	8.01%	0.04	0.08		0.067
cos.aspect	0.37	5.23%	0.42	0.288		
sin.aspect	0.36	5.14%	0.47	0.191		0.249
Water.Content	0.63	8.98%	0.01			
pH	0.60	8.53%	0.01	0.022	0.047	0.023
EC	0.45	6.48%	0.14			
Organic.C	0.55	7.81%	0.04			
Total.N	0.55	7.83%	0.03			
C.N.ratio	0.41	5.91%	0.23	0.037		
NO3.N	0.38	5.42%	0.45	0.764		0.356
NH4.N	0.46	6.56%	0.11	0.011		
Olsen.P	0.47	6.64%	0.11	0.36	0.043	0.002
*trn*L	Inertia	Proportion	Pr(>F)	Pr(>F)	Pr(>F)	Pr(>F)
Elevation	0.63	7.40%	0.01			
Slope	0.63	7.33%	0.01	0.001	0.001	0.001
Aspect	0.56	6.58%	0.01			
Mean.Temp	0.60	6.96%	0.01	0.008		0.005
cos.aspect	0.68	7.95%	0.01	0.001		0.001
sin.aspect	0.54	6.25%	0.07	0.009		
Water.Content	0.68	7.93%	0.01			
pH	0.73	8.47%	0.01	0.043	0.001	0.002
EC	0.62	7.27%	0.01			
Organic.C	0.67	7.83%	0.01			
Total.N	0.66	7.70%	0.01			
C.N.ratio	0.51	5.94%	0.10	0.508		
NO3.N	0.57	6.61%	0.03	0.072		
NH4.N	0.60	7.00%	0.01	0.513		
Olsen.P	0.66	7.69%	0.01	0.145	0.048	0.017

Table SB15 Distance-based redundancy analysis and the ANOVA tests in each step for the eDNA biodiversity datasets (ITS, COI-soil and COI-soil spun).

	All			VIF<10	Forward	Backward
ITS	Inertia	Proportion	Pr(>F)	Pr(>F)	Pr(>F)	Pr(>F)
Elevation	0.61	6.73%	0.01			
Slope	0.65	7.27%	0.01	0.001	0.001	0.002
Aspect	0.65	7.22%	0.01			
Mean.Temp	0.59	6.59%	0.03	0.056		
cos.aspect	0.58	6.41%	0.04	0.103		
sin.aspect	0.59	6.51%	0.04	0.043		0.01
Water.Content	0.70	7.82%	0.01			
pH	0.48	5.35%	0.36	0.111		
EC	0.68	7.51%	0.01			
Organic.C	0.72	7.99%	0.01			
Total.N	0.73	8.06%	0.01			
C.N.ratio	0.51	5.70%	0.14	0.167		
NO3.N	0.58	6.49%	0.01	0.223		0.14
NH4.N	0.68	7.59%	0.01	0.17		
Olsen.P	0.65	7.20%	0.01	0.042	0.003	0.018
COI	Inertia	Proportion	Pr(>F)	Pr(>F)	Pr(>F)	Pr(>F)
Elevation	0.61	6.80%	0.01			
Slope	0.58	6.47%	0.01	0.001		
Aspect	0.55	6.16%	0.02			
Mean.Temp	0.60	6.61%	0.01	0.004		0.001
cos.aspect	0.60	6.68%	0.01	0.007		
sin.aspect	0.52	5.80%	0.11	0.006		
Water.Content	0.65	7.26%	0.01			
pH	0.65	7.20%	0.01	0.044	0.001	0.002
EC	0.62	6.94%	0.01			
Organic.C	0.65	7.24%	0.01			
Total.N	0.64	7.14%	0.01			
C.N.ratio	0.53	5.91%	0.03	0.23		
NO3.N	0.57	6.38%	0.01	0.16		0.002
NH4.N	0.61	6.76%	0.01	0.259		0.006
Olsen.P	0.64	7.08%	0.01	0.202	0.001	0.038
COI-spun	Inertia	Proportion	Pr(>F)	Pr(>F)	Pr(>F)	Pr(>F)
Elevation	0.61	6.56%	0.01			
Slope	0.57	6.08%	0.03	0.007		0.006
Aspect	0.61	6.55%	0.01			
Mean.Temp	0.57	6.10%	0.03	0.023		0.017
cos.aspect	0.61	6.51%	0.01	0.013	0.001	0.01
sin.aspect	0.59	6.32%	0.01	0.003	0.008	0.005
Water.Content	0.60	6.42%	0.01			
pH	0.54	5.83%	0.09	0.049		
EC	0.51	5.44%	0.32			
Organic.C	0.53	5.74%	0.09			
Total.N	0.54	5.84%	0.09			
C.N.ratio	0.45	4.82%	0.88	0.895		
NO3.N	0.55	5.92%	0.07	0.046		0.01
NH4.N	0.53	5.69%	0.13	0.057		0.01
Olsen.P	0.51	5.50%	0.28	0.236	0.011	

Table SB16 The constrained and unconstrained inertia changes during distance-based redundancy analysis for the eDNA biodiversity datasets (16S, 18S, *trn*L, ITS, COI-soil and COI-soil spun).

	All		VIF< 10		Forward		Backward	
16S	Inertia	Proport.	Inertia	Proport.	Inertia	Proport.	Inertia	Proportion
Constrained	4.17	85.2%	3.02	61.7%	1.86	38.1%	1.86	38.1%
Unconstrained	0.72	14.8%	1.87	38.3%	3.03	61.9%	3.03	61.9%
18S	Inertia	Proport.	Inertia	Proport.	Inertia	Proport.	Inertia	Proportion
Constrained	5.85	83.4%	3.83	54.64%	1.59	22.7%	2.87	40.9%
Unconstrained	1.17	16.6%	3.18	45.36%	5.43	77.3%	4.15	59.1%
*trn*L	Inertia	Proport.	Inertia	Proport.	Inertia	Proport.	Inertia	Proportion
Constrained	7.01	81.9%	4.51	52.65%	1.89	22.1%	2.88	33.7%
Unconstrained	1.55	18.1%	4.05	47.35%	6.67	77.9%	5.67	66.3%
ITS	Inertia	Proport.	Inertia	Proport.	Inertia	Proport.	Inertia	Proportion
Constrained	7.30	81.1%	4.63	51.39%	1.27	14.1%	2.29	25.4%
Unconstrained	1.71	18.9%	4.38	48.61%	7.74	85.9%	6.72	74.6%
COI	Inertia	Proport.	Inertia	Proport.	Inertia	Proport.	Inertia	Proportion
Constrained	7.31	81.3%	4.57	50.77%	1.29	14.3%	2.77	30.7%
Unconstrained	1.69	18.7%	4.43	49.23%	7.71	85.7%	6.24	69.3%
COI-spun	Inertia	Proport.	Inertia	Proport.	Inertia	Proport.	Inertia	Proportion
Constrained	7.50	80.4%	4.71	50.57%	1.77	19%	3.34	35.9%
Unconstrained	1.82	19.6%	4.61	49.43%	7.55	81%	5.98	64.1%
