# Supplementary material for: Evaluating a multigene environmental DNA approach for biodiversity assessment
Source: Gigascience. 2015 Oct 6;4:46. doi: 10.1186/s13742-015-0086-1 (PMC4595072; doi:10.1186/s13742-015-0086-1)
Supplement: Additional file 2 — Supplementary information A. Evaluating a multigene environmental DNA approach for biodiversity assessment. (PDF 2089 kb) [file 13742_2015_86_MOESM2_ESM.pdf]

SUPPLEMENTARY INFORMATION A

Supplementary Information A: Evaluating a multigene environmental DNA approach for biodiversity assessment

Alexei J Drummond[1,2*], Richard D Newcomb[1,3,4], Thomas R Buckley[1,3,5], Dong Xie[1,2], Andrew Dopheide[1,3,4], Benjamin CM Potter[1,3], Joseph Heled[1,2], Howard A Ross[1,3], Leah Tooman[1,4], Stefanie Grosser[1,5], Duckchul Park[5], Nicholas J Demetras[9], Mark I Stevens[6,7], James C Russell[1,3,7], Sandra H Anderson[3], Anna Carter[1,10] and Nicola Nelson[1,10]

*Correspondence:
alexei@cs.auckland.ac.nz
[1]Allan Wilson Centre, University of Auckland, Auckland, New Zealand
Full list of author information is available at the end of the article

OTU identification and informatics

The source code that was used to generate the figures and tables is available from the GitHub project eDNA-pipeline (https://github.com/walterxie/eDNA-pipeline).

OTUs and reads

Figure SA1 shows the number of OTUs (97% similarity threshold) found in K subplots for $K = 1, 2, \cdots, 20$. In the tail of the x-axis the 16S and 18S eDNA datasets contained some OTUs that appeared in all (20) of the sampled subplots, but the COI-spun dataset had no such OTUs.

Taxonomy

BLAST+ was used to classify the taxonomy of the OTUs and MEGAN 5 [1] was used to interpret and visualize the BLAST results, which are illustrated in Figure SA2-SA7 for each dataset.

Comparison of traditional and eDNA methods

As the main manuscript has described, the traditional and eDNA methods were compared in two ways: firstly, in their ability to detect community differences associated with elevation; and secondly, in a pairwise community correlation analysis. Figure SA8 and Figure SA9 show the regression of community differences against elevation differences for both eDNA and traditional datasets, respectively, using Jaccard and Horn-Morisita dissimilarity indices. Figure SA13 shows the multidimensional scaling of pairwise community matrix correlations of effective β diversity between the eDNA and traditional datasets, which is listed in Table 5.

Ranking plots by biodiversity contribution

We evaluated all of the possible combinations (210) of the four plots selected from the ten to find the subset of four that maximizes either: (i) γ or (ii) effective β diversity. The subset of the four plots with maximum γ diversity for the eDNA biodiversity datasets (16S, 18S, *trn*L, ITS, COI-soil, and COI-soil spun) and the

traditional biodiversity datasets (seedlings, trees, invertebrates, birds) is illustrated in the heat-maps in Figure SA14. Where there is more than one subset with equal maximum diversity, a lighter colour is used to show the plots involved. Plots 1 and 4 appear to be important contributors to the overall γ diversity for most datasets, and Plot 8 appears to be the least important. Plots 1, 4, and 5 are important for most eDNA datasets, and Plot 9 is important for all traditional biodiversity datasets. Plot 1 (for all data) and Plot 10 are important contributors to the effective β diversity for most datasets, and Plot 2 appears to be the least important.

To discover how the diversities varied as a function of the number of sites, we applied a greedy algorithm to remove the plots sequentially so as to minimize the loss of the selected diversity measure among the remaining plots. We used γ diversity in Figure SA15 and effective β diversity in Figure SA16. This procedure was undertaken independently for each of the ten biodiversity measures.

The ten plots are ranked in Table SA11 by contribution to retained γ diversity and Table SA14 by contribution to retained effective β diversity. Rank 1 is the most important plot and was therefore removed last, Rank 10 is the least important plot and was removed first. The means and standard deviations of these ranks are calculated, respectively, in Table SA12 and Table SA15.

Table SA12 shows that, for retained γ diversity, Plots 4 and 5 are important for the eDNA datasets and Plot 9 is important for the traditional biodiversity datasets. Plot 8 is still the least important for all of the datasets. For the effective β diversity measure shown in Table SA14, Plot 1 is most important for all of the datasets, whereas Plot 10 is only important to the eDNA datasets. Plot 5 is the least important and Plot 2 is the second least important for all of the datasets.

The Spearman correlations of those ranks among datasets are calculated in Table SA13 and Table SA16.

In summary, the eDNA methods and traditional methods produce similar patterns of rank when measured by contribution to retained effective β diversity.

Author details

[1]Allan Wilson Centre, University of Auckland, Auckland, New Zealand. [2]Department of Computer Science, University of Auckland, Private Bag 92019, Auckland 1142, New Zealand. [3]School of Biological Sciences, University of Auckland, Private Bag 92019, Auckland 1142, New Zealand. [4]The Institute for Plant and Food Research, Private Bag 92169, Auckland 1142 , New Zealand. [5]Landcare Research, Private Bag 92170, Auckland 1142 , New Zealand. [6]South Australian Museum, North Terrace, Adelaide SA 5000 , Australia. [7]School of Pharmacy and Medical Sciences, University of South Australia, GPO Box 2471, Adelaide SA 5001 , Australia. [8]Department of Statistics, University of Auckland,, Private Bag 92019, Auckland 1142 , New Zealand. [9]Department of Biological Sciences, University of Waikato, Private Bag 3105, Hamilton 3240 , New Zealand. [10]School of Biological Sciences, Victoria University of Wellington, PO Box 600, Wellington 6140 , New Zealand.

References

1. Huson DH, Mitra S, Ruscheweyh HJ, Weber N, Schuster SC. Integrative analysis of environmental sequences using MEGAN4. Genome research. 2011;21(9):1552–1560.

Figure SA1 The number of OTUs at the 97% clustering threshold across the number of sites.

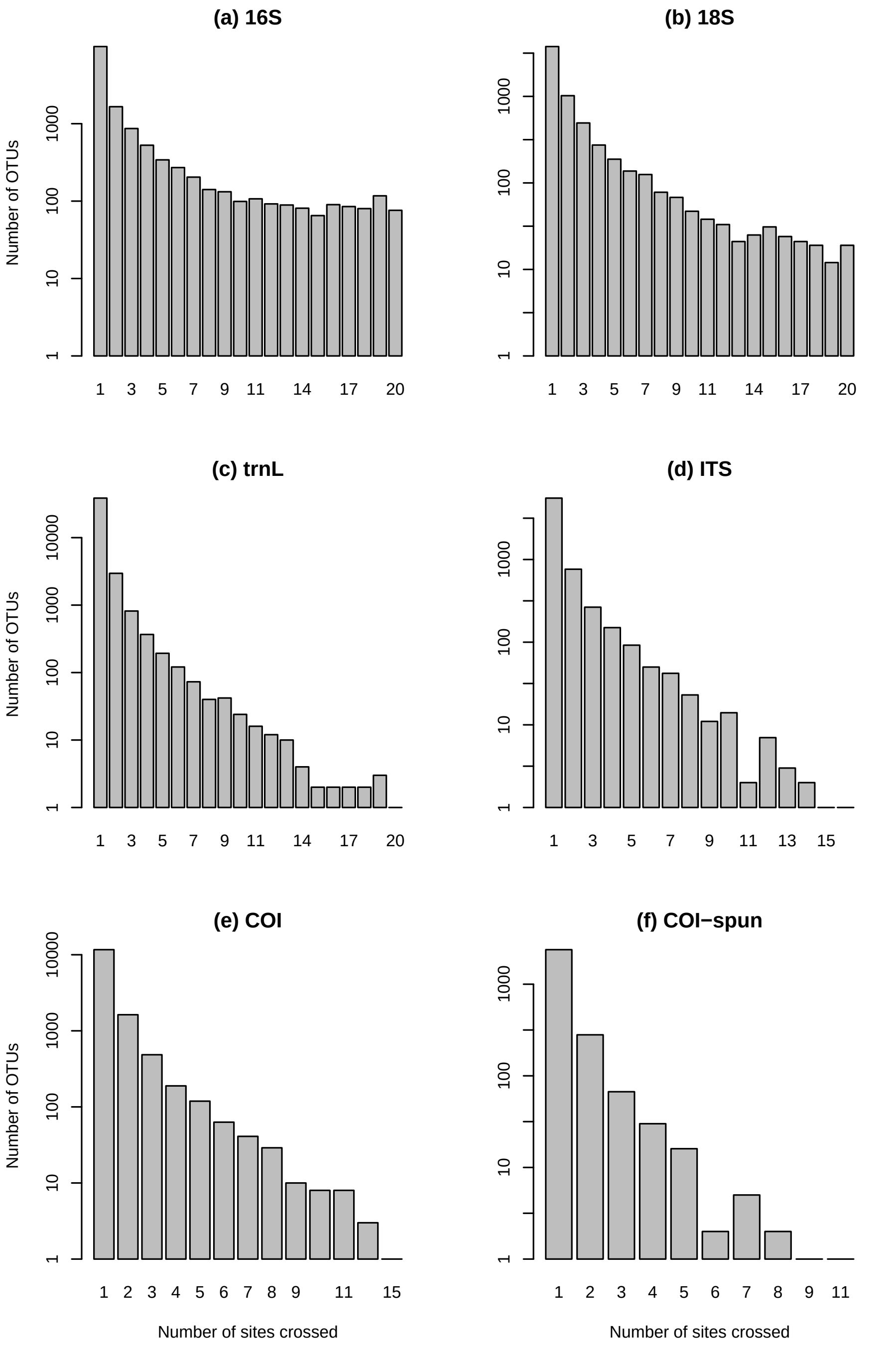

Figure SA2 MEGAN taxonomic classification of OTUs at the 97% clustering threshold assigned to phyla and equivalent high-level taxa for 16S data's BLAST result.

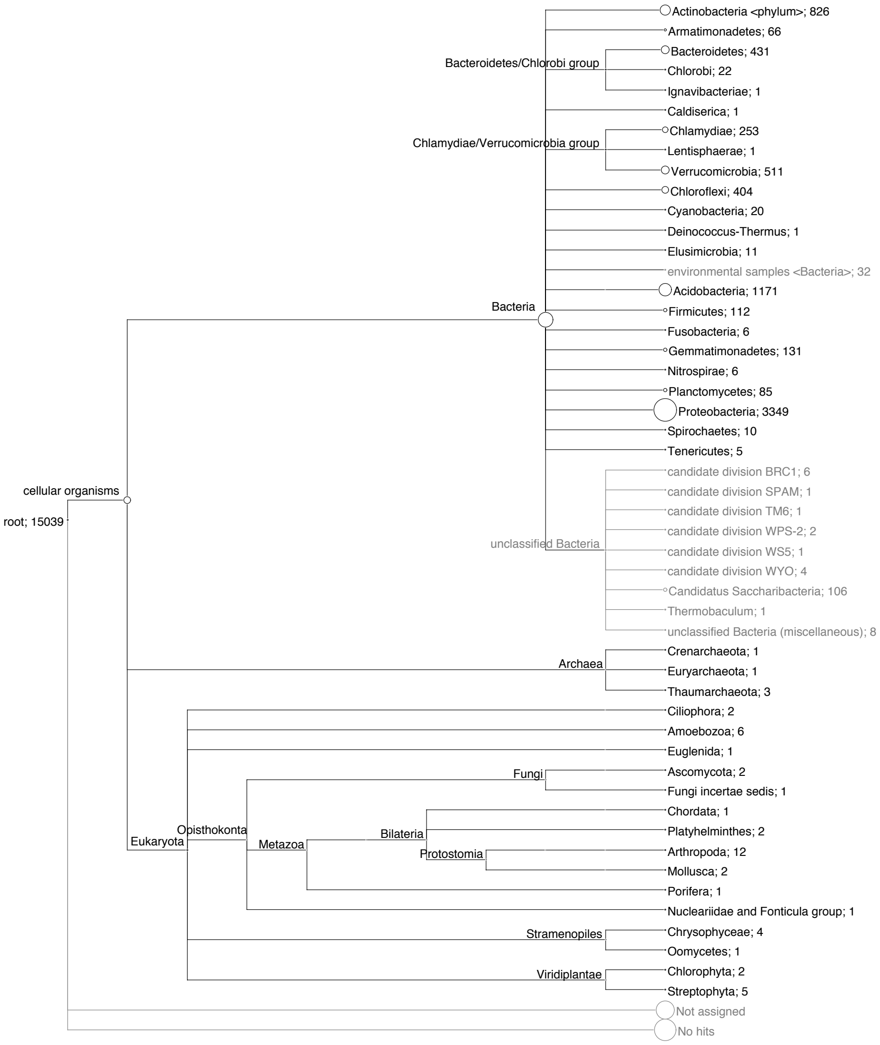

Figure SA3 MEGAN taxonomic classification of OTUs at the 97% clustering threshold assigned to phyla and equivalent high-level taxa for 18S data's BLAST result.

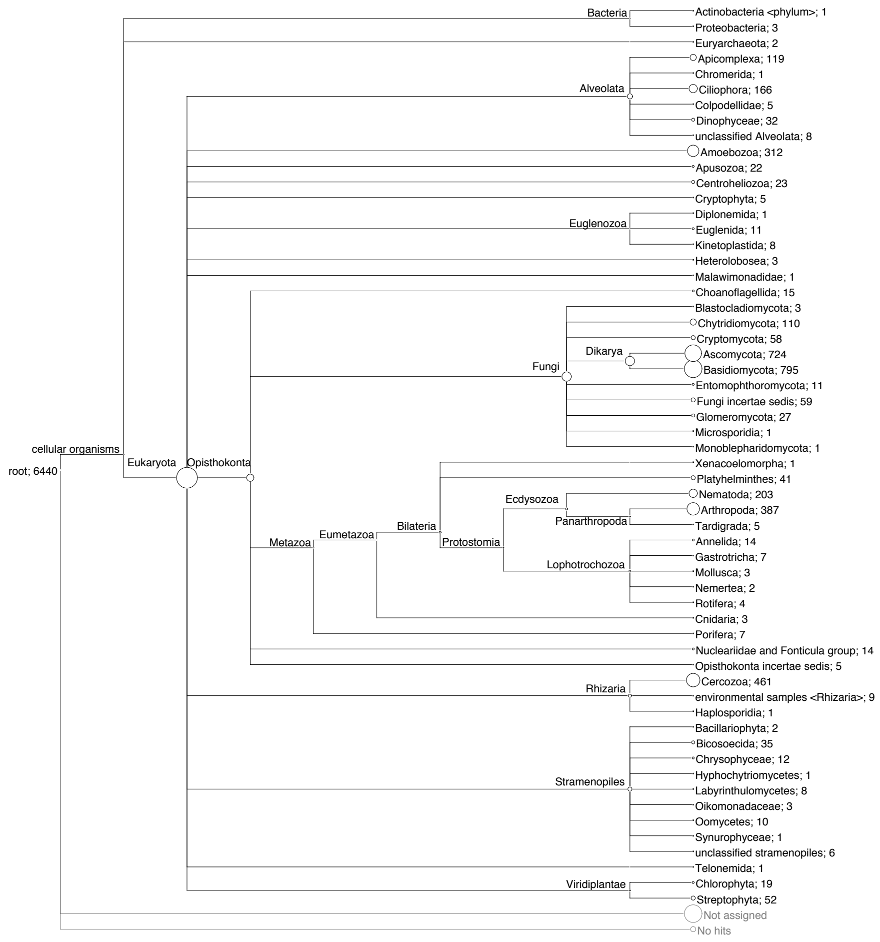

Figure SA4 MEGAN taxonomic classification of OTUs at the 97% clustering threshold assigned to phyla and equivalent high-level taxa for *trn*L data's BLAST result.

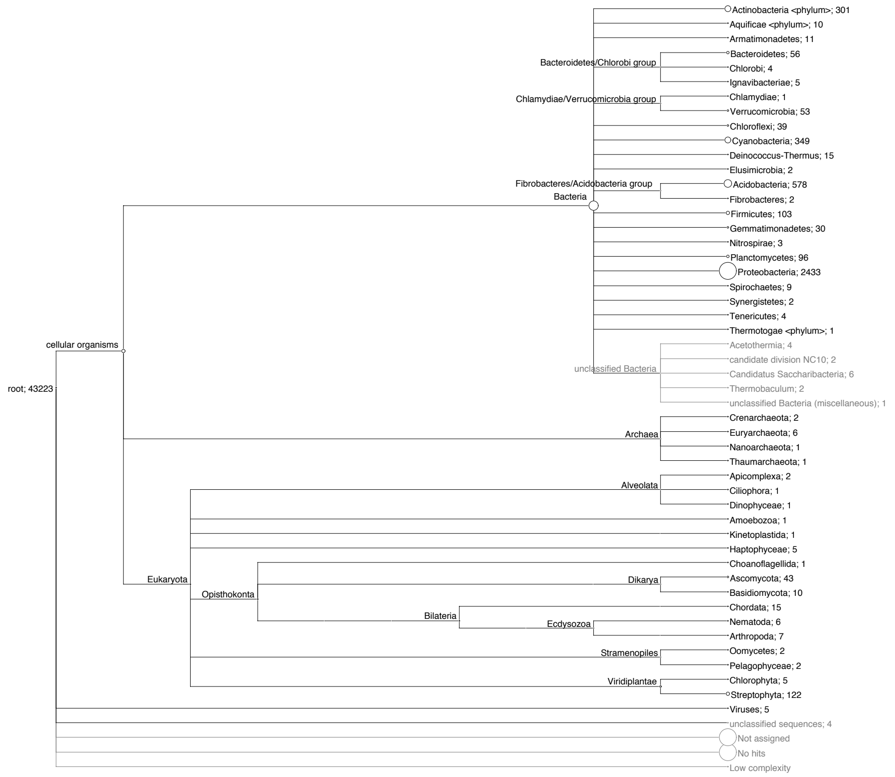

Figure SA5 MEGAN taxonomic classification of OTUs at the 97% clustering threshold assigned to phyla and equivalent high-level taxa for ITS data's BLAST result.

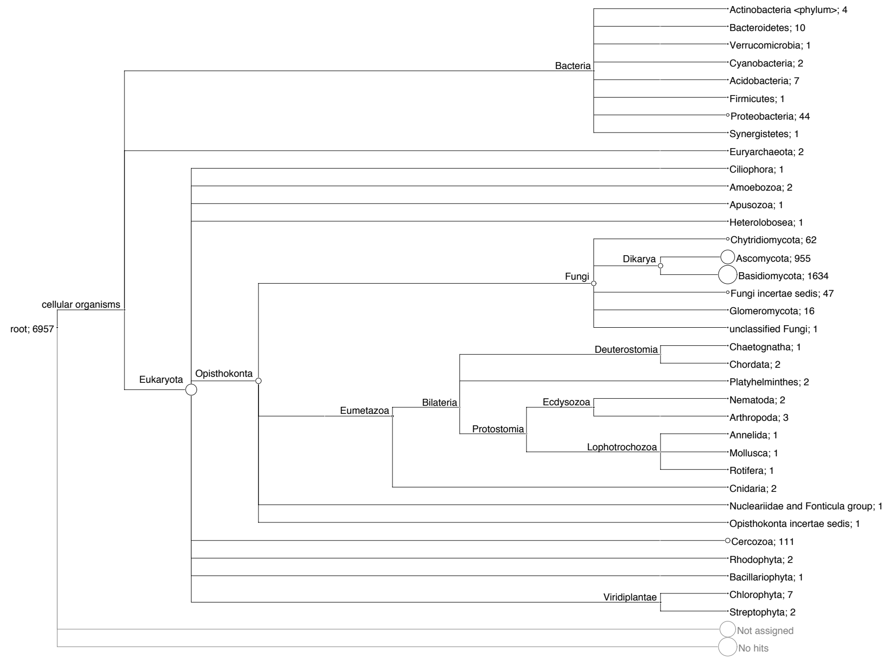

Figure SA6 MEGAN taxonomic classification of OTUs at the 97% clustering threshold assigned to phyla and equivalent high-level taxa for COI data's BLAST result.

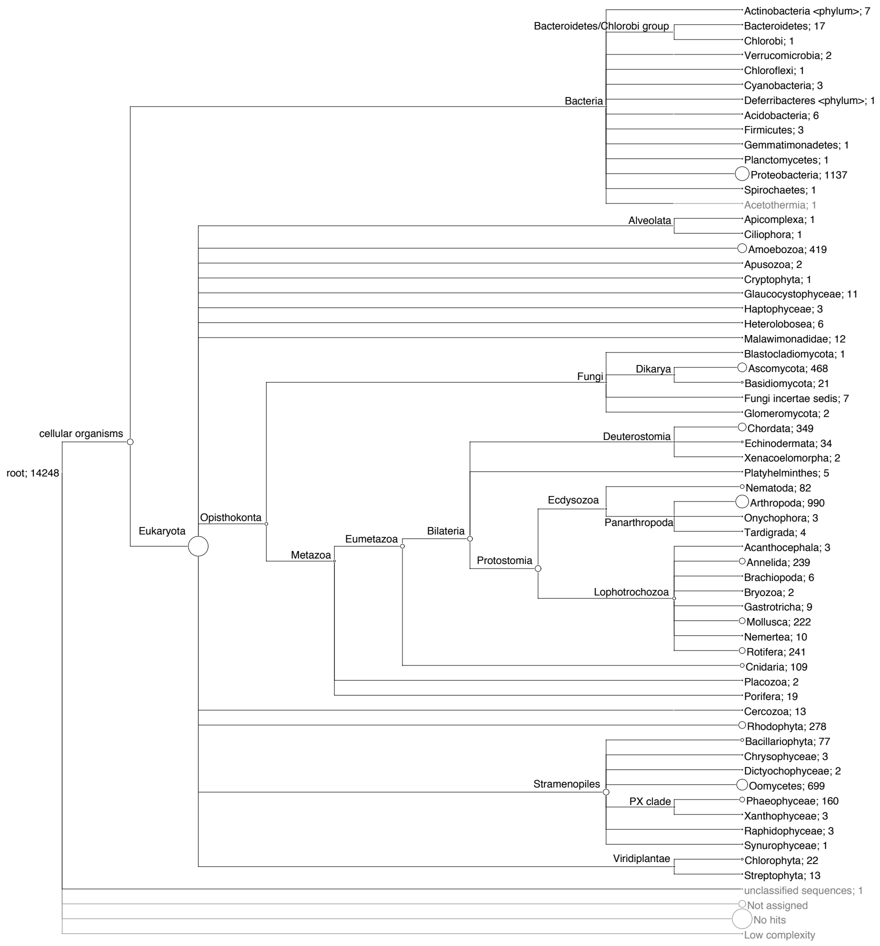

Figure SA7 MEGAN taxonomic classification of OTUs at the 97% clustering threshold assigned to phyla and equivalent high-level taxa for COI-spun data's BLAST result.

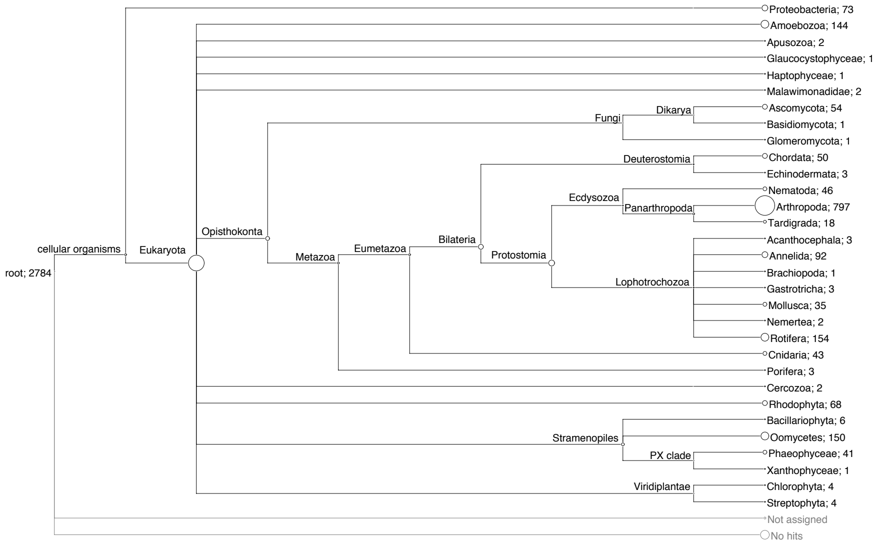

Figure SA8 Regression of difference in community measured by Jaccard index and difference in elevation for (a) the eDNA biodiversity datasets (16S, 18S, *trn*L, ITS, COI-soil and COI-soil spun) and (b) traditional biodiversity datasets (seedlings, trees, invertebrates, birds).

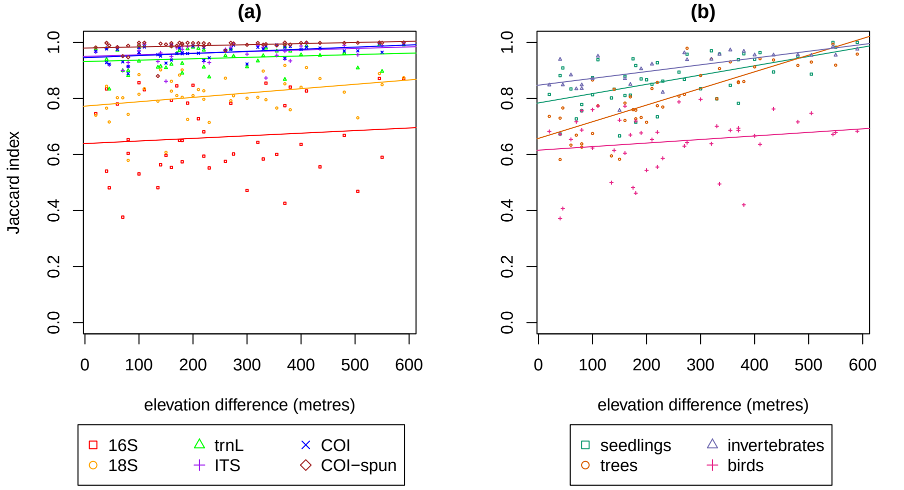

Figure SA9 Regression of difference in community measured by Horn-Morisita overlap and difference in elevation for (a) the eDNA biodiversity datasets (16S, 18S, *trn*L, ITS, COI-soil and COI-soil spun) and (b) traditional biodiversity datasets (seedlings, trees, invertebrates, birds).

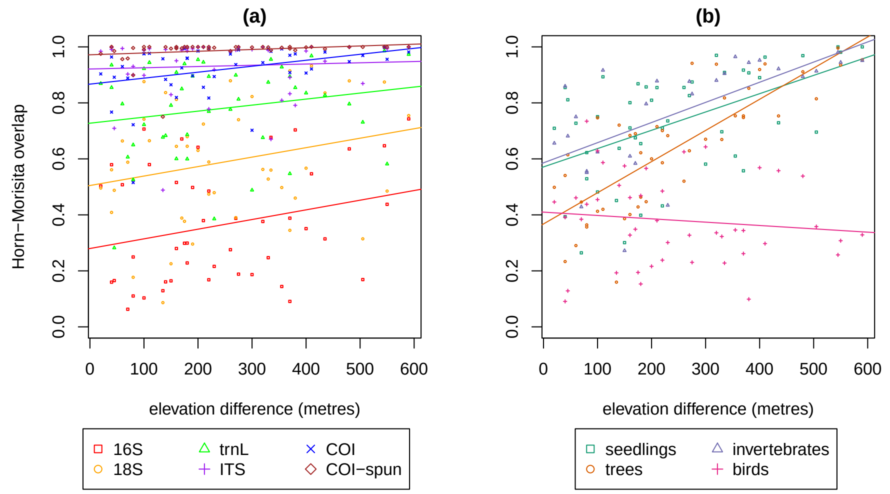

Figure SA10 Regression of effective α diversity as a function of elevation for (a) the eDNA biodiversity datasets (16S, 18S, *trn*L, ITS, COI-soil and COI-soil spun) and (b) traditional biodiversity datasets (seedlings, trees, invertebrates, birds).

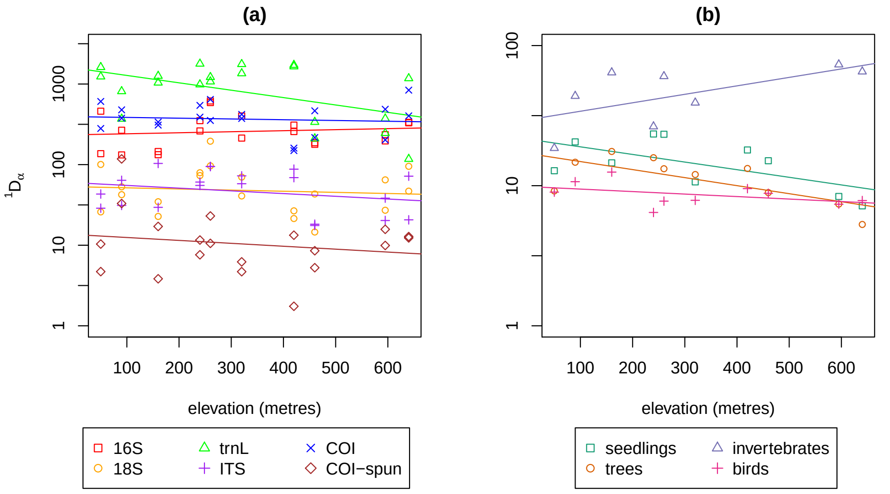

Figure SA11 Non-metric multidimensional scaling of community dissimilarity measured by Jaccard index for paired subplots for the molecular datasets 16S, 18S, *trn*L, ITS, COI-soil and COI-soil spun.

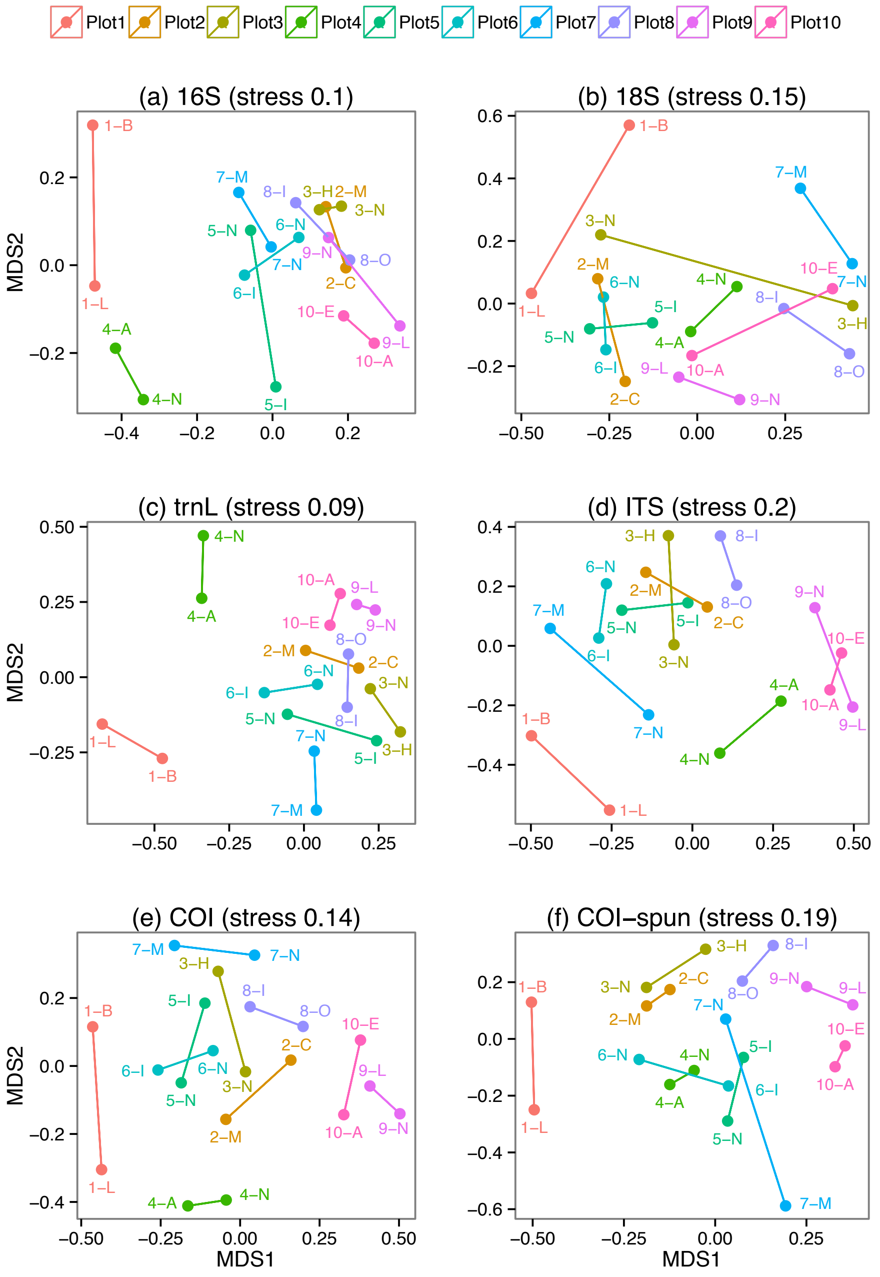

Figure SA12 Non-metric multidimensional scaling of community dissimilarity measured by Horn-Morisita overlap for paired subplots for the molecular datasets 16S, 18S, *trn*L, ITS, COI-soil and COI-soil spun.

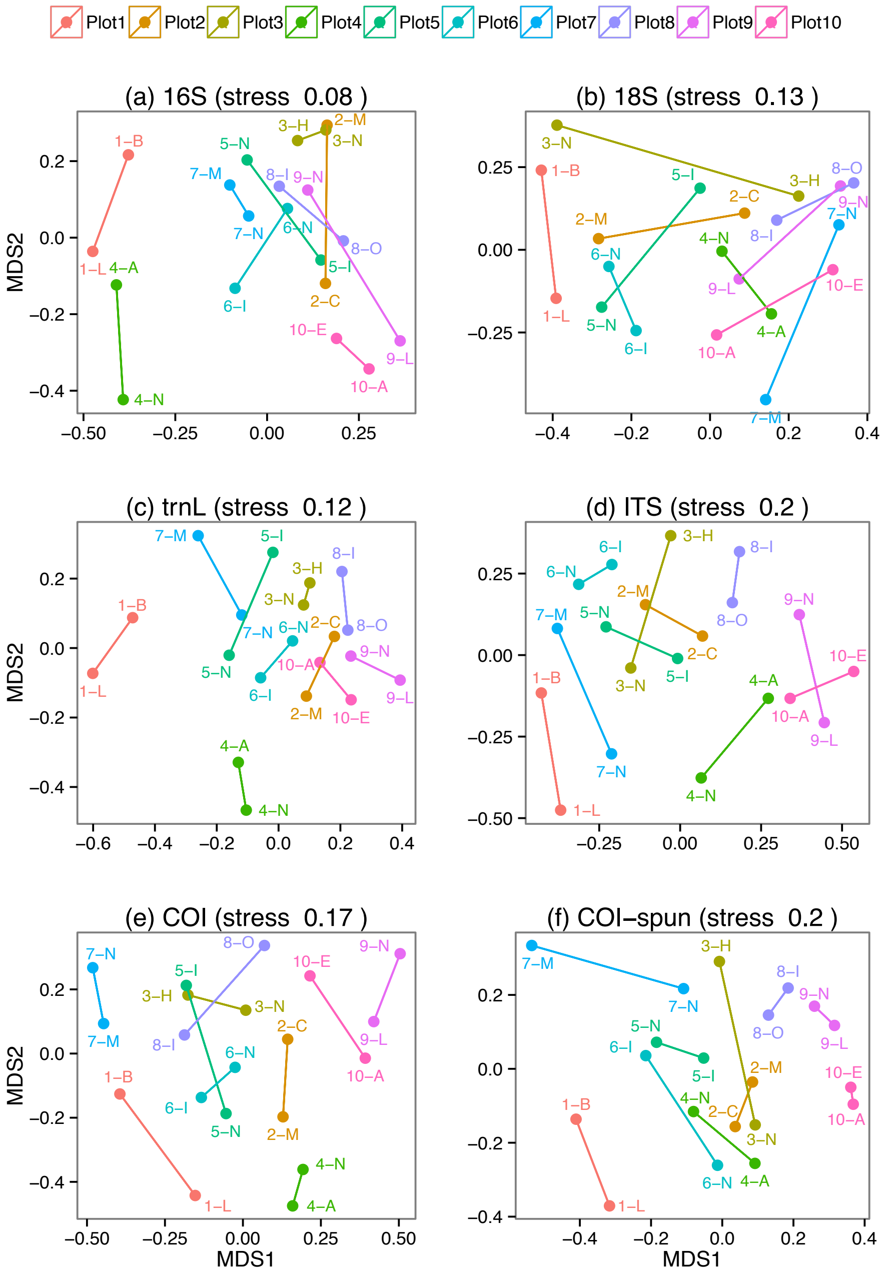

Figure SA13 Multidimensional scaling of pairwise community matrix correlations of effective β diversity within and between the eDNA datasets (16S, 18S, trnL, ITS, COI-soil and COI-soil spun) and traditional datasets (seedlings, trees, invertebrates, birds).

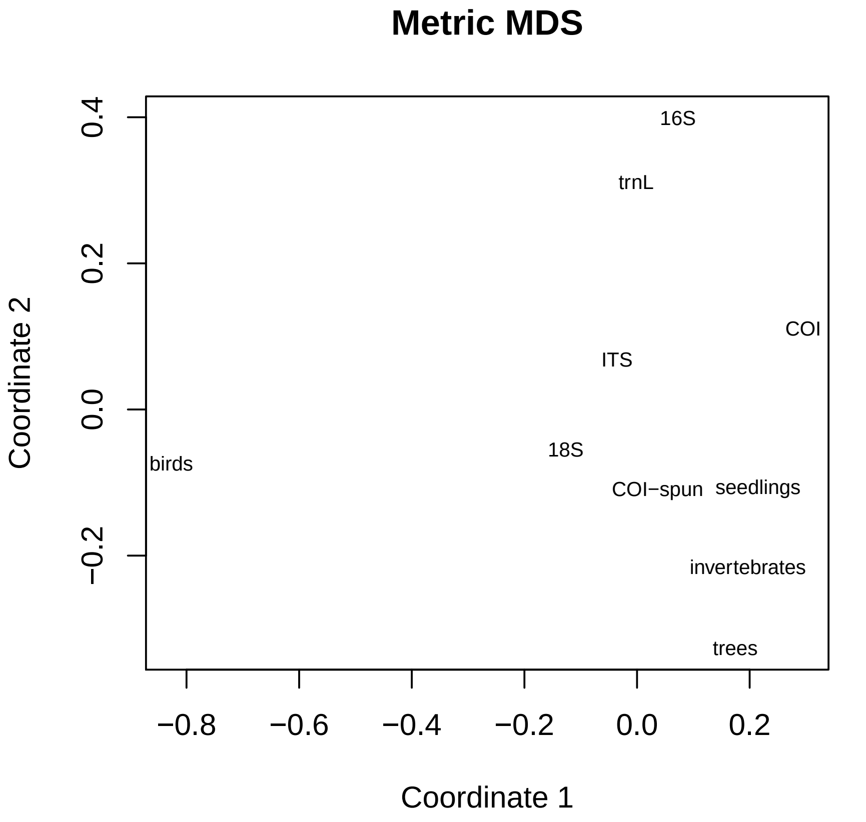

Figure SA14 The probability of having a) maximum γ diversity and b) maximum effective β diversity of all possible combinations of four plots for the eDNA biodiversity datasets (16S, 18S, *trn*L, ITS, COI-soil and COI-soil spun) and the traditional biodiversity datasets (seedlings, trees, invertebrates, birds).

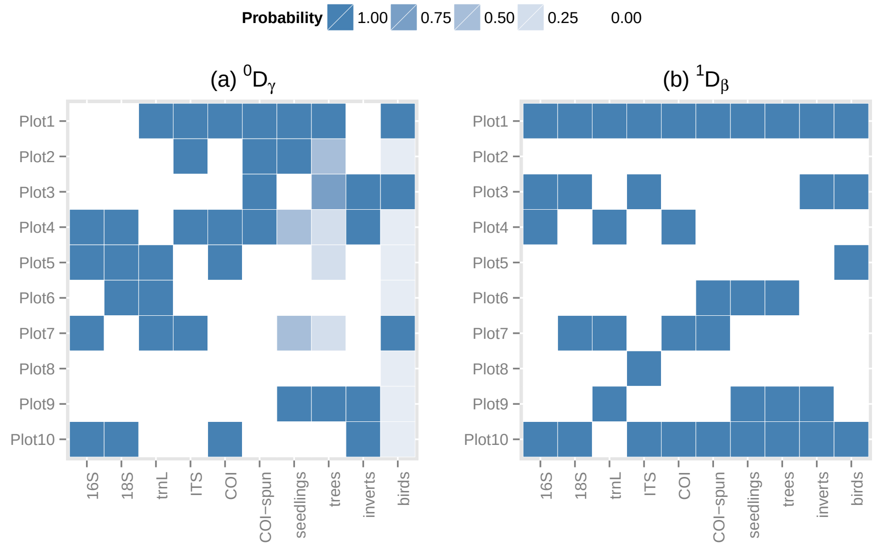

Figure SA15 Maximum remained γ diversity as a function of number of sites for (a) eDNA biodiversity datasets (16S, 18S, *trn*L, ITS, COI-soil and COI-soil spun) and (b) traditional biodiversity datasets (seedlings, trees, invertebrates, birds). The plots were removed sequentially to minimize the loss of overall γ diversity among the remaining plots. This procedure was undertaken independently for each of the ten biodiversity measures.

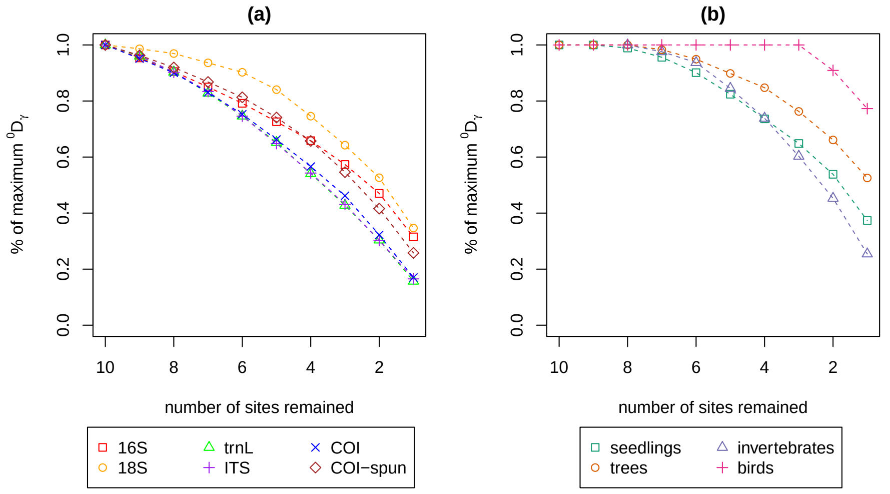

Figure SA16 Maximum remained effective β diversity as a function of the number of sites for (a) eDNA biodiversity datasets (16S, 18S, *trn*L, ITS, COI-soil, and COI-soil spun) and (b) traditional biodiversity datasets (seedlings, trees, invertebrates, birds). The plots were removed sequentially to maximize the resulting effective β diversity among the remaining plots. This procedure was undertaken independently for each of the ten biodiversity measures.

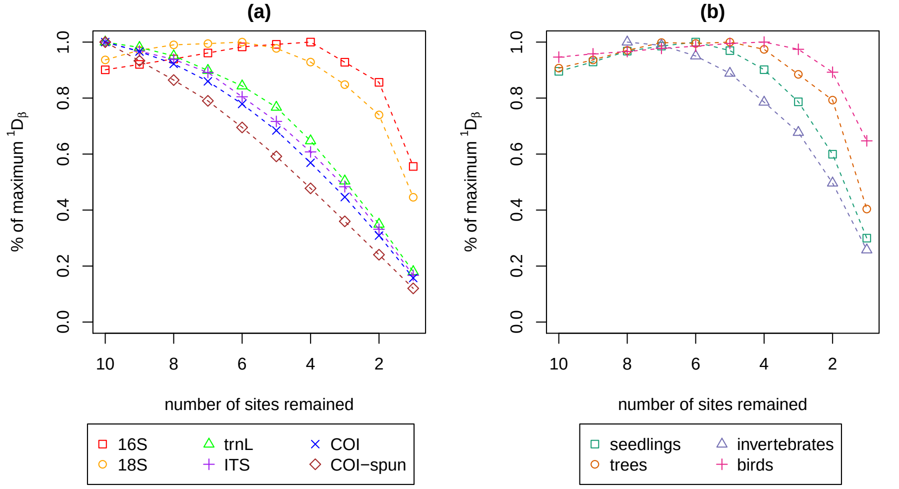

Figure SA17 Environmental data. Correlation plots of the 15 environmental variables after logarithmic transform to soil chemistry measurements, except for the pH and Carbon-Nitrogen ratio.

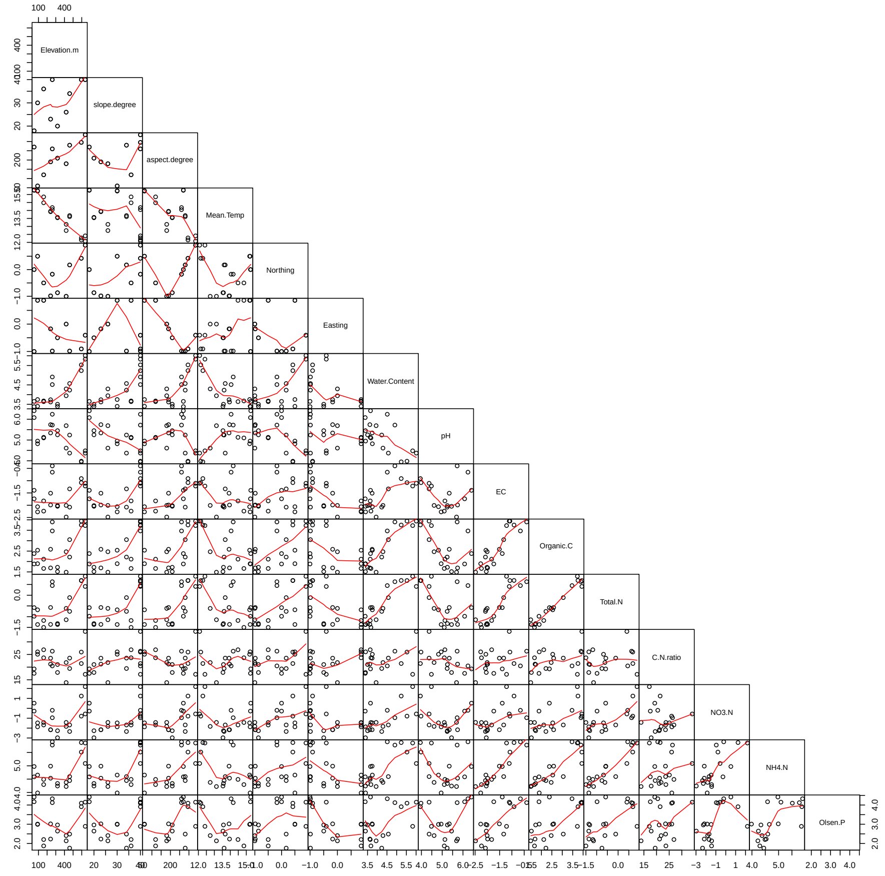

Figure SA18 Models constructed by the combined set of variables with VIF < 10 using distance-based redundancy analysis for the molecular datasets 16S, 18S, *trn*L, ITS, COI, and COI-spun.

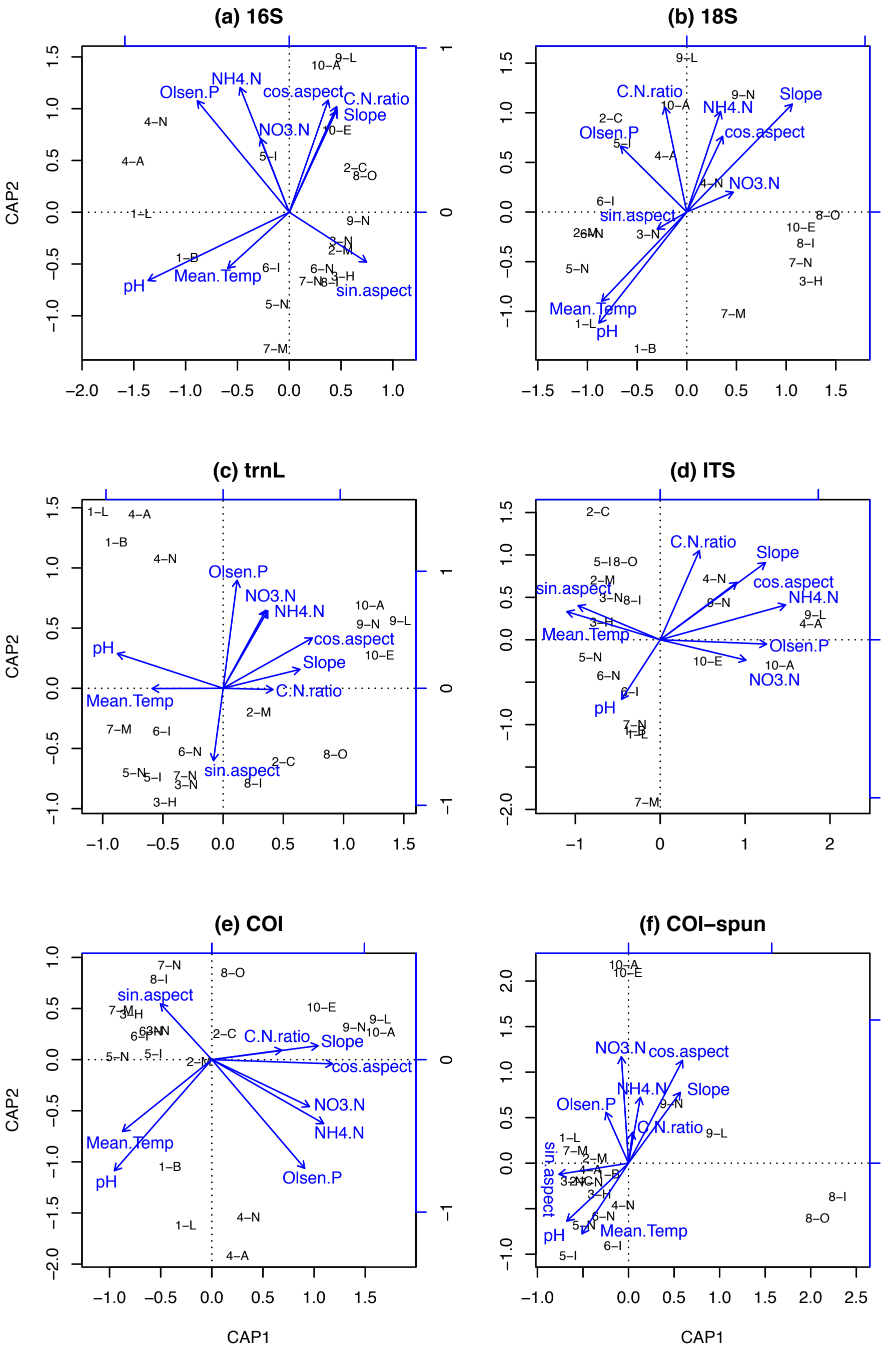

Figure SA19 More conservative models constructed by the subsets of the variables with VIF < 10 chosen by stepwise forward selection model building procedures using distance-based redundancy analysis for the molecular datasets (16S, 18S, *trn*L, ITS, COI, and COI-spun).

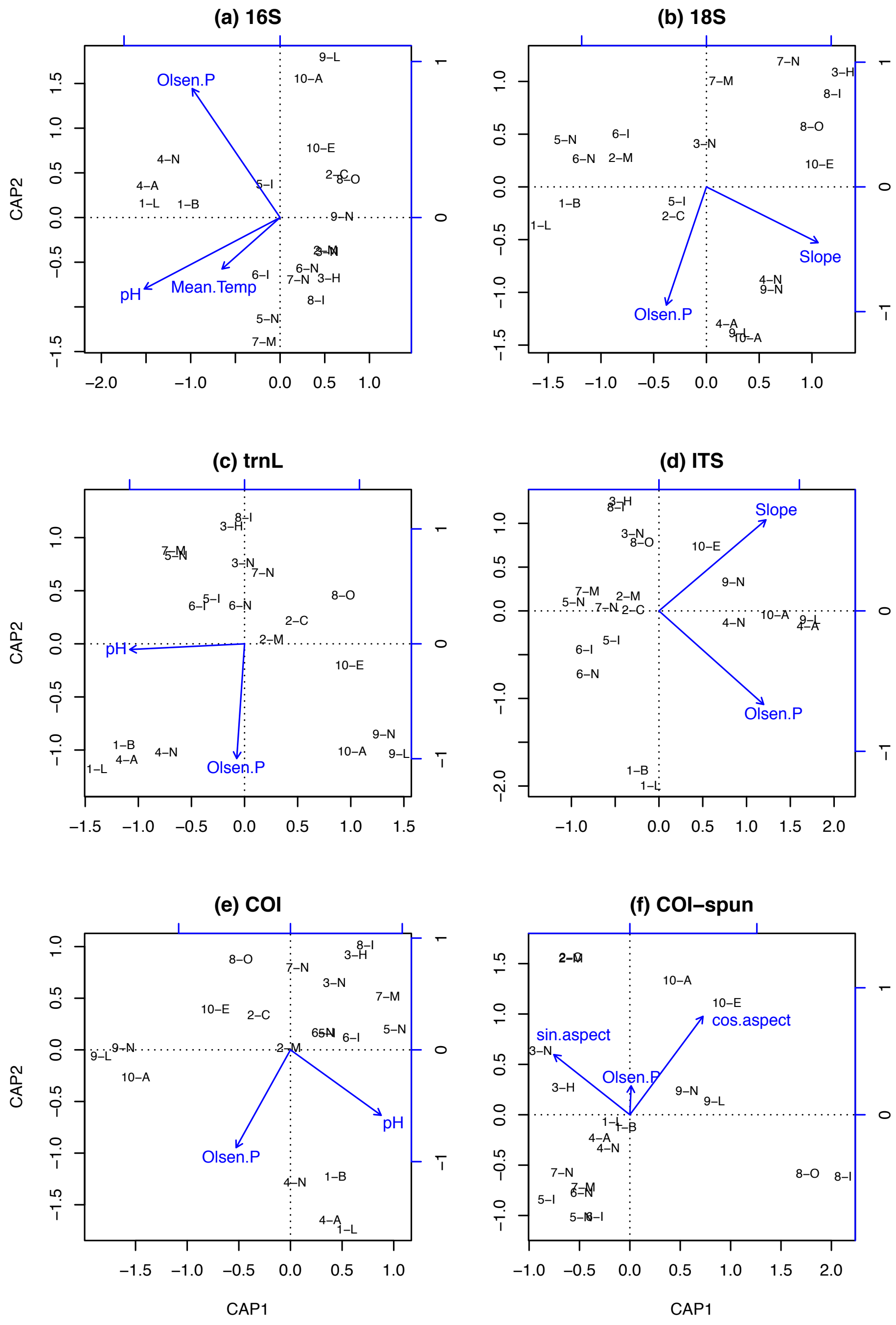

Figure SA20 More conservative models constructed by the subsets of the variables with VIF < 10 chosen by stepwise backward selection model building procedures using distance-based redundancy analysis for the molecular datasets (16S, 18S, *trn*L, ITS, COI, and COI-spun).

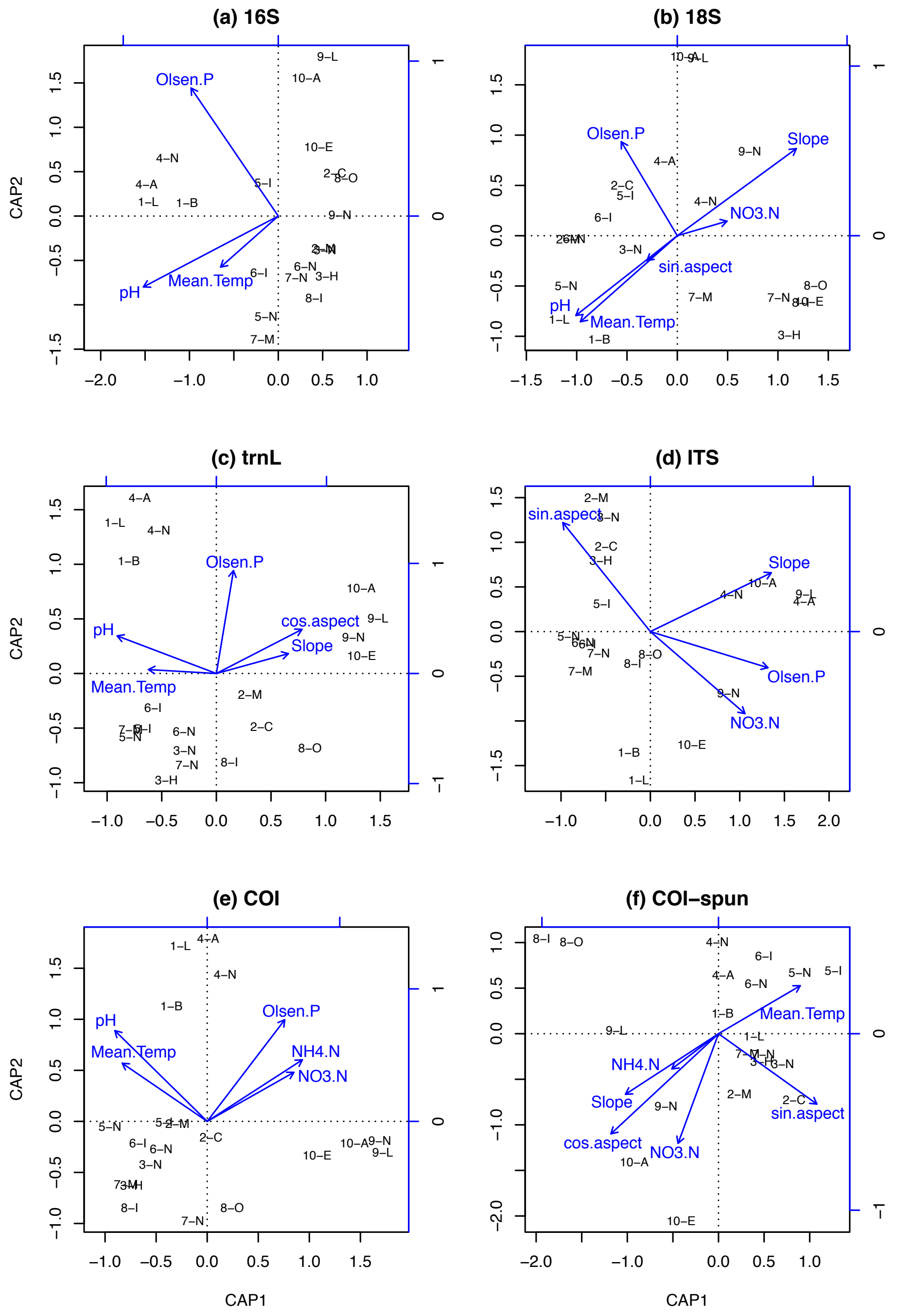

Figure SA21 Models constructed by the combined set of variables with VIF < 10, and more conservative models chosen by stepwise forward or backward selection model building procedures using distance-based redundancy analysis for the plant datasets (seedlings and trees).

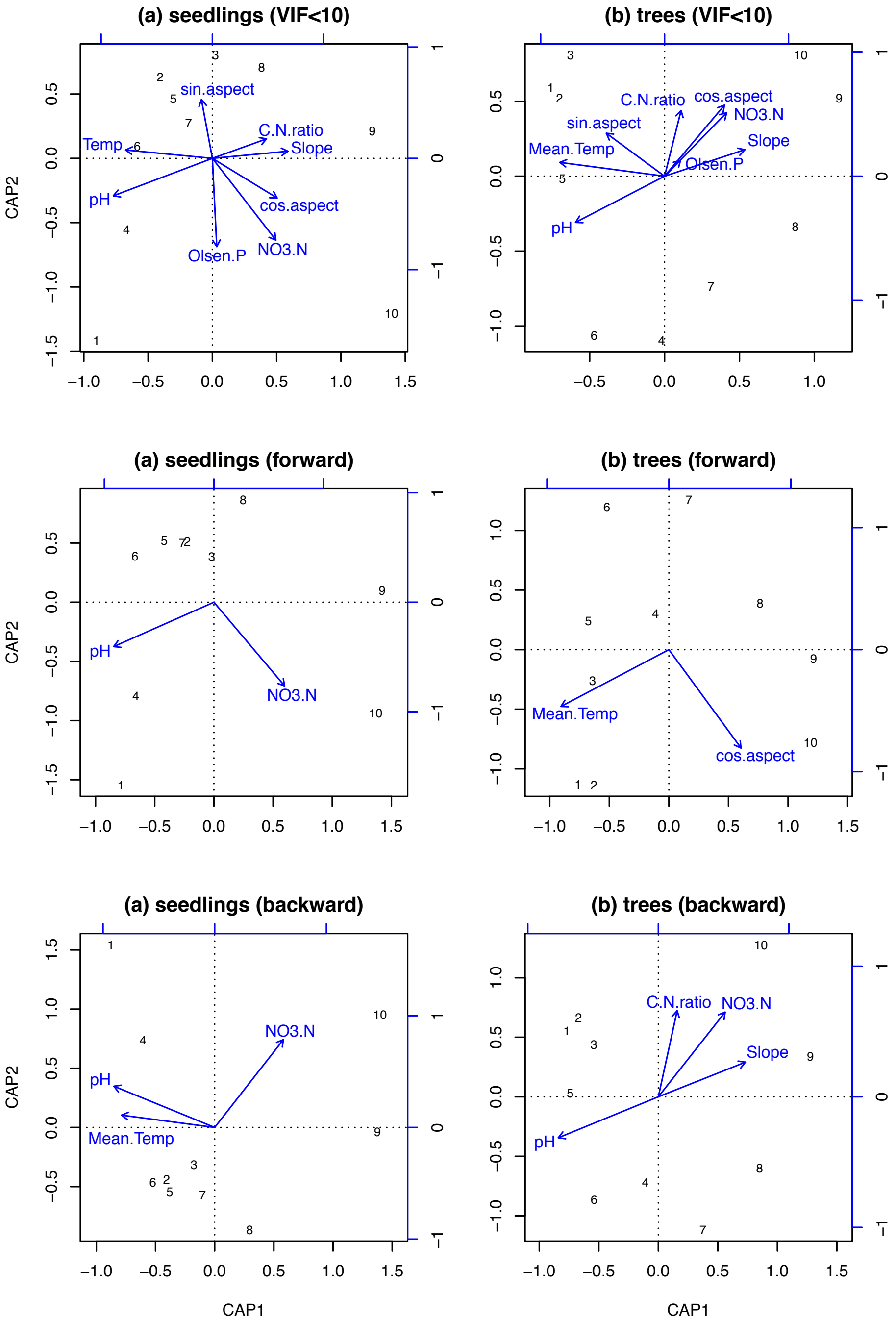

Table SA1 Table of sequences per plot for 16S, 18S, *trn*L, ITS, COI, and COI-spun molecular datasets.

	16S	18S	trnL	ITS	COI	COI-spun	Total
Plot1	64,647	27,475	16,502	10,350	10,552	11,303	140,829
Plot2	47,564	100,673	19,714	19,763	8,089	8,170	203,973
Plot3	48,262	34,213	12,757	12,813	7,248	8,012	123,305
Plot4	61,037	50,823	16,161	14,717	7,408	6,361	156,507
Plot5	93,729	86,935	16,951	18,167	10,629	4,548	230,959
Plot6	37,971	78,377	17,487	12,549	9,200	3,679	159,263
Plot7	53,431	15,079	13,906	4,344	5,978	5,383	98,121
Plot8	55,093	17,877	22,350	23,527	8,195	5,907	132,949
Plot9	50,962	74,085	12,964	11,805	7,828	5,096	162,740
Plot10	51,289	35,289	21,914	4,850	8,620	5,137	127,099

Table SA2 Table of OTUs per plot for 16S, 18S, *trn*L, ITS, COI, and COI-spun molecular datasets.

	16S	18S	trnL	ITS	COI	COI-spun	Total*
Plot1	2,781	1,189	6,552	1,007	2,344	344	14,217
Plot2	2,619	1,460	4,462	1,039	1,776	718	12,074
Plot3	2,437	963	5,166	994	1,801	464	11,825
Plot4	4,739	2,015	5,183	1,150	1,823	480	15,390
Plot5	4,281	2,233	6,105	1,118	2,426	212	16,375
Plot6	2,873	1,841	6,785	1,041	1,735	167	14,442
Plot7	2,946	568	5,632	964	1,050	153	11,313
Plot8	2,679	705	3,554	831	1,653	290	9,712
Plot9	2,577	1,188	2,297	534	1,145	198	7,939
Plot10	3,153	1,375	3,878	672	2,113	228	11,419

* the total number of OTUs is just each number for each dataset added together.

Table SA3 Table of Shannon index per plot for 16S, 18S, *trn*L, ITS, COI, and COI-spun molecular datasets.

	16S	18S	trnL	ITS	COI	COI-spun
Plot1	276.24	103.38	2,379.34	61.61	768.13	12.61
Plot2	211.25	58.98	889.48	71.21	779.77	111.20
Plot3	155.16	43.39	1,890.03	107.57	518.60	16.16
Plot4	663.09	196.26	1,967.64	164.57	813.71	29.98
Plot5	370.61	104.45	2,355.96	98.43	881.03	15.87
Plot6	345.75	73.23	2,605.78	104.56	677.12	10.23
Plot7	315.69	33.06	2,822.58	138.23	240.27	6.07
Plot8	206.70	33.85	414.90	22.01	586.96	8.40
Plot9	263.84	57.80	440.73	51.53	496.35	20.70
Plot10	379.72	102.08	526.29	64.55	959.68	19.79

Table SA4 Table of all sampled individuals per plot for seedlings, tree, invertebrates, and birds.

	seedlings†	trees	inverts	birds	Total
Plot1	114	405	56	110	685
Plot2	198	423	172	182	975
Plot3	110	257	159	90	616
Plot4	194	260	181	96	731
Plot5	138	291	130	99	658
Plot6	66	266	252	153	737
Plot7	214	521	NA*	70	805
Plot8	172	453	NA*	70	695
Plot9	58	373	213	56	700
Plot10	38	271	243	73	625

* NA: invertebrate data is missing for Plots 7 and 8.
† These values correspond to presence-absence totals from the 24 seedling subplots (0.75m^2), rather than the full plots.

Table SA5 Table of species or OTUs per plot for seedlings, tree, invertebrates, and birds

	seedlings	trees	inverts	birds	Total†
Plot1	22	21	28	15	86
Plot2	34	30	76	15	155
Plot3	25	28	84	17	154
Plot4	33	25	91	11	160
Plot5	34	31	58	12	135
Plot6	16	21	85	13	135
Plot7	31	23	NA*	13	67
Plot8	26	23	NA*	10	59
Plot9	15	17	105	9	146
Plot10	10	17	99	10	136

* NA: invertebrate data is missing for Plots 7 and 8.
† The total number of OTUs is just each number for each dataset added together.

Table SA6 Table of Shannon index per plot for seedlings, tree, invertebrates, and birds

	seedlings	trees	inverts	birds
Plot1	12.79	9.18	18.54	9.00
Plot2	20.55	14.71	43.63	10.68
Plot3	14.56	17.55	64.21	12.51
Plot4	23.28	13.21	60.36	7.76
Plot5	23.39	15.84	26.45	6.44
Plot6	10.65	12.01	39.09	7.86
Plot7	17.99	13.27	NA*	9.53
Plot8	15.07	8.93	NA*	8.82
Plot9	8.37	7.39	73.20	7.38
Plot10	7.18	5.29	65.19	7.83

* NA: invertebrate data is missing for Plots 7 and 8.

Table SA7 Mantel statistic r and their significance using Mantel's test based on 4,999 permutations, and R^2 and $p\text{-}value$ for a linear model of the regression of difference in community measured by Jaccard and difference in elevation in Figure SA8.

	Mantel statistic r	significance	R^2	$p\text{-}value$
16S	0.103	0.285	0.0106	0.500
18S	0.33	0.0192	0.109	0.0267
trnL	0.191	0.1422	0.0364	0.209
ITS	0.262	0.0592	0.0686	0.0821
COI	0.464	0.0036	0.215	0.00132
COI-spun	0.302	0.0108	0.0911	0.0439
seedlings	0.592	8e-04	0.351	1.82e-05
trees	0.805	2e-04	0.648	2.68e-11
invertebrates	0.711	0.006	0.505	2.26e-05
birds	0.186	0.1482	0.0346	0.221

Table SA8 Mantel statistic r and their significance using Mantel's test based on 4,999 permutations, and R^2 and p-$value$ for a linear model of the regression of difference in community measured by Horn-Morisita and difference in elevation in Figure SA9.

	Mantel statistic r	significance	R^2	p-$value$
16S	0.259	0.0796	0.0671	0.0858
18S	0.247	0.0726	0.0609	0.102
trnL	0.196	0.1256	0.0384	0.197
ITS	0.063	0.3598	0.00400	0.680
COI	0.345	0.013	0.119	0.0201
COI-spun	0.24	0.0378	0.0577	0.112
seedlings	0.516	0.001	0.266	2.87e-04
trees	0.793	2e-04	0.629	8.26e-11
invertebrates	0.652	0.0112	0.425	1.7e-04
birds	-0.116	0.7134	0.0134	0.449

Table SA9 The significance test of effective α diversity as a function of elevation in Figure SA10.

	R^2	p-$value$
16S	0.0151	0.606
18S	0.00967	0.680
trnL	0.255	0.0230
ITS	0.0668	0.271
COI	0.00919	0.688
COI-spun	0.0347	0.432
seedlings	0.379	0.0582
trees	0.501	0.0219
invertebrates	0.386	0.100
birds	0.185	0.215

Table SA10 Pairwise community matrix Procrustes analysis of effective β diversity within and between the eDNA datasets and traditional datasets, estimated as sum of the squared differences and their significance in parentheses is based on 4,999 permutations.

	16S	18S	*trn*L	ITS	COI	COI-spun	seedlings	trees	inverts
18S	0.57 (0.021)								
trnL	0.35 (0.00060)	0.55 (0.017)							
ITS	0.59 (0.019)	0.65 (0.069)	0.34 (0.0016)						
COI	0.44 (0.0014)	0.47 (0.0028)	0.12 (0.00040)	0.47 (0.010)					
COI-spun	0.82 (0.37)	0.70 (0.11)	0.85 (0.52)	0.78 (0.27)	0.78 (0.27)				
seedlings	0.49 (0.011)	0.41 (0.0016)	0.50 (0.0068)	0.47 (0.0056)	0.44 (0.0038)	0.81 (0.37)			
trees	0.63 (0.046)	0.45 (0.0018)	0.69 (0.096)	0.72 (0.13)	0.61 (0.039)	0.53 (0.016)	0.42 (0.0020)		
inverts	0.68 (0.14)	0.41 (0.012)	0.33 (0.010)	0.36 (0.0070)	0.26 (0.0052)	0.70 (0.22)	0.28 (0.0014)	0.54 (0.052)	
birds	0.88 (0.54)	0.56 (0.016)	0.87 (0.56)	0.85 (0.48)	0.83 (0.41)	0.66 (0.063)	0.75 (0.19)	0.67 (0.058)	0.63 (0.13)

Table SA11 Ranking sampling plots by removing plots sequentially so as to minimize the loss of overall γ diversity among the remaining plots from Figure SA15. 1 is the most important and is removed last, 10 is the least important and is removed at the beginning.

Plot name	16S	18S	*trn*L	ITS	COI	COI-spun	seedlings	trees	inverts	birds
Plot1	6	8	2	3	2	4	5	4	8	2
Plot2	8	5	8	2	8	1	6	3	6	10
Plot3	10	7	6	6	5	3	4	7	4	1
Plot4	1	2	5	1	4	2	2	6	2	9
Plot5	2	1	3	7	1	8	1	1	7	8
Plot6	7	3	1	5	6	10	10	10	5	7
Plot7	4	9	4	4	9	9	8	5	NA*	3
Plot8	5	10	9	8	7	5	7	9	NA*	6
Plot9	9	6	10	10	10	7	3	2	1	5
Plot10	3	4	7	9	3	6	9	8	3	4

* NA: invertebrate data is missing for Plots 7 and 8.

Table SA12 Means and standard deviations of Table SA11 between eDNA datasets for ranking plots to minimize the loss of overall γ diversity among the remaining plots.

	eDNA	Traditional	Traditional No Birds	All	All No Birds
Plot1	4.2 ± 2.40	4.8 ± 2.50	5.7 ± 2.08	4.4 ± 2.32	4.7 ± 2.29
Plot2	5.3 ± 3.20	6.2 ± 2.87	5.0 ± 1.73	5.7 ± 2.95	5.2 ± 2.68
Plot3	6.2 ± 2.32	4.0 ± 2.45	5.0 ± 1.73	5.3 ± 2.50	5.8 ± 2.11
Plot4	2.5 ± 1.64	4.8 ± 3.40	3.3 ± 2.31	3.4 ± 2.59	2.8 ± 1.79
Plot5	3.7 ± 3.08	4.2 ± 3.77	3.0 ± 3.46	3.9 ± 3.18	3.4 ± 3.00
Plot6	5.3 ± 3.14	8.0 ± 2.45	8.3 ± 2.89	6.4 ± 3.06	6.3 ± 3.24
Plot7	6.5 ± 2.74	5.3 ± 2.52	6.5 ± 2.12	6.1 ± 2.57	6.5 ± 2.45
Plot8	7.3 ± 2.07	7.3 ± 1.53	8.0 ± 1.41	7.3 ± 1.80	7.5 ± 1.85
Plot9	8.7 ± 1.75	2.8 ± 1.71	2.0 ± 1.00	6.3 ± 3.47	6.4 ± 3.64
Plot10	5.3 ± 2.42	6.0 ± 2.94	6.7 ± 3.21	5.6 ± 2.50	5.8 ± 2.59

Table SA13 Spearman correlations and their significance in parentheses of Table SA11 between eDNA datasets for ranking plots to minimize the loss of overall γ diversity among the remaining plots.

	16S	18S	*trn*L	ITS	COI	COI-spun	seedlings	trees	inverts
18S	0.39 (0.26)								
trnL	0.28 (0.43)	0.31 (0.39)							
ITS	0.18 (0.63)	0.12 (0.76)	0.45 (0.19)						
COI	0.50 (0.14)	0.47 (0.18)	0.54 (0.11)	0.12 (0.76)					
COI-spun	-0.13 (0.73)	-0.067 (0.86)	-0.38 (0.28)	0.45 (0.19)	0.10 (0.79)				
seedlings	0.13 (0.73)	0.32 (0.37)	-0.12 (0.76)	0.079 (0.84)	0.27 (0.45)	0.36 (0.31)			
trees	0.0061 (1.0)	0.19 (0.61)	-0.10 (0.79)	0.055 (0.89)	0.018 (0.97)	0.12 (0.76)	0.70 (0.031)		
inverts	-0.12 (0.79)	0.095 (0.84)	-0.57 (0.15)	-0.31 (0.46)	-0.52 (0.20)	0.048 (0.93)	0.12 (0.79)	-0.19 (0.66)	
birds	-0.33 (0.35)	-0.60 (0.073)	0.091 (0.81)	-0.30 (0.41)	0.042 (0.92)	-0.16 (0.66)	-0.19 (0.61)	-0.18 (0.63)	0 (1.0)

Table SA14 Ranking sampling plots by removing plots sequentially to maximize the resulting effective β diversity among the remaining plots from Figure SA16. 1 is the most important and is removed last, 10 is the least important and is removed at the beginning.

Plot name	16S	18S	*trn*L	ITS	COI	COI-spun	seedlings	trees	inverts	birds
Plot1	2	2	3	2	4	2	2	2	2	3
Plot2	7	9	10	8	8	6	8	7	7	8
Plot3	4	4	5	3	5	8	5	5	3	2
Plot4	3	5	2	6	2	7	6	6	5	5
Plot5	10	10	8	10	10	10	10	10	8	1
Plot6	9	7	6	5	7	4	4	3	6	7
Plot7	6	1	1	7	1	3	7	9	NA*	4
Plot8	8	6	9	4	9	5	9	8	NA*	6
Plot9	5	8	4	9	3	9	3	1	1	10
Plot10	1	3	7	1	6	1	1	4	4	9

* NA: invertebrate data is missing for Plots 7 and 8.

Table SA15 Means and standard deviations of Table SA14 between eDNA datasets for ranking plots to maximize effective β diversity among the remaining plots.

	eDNA	Traditional	Traditional No Birds	All	All No Birds
Plot1	2.5 ± 0.837	2.2 ± 0.500	2.0 ± 0	2.4 ± 0.699	2.3 ± 0.707
Plot2	8.0 ± 1.41	7.5 ± 0.577	7.3 ± 0.577	7.8 ± 1.14	7.8 ± 1.20
Plot3	4.8 ± 1.72	3.8 ± 1.50	4.3 ± 1.15	4.4 ± 1.65	4.7 ± 1.50
Plot4	4.2 ± 2.14	5.5 ± 0.577	5.7 ± 0.577	4.7 ± 1.77	4.7 ± 1.87
Plot5	9.7 ± 0.816	7.2 ± 4.27	9.3 ± 1.15	8.7 ± 2.83	9.6 ± 0.882
Plot6	6.3 ± 1.75	5.0 ± 1.83	4.3 ± 1.53	5.8 ± 1.81	5.7 ± 1.87
Plot7	3.2 ± 2.71	6.7 ± 2.52	8.0 ± 1.41	4.3 ± 3.04	4.4 ± 3.25
Plot8	6.8 ± 2.14	7.7 ± 1.53	8.5 ± 0.707	7.1 ± 1.90	7.2 ± 1.98
Plot9	6.3 ± 2.66	3.8 ± 4.27	1.7 ± 1.15	5.3 ± 3.43	4.8 ± 3.19
Plot10	3.2 ± 2.71	4.5 ± 3.32	3.0 ± 1.73	3.7 ± 2.87	3.1 ± 2.32

Table SA16 Spearman correlations and their significance in parentheses of Table SA14 between eDNA datasets for ranking plots to maximize effective β diversity among the remaining plots.

	16S	18S	trnL	ITS	COI	COI-spun	seedlings	trees	inverts
18S	0.67 (0.039)								
trnL	0.47 (0.18)	0.65 (0.049)							
ITS	0.64 (0.054)	0.68 (0.035)	0.079 (0.84)						
COI	0.60 (0.073)	0.65 (0.049)	0.93 (0.00013)	0.091 (0.81)					
COI-spun	0.44 (0.20)	0.71 (0.028)	0.15 (0.68)	0.71 (0.028)	0.20 (0.58)				
seedlings	0.75 (0.018)	0.48 (0.17)	0.38 (0.28)	0.60 (0.073)	0.45 (0.19)	0.50 (0.14)			
trees	0.52 (0.13)	0.15 (0.68)	0.26 (0.47)	0.38 (0.28)	0.33 (0.35)	0.21 (0.56)	0.88 (0.0020)		
inverts	0.62 (0.11)	0.62 (0.11)	0.67 (0.083)	0.40 (0.33)	0.76 (0.037)	0.14 (0.75)	0.74 (0.046)	0.83 (0.015)	
birds	-0.16 (0.66)	0.18 (0.63)	0.21 (0.56)	-0.0061 (1.0)	-0.067 (0.86)	-0.22 (0.54)	-0.41 (0.25)	-0.48 (0.17)	-0.33 (0.43)

Table SA17 Distance-based redundancy analysis and their ANOVA tests in each step for the eDNA biodiversity datasets (16S, 18S, *trn*L).

	All			VIF< 10	Forward	Backward
16S	Inertia	Proportion	Pr(>F)	Pr(>F)	Pr(>F)	Pr(>F)
Elevation	0.48	9.6%	0.04			
Slope	0.43	8.7%	0.06	0.008		
Aspect	0.37	7.4%	0.13			
Mean.Temp	0.42	8.5%	0.04	0.032	0.001	0.012
cos.aspect	0.45	9.1%	0.05	0.049		
sin.aspect	0.43	8.7%	0.06	0.003		
Water.Content	0.50	10.1%	0.01			
pH	0.93	18.8%	0.01	0.007	0.002	0.001
EC	0.61	12.2%	0.01			
Organic.C	0.56	11.4%	0.01			
Total.N	0.52	10.5%	0.01			
C.N.ratio	0.44	8.9%	0.05	0.126		
NO3.N	0.28	5.7%	0.28	0.433		
NH4.N	0.50	10.0%	0.03	0.132		
Olsen.P	0.67	13.5%	0.01	0.583	0.055	0.001
18S	Inertia	Proportion	Pr(>F)	Pr(>F)	Pr(>F)	Pr(>F)
Elevation	0.64	9.11%	0.01			
Slope	0.62	8.84%	0.03	0.008	0.011	0.006
Aspect	0.45	6.45%	0.17			
Mean.Temp	0.56	7.99%	0.03	0.067		0.073
cos.aspect	0.37	5.23%	0.43	0.286		
sin.aspect	0.36	5.14%	0.43	0.204		0.231
Water.Content	0.63	8.95%	0.01			
pH	0.60	8.51%	0.03	0.022		0.031
EC	0.46	6.48%	0.10			
Organic.C	0.55	7.80%	0.06			
Total.N	0.55	7.81%	0.04			
C.N.ratio	0.42	5.91%	0.20	0.034		
NO3.N	0.38	5.42%	0.40	0.763		0.352
NH4.N	0.46	6.56%	0.13	0.019		
Olsen.P	0.47	6.63%	0.12	0.359	0.056	0.002
*trn*L	Inertia	Proportion	Pr(>F)	Pr(>F)	Pr(>F)	Pr(>F)
Elevation	0.62	7.09%	0.01			
Slope	0.61	7.01%	0.01	0.001		0.001
Aspect	0.55	6.31%	0.04			
Mean.Temp	0.58	6.70%	0.01	0.01		0.01
cos.aspect	0.66	7.53%	0.01	0.001		0.001
sin.aspect	0.52	6.00%	0.07	0.01		
Water.Content	0.65	7.53%	0.01			
pH	0.70	8.06%	0.01	0.057	0.001	0.002
EC	0.60	6.88%	0.01			
Organic.C	0.65	7.42%	0.01			
Total.N	0.63	7.30%	0.01			
C.N.ratio	0.50	5.76%	0.14	0.554		
NO3.N	0.55	6.32%	0.03	0.104		
NH4.N	0.58	6.68%	0.01	0.511		
Olsen.P	0.63	7.22%	0.01	0.175	0.001	0.02

Table SA18 Distance-based redundancy analysis and their ANOVA tests in each step for the eDNA biodiversity datasets (ITS, COI-soil and COI-soil spun).

	All			VIF$<$10	Forward	Backward
ITS	Inertia	Proportion	Pr(>F)	Pr(>F)	Pr(>F)	Pr(>F)
Elevation	0.60	6.67%	0.01			
Slope	0.65	7.19%	0.01	0.001	0.002	0.002
Aspect	0.65	7.15%	0.01			
Mean.Temp	0.59	6.54%	0.01	0.054		
cos.aspect	0.57	6.36%	0.04	0.109		
sin.aspect	0.58	6.46%	0.01	0.039		0.007
Water.Content	0.70	7.72%	0.01			
pH	0.48	5.35%	0.46	0.133		
EC	0.67	7.42%	0.01			
Organic.C	0.71	7.88%	0.01			
Total.N	0.72	7.95%	0.01			
C.N.ratio	0.51	5.68%	0.16	0.158		
NO3.N	0.58	6.45%	0.02	0.226		0.122
NH4.N	0.68	7.50%	0.01	0.16		
Olsen.P	0.64	7.13%	0.01	0.039	0.004	0.022
COI	Inertia	Proportion	Pr(>F)	Pr(>F)	Pr(>F)	Pr(>F)
Elevation	0.60	6.66%	0.01			
Slope	0.58	6.37%	0.01	0.001		
Aspect	0.55	6.08%	0.01			
Mean.Temp	0.59	6.49%	0.01	0.004		0.001
cos.aspect	0.59	6.56%	0.01	0.004		
sin.aspect	0.52	5.75%	0.10	0.008		
Water.Content	0.64	7.08%	0.01			
pH	0.64	7.03%	0.01	0.043	0.001	0.003
EC	0.61	6.79%	0.01			
Organic.C	0.64	7.06%	0.01			
Total.N	0.63	6.98%	0.01			
C.N.ratio	0.53	5.84%	0.04	0.213		
NO3.N	0.57	6.29%	0.01	0.171		0.004
NH4.N	0.60	6.63%	0.01	0.263		0.006
Olsen.P	0.62	6.91%	0.01	0.194	0.001	0.048
COI-spun	Inertia	Proportion	Pr(>F)	Pr(>F)	Pr(>F)	Pr(>F)
Elevation	0.61	6.54%	0.01			
Slope	0.57	6.06%	0.04	0.004		0.003
Aspect	0.61	6.52%	0.01			
Mean.Temp	0.57	6.08%	0.02	0.035		0.022
cos.aspect	0.60	6.48%	0.01	0.012	0.001	0.014
sin.aspect	0.59	6.29%	0.01	0.005	0.011	0.007
Water.Content	0.60	6.40%	0.01			
pH	0.54	5.82%	0.07	0.058		
EC	0.51	5.43%	0.36			
Organic.C	0.53	5.73%	0.12			
Total.N	0.54	5.83%	0.07			
C.N.ratio	0.45	4.83%	0.90	0.912		
NO3.N	0.55	5.90%	0.02	0.046		0.014
NH4.N	0.53	5.68%	0.12	0.061		0.014
Olsen.P	0.51	5.49%	0.26	0.229	0.008	

Table SA19 The constrained and unconstrained inertia changes during distance-based redundancy analysis for the eDNA biodiversity datasets (16S, 18S, *trn*L, ITS, COI-soil and COI-soil spun).

	All		VIF< 10		Forward		Backward	
16S	Inertia	Proport.	Inertia	Proport.	Inertia	Proport.	Inertia	Proportion
Constrained	4.22	85%	3.04	61.3%	1.86	37.4%	1.86	37.4%
Unconstrained	0.74	15%	1.92	38.7%	3.11	62.6%	3.11	62.6%
18S	Inertia	Proport.	Inertia	Proport.	Inertia	Proport.	Inertia	Proportion
Constrained	5.86	83.4%	3.84	54.6%	1.11	15.8%	2.87	40.8%
Unconstrained	1.17	16.6%	3.19	45.4%	5.92	84.2%	4.16	59.2%
*trn*L	Inertia	Proport.	Inertia	Proport.	Inertia	Proport.	Inertia	Proportion
Constrained	7.07	81.3%	4.49	51.61%	1.33	15.3%	2.82	32.4%
Unconstrained	1.63	18.7%	4.21	48.39%	7.37	84.7%	5.88	67.6%
ITS	Inertia	Proport.	Inertia	Proport.	Inertia	Proport.	Inertia	Proportion
Constrained	7.31	81%	4.62	51.24%	1.26	14%	2.28	25.3%
Unconstrained	1.72	19%	4.40	48.76%	7.76	86%	6.74	74.7%
COI	Inertia	Proport.	Inertia	Proport.	Inertia	Proport.	Inertia	Proportion
Constrained	7.33	81%	4.56	50.49%	1.26	14%	2.74	30.3%
Unconstrained	1.71	19%	4.47	49.51%	7.78	86%	6.29	69.7%
COI-spun	Inertia	Proport.	Inertia	Proport.	Inertia	Proport.	Inertia	Proportion
Constrained	7.50	80.4%	4.71	50.49%	1.77	19%	3.34	35.8%
Unconstrained	1.83	19.6%	4.62	49.52%	7.56	81%	5.99	64.2%

Table SA20 Distance-based redundancy analysis and their ANOVA tests in each step for the plant datasets (seedlings and trees).

	All			VIF< 10	Forward	Backward
seedlings	Inertia	Proportion	Pr(>F)	Pr(>F)	Pr(>F)	Pr(>F)
Slope	0.48	14.18%	0.05	0.094		
Mean.Temp	0.54	15.96%	0.01	0.113		0.001
cos.aspect	0.43	12.69%	0.20	0.424		
sin.aspect	0.41	11.96%	0.28	0.448		
pH	0.58	17.09%	0.01	0.152	0.001	0.014
C.N.ratio	0.45	13.27%	0.10	0.816		
NO3.N	0.55	16.05%	0.01	0.456	0.005	0.002
NH4.N	0.44	12.94%	0.10			
Olsen.P	0.47	13.87%	0.06	0.772		
trees	Inertia	Proportion	Pr(>F)	Pr(>F)	Pr(>F)	Pr(>F)
Slope	0.52	17.5%	0.09	0.023		0.015
Mean.Temp	0.69	23.4%	0.01	0.03	0.002	
cos.aspect	0.48	16.4%	0.07	0.099	0.095	
sin.aspect	0.44	15.0%	0.13	0.126		
pH	0.63	21.4%	0.02	0.177		0.068
C.N.ratio	0.35	11.7%	0.33	0.253		0.014
NO3.N	0.50	16.8%	0.07	0.2		0.071
NH4.N	0.41	13.8%	0.15			

Table SA21 The constrained and unconstrained inertia changes during distance-based redundancy analysis for the plant datasets (seedlings and trees).

	All		VIF< 10		Forward		Backward	
seedlings	Inertia	Proport.	Inertia	Proport.	Inertia	Proport.	Inertia	Proportion
Constrained	3.40	100%	3.05	89.9%	1.10	32.5%	1.51	44.6%
Unconstrained	0.00	0%	0.34	10.1%	2.29	67.5%	1.88	55.4%
seedlings	Inertia	Proport.	Inertia	Proport.	Inertia	Proport.	Inertia	Proportion
Constrained	2.95	100%	2.75	93.2%	1.11	37.6%	1.80	60.9%
Unconstrained	0.00	0%	0.20	6.8%	1.84	62.4%	1.15	39.1%
